# Meta-Analysis of 28,141 Individuals Identifies Common Variants within Five New Loci That Influence Uric Acid Concentrations

**DOI:** 10.1371/journal.pgen.1000504

**Published:** 2009-06-05

**Authors:** Melanie Kolz, Toby Johnson, Serena Sanna, Alexander Teumer, Veronique Vitart, Markus Perola, Massimo Mangino, Eva Albrecht, Chris Wallace, Martin Farrall, Åsa Johansson, Dale R. Nyholt, Yurii Aulchenko, Jacques S. Beckmann, Sven Bergmann, Murielle Bochud, Morris Brown, Harry Campbell, for the EUROSPAN Consortium, John Connell, Anna Dominiczak, Georg Homuth, Claudia Lamina, Mark I. McCarthy, for the ENGAGE Consortium, Thomas Meitinger, Vincent Mooser, Patricia Munroe, Matthias Nauck, John Peden, Holger Prokisch, Perttu Salo, Veikko Salomaa, Nilesh J. Samani, David Schlessinger, Manuela Uda, Uwe Völker, Gérard Waeber, Dawn Waterworth, Rui Wang-Sattler, Alan F. Wright, Jerzy Adamski, John B. Whitfield, Ulf Gyllensten, James F. Wilson, Igor Rudan, Peter Pramstaller, Hugh Watkins, for the PROCARDIS Consortium, Angela Doering, H.-Erich Wichmann, for the KORA Study, Tim D. Spector, Leena Peltonen, Henry Völzke, Ramaiah Nagaraja, Peter Vollenweider, Mark Caulfield, for the WTCCC, Thomas Illig, Christian Gieger

**Affiliations:** 1 Institute of Epidemiology, Helmholtz Zentrum München, National Research Center for Environment and Health, Neuherberg, Germany; 2 Department of Medical Genetics, University of Lausanne, Lausanne, Switzerland; 3 Swiss Institute of Bioinformatics, Lausanne, Switzerland; 4 University Institute of Social and Preventive Medicine, Centre Hospitalier Universitaire Vaudois and University of Lausanne, Switzerland; 5 Istituto di Neurogenetica e Neurofarmacologia, Cagliari, Italy; 6 Interfaculty Institute for Genetics and Functional Genomics, Ernst-Moritz-Arndt-Universität Greifswald, Greifswald, Germany; 7 MRC Human Genetics Unit, IGMM, Western General Hospital, Crewe Road, Edinburgh, United Kingdom; 8 Department of Chronic Disease Prevention, Institute of Health and Welfare, Helsinki, Finland; 9 FIMM, Institute of Molecular Medicine, Helsinki, Finland; 10 DTR Department of Twin Research and Genetic Epidemiology, King's College London, London, United Kingdom; 11 The Diabetes Inflammation Laboratory, Cambridge Institute of Medical Research, Cambridge University, Cambridge, United Kingdom; 12 Department of Cardiovascular Medicine, University of Oxford, Oxford, United Kingdom; 13 The Wellcome Trust Centre for Human Genetics, University of Oxford, Oxford, United Kingdom; 14 Department of Genetics and Pathology, Rudbeck Laboratory, Uppsala University, Uppsala, Sweden; 15 Genetic Epidemiology Unit, Queensland Institute of Medical Research, Brisbane, Australia; 16 Department of Epidemiology, Erasmus MC Rotterdam, Rotterdam, The Netherlands; 17 Service of Medical Genetics, Centre Hospitalier Universitaire Vaudois, Lausanne, Switzerland; 18 Clinical Pharmacology Unit, Addenbrookes Hospital, University of Cambridge, Cambridge, United Kingdom; 19 Centre for Population Health Sciences, University of Edinburgh, Edinburgh, United Kingdom; 20 Glasgow Cardiovascular Research Centre, University of Glasgow, Glasgow, United Kingdom; 21 Division of Genetic Epidemiology, Department of Medical Genetics, Molecular and Clinical Pharmacology, Innsbruck Medical University, Innsbruck, Austria; 22 Oxford Centre for Diabetes, Endocrinology and Metabolism, University of Oxford, Churchill Hospital, Oxford, United Kingdom; 23 National Institute for Health Research, Oxford Biomedical Research Centre, University of Oxford, Headington, Oxford, United Kingdom; 24 Institute of Human Genetics, Helmholtz Zentrum München, National Research Center for Environment and Health, Neuherberg, Germany; 25 Genetics Division, GlaxoSmithKline, King of Prussia, Pennsylvania, United States of America; 26 The William Harvey Research Institute, Barts and The London School of Medicine and Dentistry, Queen Mary University of London, London, England; 27 Institut für Klinische Chemie und Laboratoriumsmedizin, Ernst-Moritz-Arndt-Universität Greifswald, Greifswald, Germany; 28 Cardiovascular Sciences, University of Leicester, Glenfield Hospital, Leicester, United Kingdom; 29 Laboratory of Genetics, Intramural Research Program, National Institute on Aging, National Institutes of Health, Baltimore, Maryland, United States of America; 30 Department of Medicine and Internal Medicine, Centre Hospitalier Universitaire Vaudois, Lausanne, Switzerland; 31 Genome Analysis Centre, Institute for Experimental Genetics, Helmholtz Zentrum München, National Research Center for Environment and Health, Neuherberg, Germany; 32 Croatian Centre for Global Health, Faculty of Medicine, University of Split, Split, Croatia; 33 Gen-Info, Zagreb, Croatia; 34 Institute of Genetic Medicine, European Academy Bozen/Bolzano (EURAC), Bolzano, Italy; 35 Affiliated Institute of the University Lübeck, Department of Neurology, Central Hospital, Bolzano, Italy; 36 IBE, Chair of Epidemiology, University of Munich, Germany; 37 The Wellcome Trust Sanger Institute, Wellcome Trust Genome Campus, Hinxton, Cambridge, United Kingdom; 38 Institute for Community Medicine, Ernst-Moritz-Arndt-Universität Greifswald, Greifswald, Germany; University of Alabama at Birmingham, United States of America

## Abstract

Elevated serum uric acid levels cause gout and are a risk factor for cardiovascular disease and diabetes. To investigate the polygenetic basis of serum uric acid levels, we conducted a meta-analysis of genome-wide association scans from 14 studies totalling 28,141 participants of European descent, resulting in identification of 954 SNPs distributed across nine loci that exceeded the threshold of genome-wide significance, five of which are novel. Overall, the common variants associated with serum uric acid levels fall in the following nine regions: *SLC2A9* (p = 5.2×10^−201^), *ABCG2* (p = 3.1×10^−26^), *SLC17A1* (p = 3.0×10^−14^), *SLC22A11* (p = 6.7×10^−14^), *SLC22A12* (p = 2.0×10^−9^), *SLC16A9* (p = 1.1×10^−8^), *GCKR* (p = 1.4×10^−9^), *LRRC16A* (p = 8.5×10^−9^), and near *PDZK1* (p = 2.7×10^−9^). Identified variants were analyzed for gender differences. We found that the minor allele for rs734553 in *SLC2A9* has greater influence in lowering uric acid levels in women and the minor allele of rs2231142 in *ABCG2* elevates uric acid levels more strongly in men compared to women. To further characterize the identified variants, we analyzed their association with a panel of metabolites. rs12356193 within *SLC16A9* was associated with DL-carnitine (p = 4.0×10^−26^) and propionyl-L-carnitine (p = 5.0×10^−8^) concentrations, which in turn were associated with serum UA levels (p = 1.4×10^−57^ and p = 8.1×10^−54^, respectively), forming a triangle between SNP, metabolites, and UA levels. Taken together, these associations highlight additional pathways that are important in the regulation of serum uric acid levels and point toward novel potential targets for pharmacological intervention to prevent or treat hyperuricemia. In addition, these findings strongly support the hypothesis that transport proteins are key in regulating serum uric acid levels.

## Author Summary

Elevated serum uric acid levels cause gout and are a risk factor for cardiovascular disease and diabetes. The regulation of serum uric acid levels is under a strong genetic control. This study describes the first meta-analysis of genome-wide association scans from 14 studies totalling 28,141 participants of European descent. We show that common DNA variants at nine different loci are associated with uric acid concentrations, five of which are novel. These variants are located within the genes coding for organic anion transporter 4 (*SLC22A11*), monocarboxylic acid transporter 9 (*SLC16A9*), glucokinase regulatory protein (*GCKR*), Carmil (*LRRC16A*), and near PDZ domain-containing 1 (*PDZK1*). Gender-specific effects are shown for variants within the recently identified genes coding for glucose transporter 9 (*SLC2A9*) and the ATP-binding cassette transporter (*ABCG2*). Based on screening of 163 metabolites, we show an association of one of the identified variants within *SLC16A9* with DL-carnitine and propionyl-L-carnitine. Moreover, DL-carnitine and propionyl-L-carnitine were strongly correlated with serum UA levels, forming a triangle between SNP, metabolites and UA levels. Taken together, these associations highlight pathways that are important in the regulation of serum uric acid levels and point toward novel potential targets for pharmacological intervention to prevent or treat hyperuricemia.

## Introduction

Uric acid (UA) is the final catabolic, heterocyclic purine derivative resulting from the oxidation of purines in humans. Due to the loss of hepatic uricase activity during human evolution, UA is excreted as such and is not further metabolized into carbon dioxide and ammonia. A major mechanism underlying hyperuricemia is impaired renal excretion of urate. Most notably, UA is causally involved in the pathogenesis of gouty arthritis that results from deposition of monosodium urate crystals in the joints [1]. Increased UA concentrations have been implicated in cardiovascular disease for more than five decades [2]. In addition, elevated urate is associated with obesity, blood pressure and insulin resistance, and consequently with the metabolic syndrome and type 2 diabetes [2],[3]. However, UA also has a positive role as an antioxidant, and is correlated with longevity in mammals [4]. Thus, human physiology is especially sensitive to the precise range of UA levels.

Besides environmental factors, there is evidence for a strong genetic influence upon serum UA concentrations, with heritability estimates of up to 73% [5]. Recently, genome-wide association (GWA) studies have identified single nucleotide polymorphisms (SNPs) in the *SLC2A9* gene (solute carrier family 2, member 9 gene), a putative glucose transporter, which are strongly associated with serum UA concentrations and gout [6]–[9]. This novel gene locus functions as a high-capacity urate transporter in humans [8],[10]. This emphasises the power of GWA studies in expanding our understanding at the molecular level of disease mechanisms and in pointing to innovative therapeutic strategies.

The power of GWA studies to detect common variants with modest effects directly depends on the size of the study group. Therefore, the present study sought to detect novel genetic variants related to serum UA levels by conducting a meta-analysis of GWA findings from 14 studies (BRIGHT, CoLaus, CROATIA, Health 2000, KORA F3, KORA S4, ORCADES, PROCARDIS, NSPHS, SardiNIA, SHIP, SSAGA, MICROS, and TwinsUK) totalling 28,141 participants. In addition, the meta-analysis was performed independently on sex specific GWA results to address the pronounced gender differences in the regulation of UA concentrations that have previously been reported [1],[6]. Identified variants were further analyzed for association with metabolite profiles.

## Results

**Figure 1:**
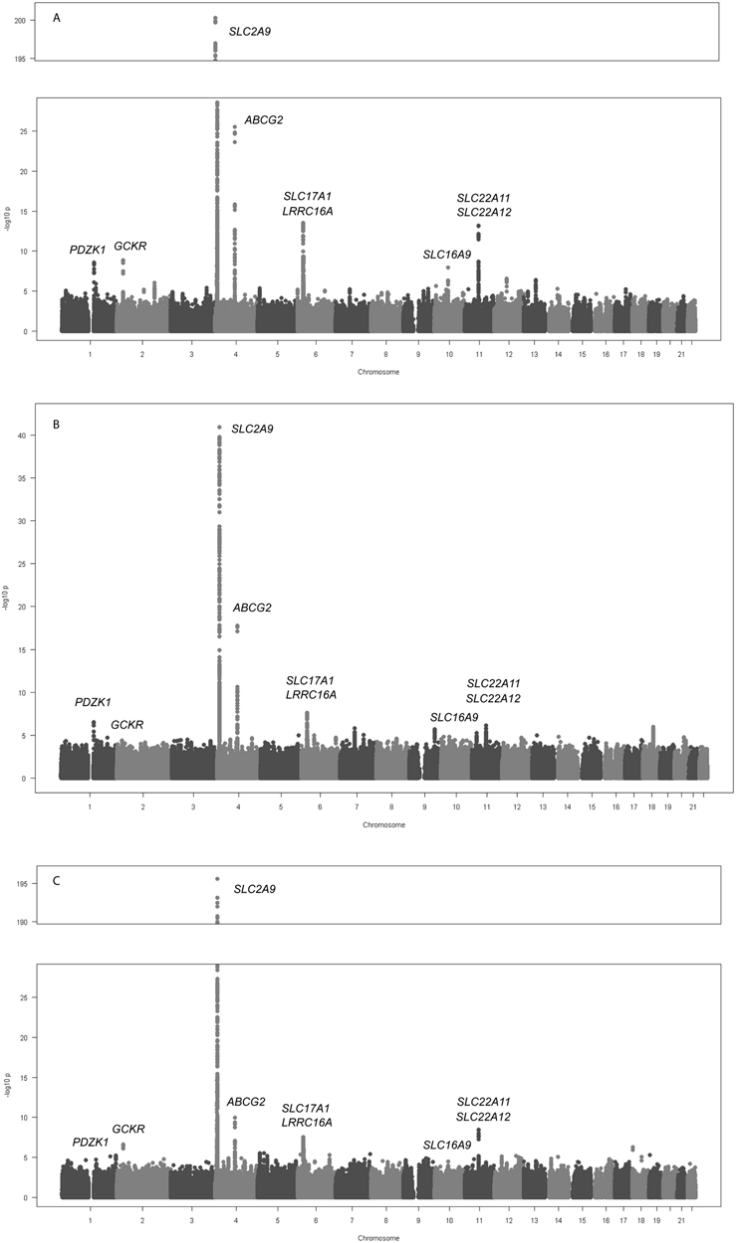
Genome-wide association results. Manhattan plots showing significance of association of all SNPs in the meta-analysis for (A) men and women combined, (B) men only, and (C) women only. SNPs are plotted on the *x*-axis according to their position on each chromosome against association with uric acid concentrations on the *y*-axis (shown as −log_10_ p-value).

The sample size and participant characteristics for each participating study are shown in Table S1. Meta-analysis of GWA data of 28,141 individuals of European ancestry yielded 954 SNPs (full list is provided in Table S4) that exceeded the genome-wide significance threshold of 5×10^−8^ (Figure 1A).

**Figure 2:**
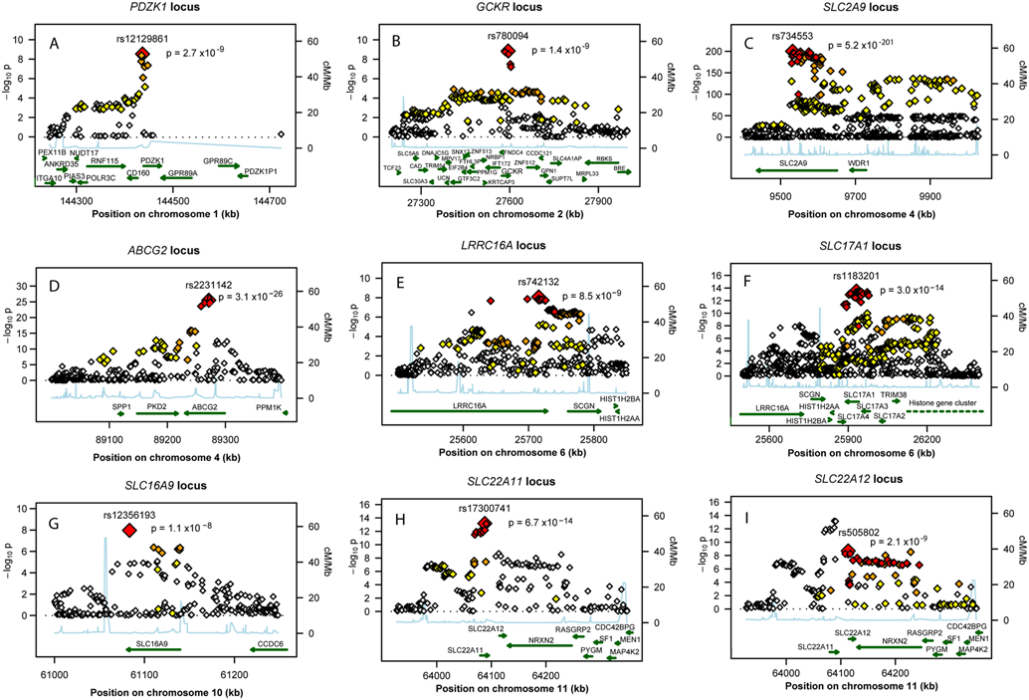
Regional association plots of nine urate loci. P-value plots showing the association signals in the region of (A) *PDZK1* on chromosome 1, (B) *GCKR* on chromosome 2, (C) *SLC2A9* on chromosome 4, (D) *ABCG2* on chromosome 4, (E) *LRRC16A* on chromosome 6, (F) *SLC17A1* on chromosome 6, (G) *SLC16A9* on chromosome 10, (H) *SLC22A11* on chromosome 11, and (I) *SLC22A12* on chromosome 11. −log_10_ p-values are plotted as a function of genomic position (NCBI Build 36). Large diamonds in red indicate the most significant SNP in the region while other SNPs in the region are given as colour-coded smaller diamonds. Red diamonds indicate high correlation with the lead SNP (r2>0.8), orange diamonds indicate moderate correlation with the most significant SNP (0.5<r2<0.8), yellow indicates markers in weak correlation with the most significant SNP (0.2<r2<0.5), white indicates no correlation with the most significant SNP (r2<0.2). Estimated recombination rates (HapMap Phase II) are given in light blue, genes as well as the direction of transcription (NCBI) are displayed by green bars.

Those SNPs cluster around nine loci (Table 1), four of which are well known regulators of serum UA levels: *SLC2A9* (p = 5.2×10^−201^), *ABCG2* (p = 3.1×10^−26^), *SLC17A1* (p = 3.0×10^−14^), and *SLC22A12* (p = 2.0×10^−9^). The first, *SLC2A9*, was identified in previous GWA scans (Figure 2C) [6]–[9]. A total of 788 SNPs reached the genome-wide significance threshold at the *SLC2A9* locus. The strongest associated marker was the intronic SNP rs734553 (p = 5.2×10^−201^, Table 1), which is in high linkage disequilibrium (r^2^ = 0.88) with the missense mutation rs16890979 previously described [11]. The second locus was on chromosome 4q22, harbouring the *ABCG2* gene (Figure 2D). In accordance with previous results, the strongest observed association was at rs2231142 (p = 3.1×10^−26^, Table 1), a coding SNP leading to a glutamine-to-lysine amino acid change at position 141 [11]. The third previously implicated locus influencing UA levels was on chromosome 6p23-p21.3, which contains the *SLC17A3* gene (Figure 2F) [11]. The top associated marker was SNP rs1183201 (p = 3.0×10^−14^, Table 1), intronic of *SLC17A1*, but the association signal encompassed a larger region including the *SLC17A1*, *SLC17A3*, *SLC17A4* genes and downstream to *HIST1H4C*, in agreement with the linkage disequilibrium at this locus. SNP rs1183201 is in high linkage disequilibrium (r^2^ = 0.97) with rs1165205, a SNP intronic of *SLC17A3* gene identified by a previous GWA scan [11].

**Table 1:** Nine loci associated with uric acid concentrations.

Loci	SNP	Chr*	Position (bp)	Allele		Frequency (Effect allele)	All individuals				Explained variability
				Effect	Other		N	beta	[95% CI]	p-value	
*PDZK1*	rs12129861	1	144437046	A	G	46.40%	25627	−0.062	[−0.083; −0.042]	2.68E-09	0.19%
*GCKR*	rs780094	2	27594741	T	C	41.70%	27991	0.052	[0.035; 0.068]	1.40E-09	0.13%
*SLC2A9*	rs734553	4	9532102	T	G	76.81%	27817	0.315	[0.294; 0.335]	5.22E-201	3.53%
*ABCG2*	rs2231142	4	89271347	T	G	10.77%	23622	0.173	[0.141; 0.205]	3.10E-26	0.57%
*LRRC16A*	rs742132	6	25715550	A	G	69.57%	27923	0.054	[0.036; 0.072]	8.50E-09	0.12%
*SLC17A1*	rs1183201	6	25931423	A	T	48.24%	27908	−0.062	[−0.078; −0.459]	3.04E-14	0.19%
*SLC16A9*	rs12356193	10	61083359	A	G	82.68%	23559	0.078	[0.051; 0.105]	1.07E-08	0.17%
*SLC22A11*	rs17300741	11	64088038	A	G	51.06%	27727	0.062	[0.046; 0.078]	6.68E-14	0.19%
*SLC22A12*	rs505802	11	64113648	T	C	69.83%	27967	−0.056	[−0.074; −0.038]	2.04E-09	0.13%

*: Chromosome.

Shown is the most significant SNP for each independent locus associated (p<5×10^−8^) with uric acid concentrations on meta-analysis in the complete dataset. Position is given for NCBI Build 36. Effect estimates result from additive linear regression on Z-scores of uric acid concentrations. P-values have been combined weighting by the inverse variance. The effect allele is the allele to which the beta (effect) estimate refers.

Among the novel loci identified, the strongest was on chromosome 11q13. One locus was localized upstream and within the *SLC22A11* gene, and represented by SNP rs17300741 (p = 6.7×10^−14^, Table 1, Figure 2H). The second signal was SNP rs505802 (p = 2.0×10^−9^), representative of all associated markers falling within and downstream the extensively studied *SLC22A12* gene coding for URAT1 (Figure 2I). The p-value plot as well as the LD block structure (r^2^ = 0.09) suggested two nearby but independently associated regions, which was verified in multiple regression analysis (Table S5).

The second novel region was on chromosome 2p23.3-p23.2 (Figure 2B). The most significant SNP in this region was SNP rs780094 (p = 1.4×10^−9^), intronic of *GCKR*, a glucokinase regulator protein recently associated with several quantitative traits including the regulation of triglycerides levels [12]. We also identified genome-wide significant association on chromosome 1q21 (Figure 2A). The top ranking SNP in this region was rs12129861 (p = 2.7×10^−9^, Table 1), located 2 kb upstream of *PDZK1* coding for PDZ domain-containing 1 reported to interact with URAT1 [13]. The fourth newly detected region was found on chromosome 6p22.2 (Figure 2E), where the association signal spans two genes, *LRRC16A* and *SCGN*, within one highly preserved LD block. The strongest p-value was observed for the SNP rs742132, located within an intron of *LRRC16A* (p = 8.5×10^−9^, Table 1). Independence of the *LRRC16A* and the *SLC17A1* loci (r^2^ = 0.07) was verified in multiple regression analysis. P-values and effect estimates only slightly changed between single SNP analysis and multiple regression analysis (Table S5). Finally, we also observed some evidence of association on chromosome 10q21.3 (Figure 2G). One SNP within *SLC16A9*, rs12356193, reached genome-wide significance (p = 1.1×10^−8^). However, there were several additional SNPs within this gene with borderline significance, supporting the hypothesis that this locus may be a true signal rather than a false positive result.

### Sex-Stratified Meta-Analysis Identifies Male and Female Specific Variants

We have also performed a meta-analysis of sex specific GWA results using all 14 studies (12,328 males, 15,813 females). Although the results did not show any additional genome-wide significant locus (Figure 1B and 1C), we were able to query which of the aforementioned SNPs have sex-specific effects on serum UA levels (Table 2). For the *SLC2A9* gene, we found that in males the top ranking SNP was still rs734553 (p = 1.1×10^−41^), while for women it was the nearby intronic SNP rs12498742 (p = 2.4×10^−196^). Supporting previously reported results, we found for both markers that the effect size of the minor allele observed in women was twice the effect size observed in men (p<3.8×10^−17^, Table 2) [6]. The minor allele of rs2231142 in the *ABCG2* gene showed a greater effect size in men compared to women (p = 0.01, Table 2). Similar, the effect size of the most significant SNP for males in the *ABCG2* gene locus, rs2199936, was greater in men compared to women (p = 0.008, Table 2). The effect sizes of the other SNPs were comparable in men and women (Table 2).

**Table 2:** Gender specific association results at the nine loci.

Loci	SNP	Chr*	Position (bp)	Effect Allele	Men				Women				Difference	
					N	beta	[95% CI]	p-value	N	beta	[95% CI]	p-value	Δ beta (men - women)	p-value
*PDZK1*	rs12129861	1	144437046	A	11888	−0.080	[−0.108; −0.048]	3.68E-07	13739	−0.047	[−0.075; −0.019]	9.10E-04	−0.033	0.140
*PDZK1*	rs1471633	1	144435096	A	12225	0.072	[0.044; 0.099]	2.94E-07	14289	0.0403	[0.016; 0.064]	1.10E-03	0.031	0.094
*GCKR*	rs780094	2	27594741	T	12255	0.050	[0.023; 0.077]	3.05E-04	15736	0.055	[0.034; 0.077]	3.11E-07	−0.005	0.744
*GCKR*	rs780093	2	27596107	T	12243	0.047	[0.020; 0.074]	6.18E-04	15751	0.056	[0.035; 0.076]	2.30E-07	−0.009	0.617
*SLC2A9*	rs734553	4	9532102	T	12178	0.220	[0.188; 0.252]	1.13E-41	15639	0.397	[0.371; 0.423]	1.05E-192	−0.177	3.8E-17
*SLC2A9*	rs12498742	4	9553150	A	12274	0.208	[0.176; 0.239]	1.50E-38	15761	0.395	[0.369; 0.420]	2.36E-196	−0.187	2.1E-19
*ABCG2*	rs2231142	4	89271347	T	10324	0.221	[0.171; 0.270]	2.25E-18	13298	0.138	[0.096; 0.181]	1.13E-10	0.083	0.013
*ABCG2*	rs2199936	4	89264355	A	10323	0.222	[0.173; 0.272]	1.65E-18	13218	0.133	[0.091; 0.176]	6.85E-10	0.089	0.008
*LRRC16A*	rs742132	6	25715550	A	12235	0.062	[0.033; 0.091]	2.68E-05	15688	0.048	[0.024; 0.071]	8.14E-05	0.014	0.449
*SLC17A1*	rs1183201	6	25931423	A	12206	−0.076	[−0.103; −0.049]	2.52E-08	15702	−0.055	[−0.075; −0.036]	4.48E-08	−0.021	0.224
*SLC17A1*	rs9393672	6	25950584	T	12252	−0.074	[−0.101; −0.047]	6.22E-08	15738	−0.056	[−0.076; −0.036]	2.77E-08	−0.018	0.296
*SLC17A1*	rs942379	6	25957599	A	12215	−0.076	[−0.103; −0.049]	2.24E-08	15686	−0.054	[−0.074; −0.034]	1.01E-07	−0.022	0.198
*SLC16A9*	rs12356193	10	61083359	A	10315	0.089	[0.047; 0.131]	3.57E-05	13244	0.073	[0.039; 0.108]	3.29E-05	0.016	0.582
*SLC22A11*	rs17300741	11	64088038	A	12120	0.066	[0.039; 0.093]	1.50E-06	15607	0.060	[0.040; 0.080]	3.60E-09	0.006	0.735
*SLC22A11*	rs2078267	11	64090690	T	12259	−0.066	[−0.093; −0.039]	1.62E-06	15750	-0.061	[−0.081; −0.041]	3.22E-09	−0.033	0.757
*SLC22A12*	rs505802	11	64113648	T	12232	−0.073	[−0.102; −0.044]	7.22E-07	15735	-0.047	[−0.070; −0.023]	1.02E-04	−0.026	0.161

*: Chromosome.

Shown are the gender-specific loci for the most significant SNP at the nine associated loci. Positions are given according to NCBI Build 36. Effect estimates result from additive linear regression on Z-scores of uric acid concentrations when only males (or females) were considered for the analysis. P-values have been calculated using weighting by the inverse variance. The effect allele is the allele to which the beta (effect) estimate refers. When different from the main meta-analysis, the most associated marker in males (females) is also listed.

### Association of Identified Variants with Metabolite Profiles

To further characterize the identified variants, we analyzed their association with a panel of 163 metabolites measured in the KORA F4 survey. After correction for multiple testing, one SNP within *SLC16A9*, rs12356193, was associated with DL-carnitine concentrations (β = −3.58, p = 4.0×10^−26^), which in turn were associated with serum UA levels (β = 0.06, p = 1.4×10^−57^). In addition, this SNP was associated with propionyl-L-carnitine (β = −0.06, p = 5.0×10^−8^). Similar to DL-carnitine, propionyl-L-carnitine concentrations were also strongly associated with serum UA levels (β = 1.78, p = 8.1×10^−54^), forming a triangle between SNP, metabolites and UA levels. None of the other SNPs were significantly associated with the measured metabolites.

## Discussion

Based on meta-analysis of GWA studies including 28,141 individuals, we have mapped 5 novel loci and confirmed 4 previously implicated loci that influence serum UA levels. Altogether, these associations highlight biological pathways that are important in the regulation of urate concentrations and may point to novel targets for pharmacological interventions to prevent or treat hyperuricemia.

A genome-wide significant p-value was observed for one SNP within *SLC16A9* gene locus, coding for monocarboxylic acid transporter 9 (MCT9). This is a member of the monocarboxylate co-transporter family that has been demonstrated to catalyze transport of monocarboxylic acids across cell membranes [14]. MCT9 is expressed in various tissues including the kidney [15]. As other sodium monocarboxylate transporters have been found to influence urate in knockout models this MCT9 isoform might be a sodium-dependent transporter in the kidney. The second newly identified locus was *GCKR* (glucokinase regulatory protein) a regulator of glucokinase, the first glycolytic enzyme which serves as a glucose sensor, responsible for glucose phosphorylation in the liver. Recently, GWA studies for type 2 diabetes identified the same rs780094 SNP as a potential marker for modulation of triglyceride levels [16]. Meanwhile, *GCKR* polymorphisms were also shown to be associated with metabolic traits like fasting glucose and, modestly, type 2 diabetes [12],[17],[18]. Several potential mechanisms have been proposed to link serum UA concentrations with metabolic traits. Exogenous insulin decreases renal sodium and urate excretion [19]. Furthermore, renal clearance of UA is inversely related to the degree of insulin resistance [20]. Finally, insulin resistance is thought to be accompanied by impaired oxidative phosphorylation in hepatic mitochondria, leading to increased concentrations of co-enzyme A esters and thus to increased systemic adenosine concentrations [21]. Increased adenosine, in turn, may result in renal retention of sodium, urate, and water [21],[22]. This provides a putative mechanism for hyperuricaemia via both the break down of adenosine to urate and increased renal urate retention [21],[22].

We also found evidence for association in a region containing two genes, *LRRC16A* and *SCGN*. The strongest association was located within *LRRC16A* coding for CARMIL. This large protein is most abundant in kidney and epithelial tissues and serves as an inhibitor of the heterodimeric actin capping protein (CP), an essential element of the actin cytoskeleton which binds to the barbed ends of actin filaments and regulates their polymerization [23]. The multiple biochemical functions associated with CARMIL raise many possibilities for its mechanism of action in cells, but the relation of CARMIL to UA concentration is thus far unclear. The nearby *SCGN* is coding for Secretagogin, a calcium-binding protein selectively expressed in neuroendocrine tissue and pancreatic beta-cells. The function of Secretagogin is unknown, but it has been suggested to influence calcium influx and insulin secretion [24].

We also demonstrated association of SNPs in *SLC22A11* and *SLC22A12* with UA concentrations. *SLC22A12* encodes the extensively studied URAT1, a member of the organic anion transporter (OAT) family [25]. URAT1, a well known candidate gene for UA accumulation/transport, mediates the non-voltage-dependent exchange of urate for several organic anions [1]. *SLC22A11* codes for OAT4, an OAT isoform which, like URAT1, is localized at the apical membrane of the proximal tubules. OAT4 serves as an organic anion–dicarboxylate exchanger, which mediates urate transport across the apical membrane of kidney [26],[27]. In combination with these findings, we also identified genome-wide significant association of SNPs in and upstream of *PDZK1*, coding for PDZ domain containing 1, a scaffolding protein reported to interact with OAT4, URAT1 and NTP1 (*SLC17A1*) via their C-terminal PDZ motifs [13],[28]. It has been proposed that the PDZ scaffold may form a bidirectional transport system by linking URAT1 (reabsorption) and NPT1 (secretion) leading to a functional complex responsible for the balanced urate transport regulation at the apical membrane of renal proximal tubules [1],[28].

In accordance with previous genome-wide studies, the strongest effect on serum UA concentrations was detected for *SLC2A9*, [6]–[9] coding for GLUT9, which has been shown to be strongly associated with hyperuricemia and gout and to serve as a high-capacity urate transporter in humans [8],[10]. Additional confirmed loci include *ABCG2* and *SLC17A1* [11]. *ABCG2* is a member of the ATP-binding cassette (ABC) superfamily of membrane transporters, while the *SLC17A1* locus, located directly downstream of the recently identified *SLC17A3* locus (NPT4), encodes NPT1 (renal sodium phosphate transport protein 1). The human NPT1 is localized at the apical membrane of renal proximal tubules and serves as a voltage-driven UA transporter in model systems [28].

Although several of the SNPs associated with uric acid concentrations in this meta-analysis are located within genes that are plausible candidates for influencing uric acid concentrations, our association approach is not able to identify underlying genes or mechanisms in the regions of association signals. Therefore, other genes might be responsible for the observed associations and functional studies are warranted to identify the causal variants and provide insights in the underlying biological mechanisms.

Pronounced gender differences in the regulation of serum UA concentrations have been reported for both humans and animals [1],[6]. In line with our previous findings, [6] the strongest gender-specific effect was observed for the minor allele of rs734553 (*SLC2A9*), resulting in a 2-fold larger effect size on serum UA concentrations in women compared to men. For *ABCG2*, the effect of the minor allele of rs2231142 demonstrated a larger effect on UA concentrations in men compared to women. For the other loci, effect sizes did not significantly differ by gender.

The rapidly evolving field of metabolomics aims at a comprehensive measurement of endogenous metabolites in a cell or body fluid [29]. Based on screening of 163 metabolites, we have observed an association of one of the identified variants, rs12356193 within *SLC16A9*, with DL-carnitine and propionyl-L-carnitine. Moreover, DL-carnitine and propionyl-L-carnitine were strongly correlated with serum UA levels, forming a triangle between SNP, metabolites and UA levels. Carnitine is acquired from diet and endogenous biosynthesis. Its primary function is in the transport of long chain fatty acids. After strenuous physical exercise, both acylcarnitine and UA levels increase in the serum of healthy humans [30]. In spontaneously hypertensive rats, L-carnitine decreases serum UA levels and the age-dependent rise in serum UA [31],[32]. Kidneys absorb 95% of carnitine from the glomerular filtrate via an active Na^+^-dependent transport mechanism [33]. Impairment of this reabsorptive function can lead to carnitine deficiency, in which hyperuricemia may be present because carnitine competes for renal tubular excretion [34]. Although experimental data are few, currently available data suggest that urinary acylcarnitine, which reflects the balance between dietary intake of carnitine and renal excretion, may be linked to serum UA via oxidative stress pathways [35]. Given that palmitoyl carnitine inhibits binding of Ca^2+^ channel ligands to rat brain cortical membranes and to inhibit voltage-activated Ca^2+^ channel currents, acylcarnitines may also have direct influences on MCT9 [36].

Overall, serum UA concentrations are mainly determined by the balance between urate production and renal excretion. We have identified nine loci that are associated with serum UA levels and six of them harbor genes that code for renal transport proteins. Most notably, five of these transport proteins belong to the family–and moreover, to one phylogenetic cluster within this family [37]. These findings strongly support the hypothesis that genetic variation in urate transport proteins are the key influences upon regulation of serum UA levels in humans.

## Materials and Methods

### Study Participants

The present meta-analysis combined data from 14 GWA scans: British Genetics of Hypertension (BRIGHT), Cohorte Lausannoise (CoLaus), Vis Island Isolate Study (CROATIA), Health 2000 cohort (Health 2000), two surveys of the Cooperative Health Research in the Region of Augsburg (KORA F3, KORA S4), Orkney Complex Disease Study (ORCADES), Precocious Coronary Artery Disease (PROCARDIS), Northern Swedish Population Health Study (NSPHS), SardiNIA Study of Aging (SardiNIA), Study of Health in Pomerania (SHIP), Semi-Structured Assessment for Genetics of Alcoholism (SSAGA), Microisolates in South Tyrol (MICROS), and UK Adult Twin Register (TwinsUK). Altogether, the meta-analysis comprises 28,141 individuals (12,328 males, 15,813 females) of European ancestry with measured serum UA concentrations (Table S1). Approval was obtained by local ethic committees for all studies and informed consent was given from the study participants. A detailed individual description of study designs is provided in Text S1.

### Genome-Wide Genotyping and Imputation

Six different platforms/arrays were used for genotyping: the Affymetrix 500 K GeneChip array (4 cohorts, n = 13,103), the Affymetrix 6.0 GeneChip array (2 cohorts, n = 5,901), Illumina HumanHap 300 (5 cohorts, n = 3,609), Illumina Human 610 K Beadchip (1 cohort, n = 2,212), Illumina HumanHap 300-Duo (1 cohort, n = 2,113), and Illumina Human 1 M beadchip (1 cohort, n = 1,203). Imputation of allele dosage of SNPs typed in the HapMap CEU population was performed using either MACH [38] or IMPUTE [39] with parameters and pre-imputation filters as specified in Table S2. All SNPs with a minor allele frequency <0.01 were excluded from analysis. SNPs were also excluded if the cohort-specific imputation quality as assessed by r2.hat (MACH) or .info (IMPUTE) metrics was <0.30 or <0.40, respectively. In total, up to 2,493,963 genotyped or imputed autosomal SNPs were analyzed.

### Uric Acid Measurements

Non-fasting blood samples were obtained from study participants of BRIGHT, KORA, NSPHS, SardiNIA, SHIP and SSAGA and fasting samples from those of CoLaus, PROCARDIS, CROATIA, Health 2000, MICROS, ORCADES and TwinsUK. UA analyses were carried out on fresh samples in all studies except from BRIGHT, NSPHS, CROATIA, MICROS and SSAGA, where frozen serum was used that was stored at −20°C (BRIGHT) or −70°C (NSPHS, SSAGA, CROATIA, MICROS). UA concentrations were measured using an uricase/peroxidase method (CROATIA, MICROS, NSPHS and ORCADES: DVIA1650-Autoanalyzer, Siemens Healthcare Diagnostics) or an uricase method (BRIGHT: Hitachi, Roche Diagnostics; CoLaus: uricase PGP, Roche Diagnostics; Health 2000: Thermo Fisher Scientific, Vantaa; KORA F3: URCA Flex, Dade Behring; KORA S4: UA Plus, Roche; PROCARDIS: Hitachi 917, Roche Diagnostics; SardiNIA: Bayer; SHIP: UA PAP, Boehringer; SSAGA: Hitachi 747, Boehringer; TwinsUK: Ektachem/Vitros system, Johnson & Johnson Clinical Diagnostics).

### Metabolite Measurements

Metabolomic analyses were conducted in 2020 randomly selected participants (ages 32–81 years) of the KORA F4 survey, a follow-up survey of KORA S4. Genotype information was available for 1814 of these participants. Fasting blood samples were collected in 2006–2008. Blood was drawn into serum gel tube in the morning between 8 and 10 am. The tube was gently inverted two times, followed by 30 minutes resting at room temperature to obtain complete coagulation, and finally centrifugation of blood was performed at 2750 g, 15°C for 10 minutes for serum collection. Serum was aliquoted and kept at 4°C for a maximum of 6 hours, after which it was frozen at −80°C until analyses. Liquid handling of serum samples (10 µl) was performed with Hamilton Star (Hamilton Bonaduz AG, Bonaduz, Switzerland) robot and prepared for quantification with AbsoluteIDQ kit (BIOCRATES Life Sciences AG, Innsbruck, Austria). Sample analyses were done on API4000 Q TRAP LC/MS/MS System (Applied Biosystems, Darmstadt, Germany) equipped with Schimadzu Prominence LC20AD pump and SIL-20AC auto sampler. The complete analytical process (e.g. the targeted metabolite concentration) was performed using the Met*IQ* software package, which is an integral part of the Absolute*IDQ* Kit. A total of 163 metabolites were measured. The metabolomics dataset contains 14 amino acids, one sugar, 41 acylcarnitines, 15 sphingolipids, and 92 glycerophospholipids.

### Statistical Analysis

GWA scans were made using an additive genetic model on Z-scores, calculated by adjusting serum UA levels for age and sex using linear regression and standardizing residuals. In sex-specific association testing Z-scores were calculated in each stratum separately. Study-specific results of the most significant SNP at each locus are presented in Table S3. The results from all 14 GWA scans were combined into a fixed-effects meta-analysis with inverse variance weighting, using the METAL package (www.sph.umich.edu/csg/abecasis/metal). The individual studies were corrected for residual inflation of the test statistic using genomic control methods for genotyped and imputed SNPs combined [40]. For the overall meta-analysis, the inflation factor was 1.028, no further correction was applied. Quantile-quantile plots of the association results are shown in Figure S1, study-specific quantile-quantile plots are illustrated in Figure S2 and S3. Associations were considered genome-wide significant below p = 5×10^−8^, which corresponds to a Bonferroni correction for the estimated one million independent common variant tests in the human genome of European individuals [41]. We also tested whether the effect estimate resulting from the gender-specific fixed effect meta-analysis differed significantly between men and women by applying a t-test comparing effect and standard error estimates in men with the effect and standard error estimates in women. Genome-wide significant SNPs were tested for independent associations, by including all SNPs in a multiple regression model, and then performing inverse variance weighted meta-analysis, across all cohorts (except for Health 2000), of the coefficient for each SNP. The analysis of metabolites was performed using the same linear regression adjusted by sex and gender as in the genome-wide scan. To specify the dependency of uric acid on metabolite concentration, a univariate regression model without further transformation or adjustment was used. The multiple regression and metabolite analysis were performed using either posterior expected allele dosages, or on best-guess imputed genotypes, with the statistical analysis software R.

### Accession Numbers

The OMIM (http://www.ncbi.nlm.nih.gov/OMIM) accession numbers for genes mentioned in this article are *PDZK1* (603831), *GCKR* (600842), *SLC2A9* (606142), *ABCG2* (603756), *LRRC16A* (609593), *SLC17A1* (182308), *SLC22A11* (607097), and *SLC22A12* (607096). The HGNC (http://www.gene.ucl.ac.uk) accession number for *SLC16A9* is 23520.

## Supporting Information

Figure S1 Quantile-quantile plots of association results. Meta-analysis was performed using sample-size weighted Z-scores after cohort-specific genomic control. Shown are expected p-values plotted against observed p-values resulting from meta-analysis based on all subjects (1st row), only males (2nd row) and only females (3rd row) for all analysed SNPs (left column) and for all analysed SNPs excluding the *SLC2A9* region (GLUT9, right column).
(1.25 MB TIF)

Figure S2 Study-specific quantile-quantile plots. Shown are expected p-values plotted against observed p-values resulting from each single study before (black dots) and after (blue dots) genomic control correction. The study-specific λ-values were λ = 1.007 (BRIGHT), λ = 1.025 (CoLaus), λ = 1.013 (CROATIA), λ = 1.024 (Health 2000), λ = 1.006 (KORA F3), λ = 1.016 (KORA S4), λ = 1.246 (MICROS), λ = 1.253 (NSPHS), λ = 1.182 (ORCADES), λ = 1.022 (PROCARDIS), λ = 1.090 (SardiNIA), λ = 1.031 (SHIP), λ = 1.022 (SSAGA) and λ = 1.122 (TwinsUK). For the overall meta-analysis, the inflation factor was 1.028.
(1.95 MB TIF)

Figure S3 Study-specific quantile-quantile plots excluding GLUT9. Shown are expected p-values plotted against observed p-values resulting from each single study before (black dots) and after (blue dots) genomic control correction, excluding SNPs in the *SLC2A9* (GLUT9) region on chromosome 4 (positions 9194245 to 10270832).
(1.91 MB TIF)

Table S1 Study sample characteristics. Characteristics are shown by study for British Genetics of Hypertension (BRIGHT), Cohorte Lausannoise (CoLaus), Vis island isolate study (CROATIA), Health 2000 cohort (Health 2000), two surveys of the Cooperative Health Research in the Region of Augsburg (KORA F3, KORA S4), Orkney Complex Disease Study (ORCADES), Precocious Coronary Artery Disease (PROCARDIS), Northern Swedish Population Health Study (NSPHS), SardiNIA Study of Aging (SardiNIA), Study of Health in Pomerania (SHIP), Semi-Structured Assessment for Genetics of Alcoholism (SSAGA), Microisolates in South Tyrol (MICROS) and UK Adult Twin Register (TwinsUK). Age is given as mean and range in brackets. Uric acid concentrations (UA) are given as mean and appropriate standard deviation (SD). NA indicates not applicable.
(0.06 MB DOC)

Table S2 Genotyping, imputation and analysis procedures by study. Shown are the genotyping platforms, quality control (QC) filters applied before imputation, imputation software, number of SNPs and genotype-phenotype association software.
(0.07 MB DOC)

Table S3 Study-specific results. Shown are study-specific results of the most significant SNP at each locus.
(0.23 MB DOC)

Table S4 Full list of genome-wide significant SNPs. Shown is a full list of SNPs that exceeded the threshold of genome-wide significance (p<5×10−8). Position is given for NCBI Build 36. Effect estimates result from additive linear regression on Z-scores of uric acid concentrations. P-values have been calculated using weighting by the inverse variance. The effect allele is the allele to which the beta (effect) estimate refers.
(2.01 MB DOC)

Table S5 Multiple regression analysis. Genome-wide significant SNPs were tested for independent associations, by including all nine SNPs in a multiple regression model, and then performing inverse variance weighted meta-analysis, across participating cohorts (except for Health2000), of the coefficient for each SNP.
(0.04 MB DOC)

Text S1 Study design. This section describes additional study specific characteristics.
(0.14 MB DOC)
