# Supplementary figures and images for: Meta-Analysis of 28,141 Individuals Identifies Common Variants within Five New Loci That Influence Uric Acid Concentrations
Source: PLoS Genet. 2009 Jun 5;5(6):e1000504. doi: 10.1371/journal.pgen.1000504 (PMC2683940; doi:10.1371/journal.pgen.1000504)

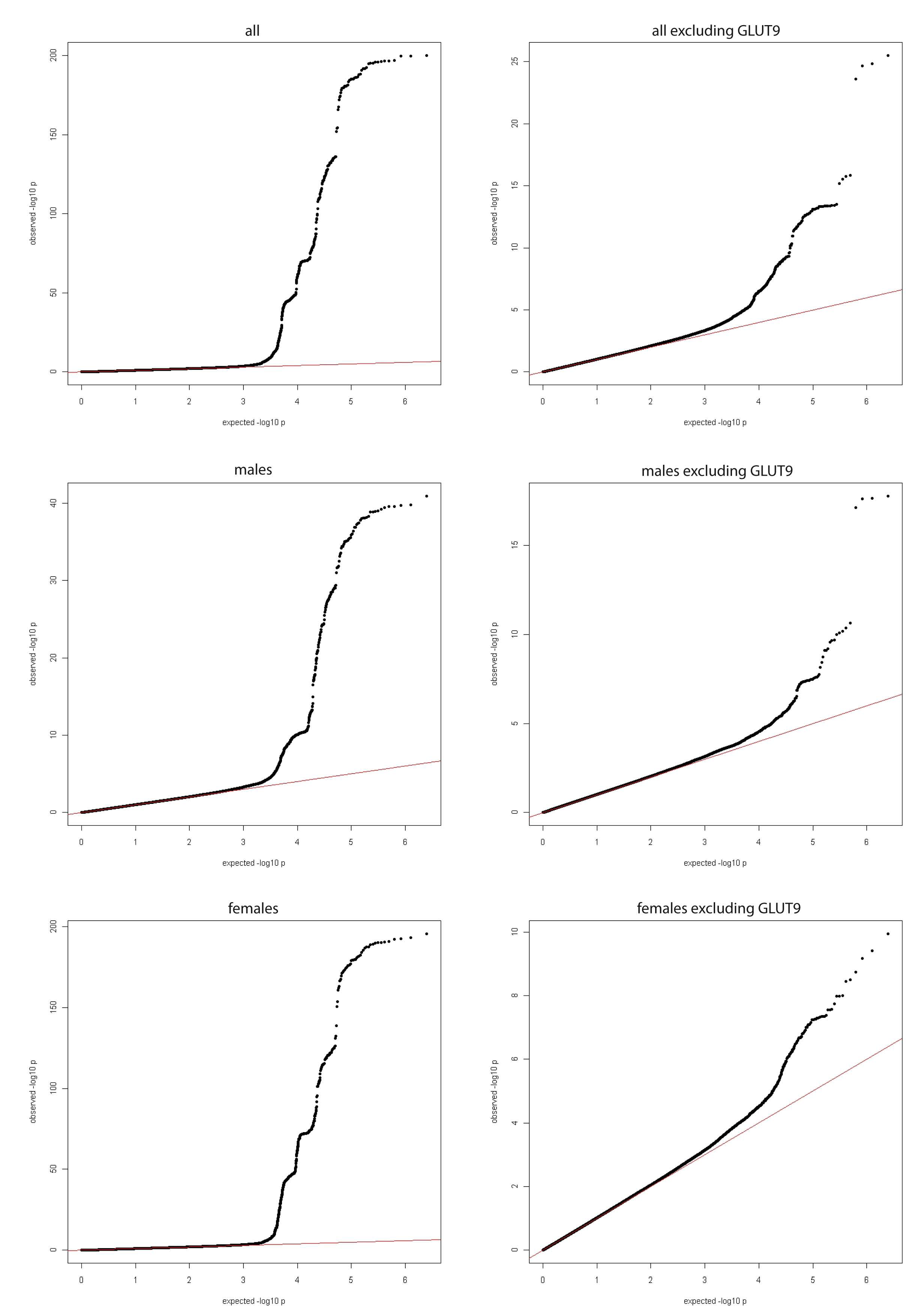

Supplement: Figure S1 — Quantile-quantile plots of association results. Meta-analysis was performed using sample-size weighted Z-scores after cohort-specific genomic control. Shown are expected p-values plotted against observed p-values resulting from meta-analysis based on all subjects (1st row), only males (2nd row) and only females (3rd row) for all analysed SNPs (left column) and for all analysed SNPs excluding the SLC2A9 region (GLUT9, right column). (1.25 MB TIF) [file pgen.1000504.s001.tif]

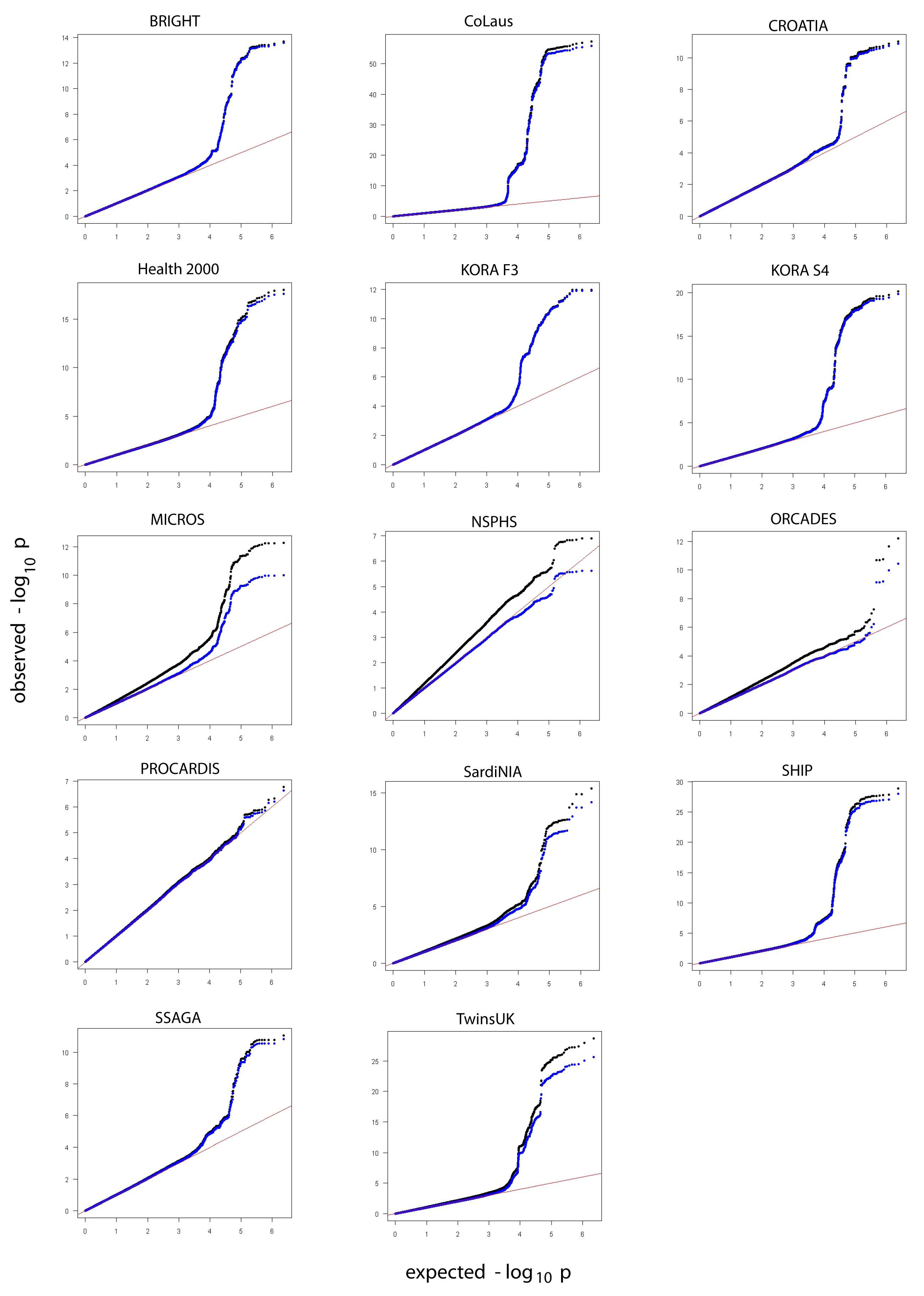

Supplement: Figure S2 — Study-specific quantile-quantile plots. Shown are expected p-values plotted against observed p-values resulting from each single study before (black dots) and after (blue dots) genomic control correction. The study-specific λ-values were λ = 1.007 (BRIGHT), λ = 1.025 (CoLaus), λ = 1.013 (CROATIA), λ = 1.024 (Health 2000), λ = 1.006 (KORA F3), λ = 1.016 (KORA S4), λ = 1.246 (MICROS), λ = 1.253 (NSPHS), λ = 1.182 (ORCADES), λ = 1.022 (PROCARDIS), λ = 1.090 (SardiNIA), λ = 1.031 (SHIP), λ = 1.022 (SSAGA) and λ = 1.122 (TwinsUK). For the overall meta-analysis, the inflation factor was 1.028. (1.95 MB TIF) [file pgen.1000504.s002.tif]

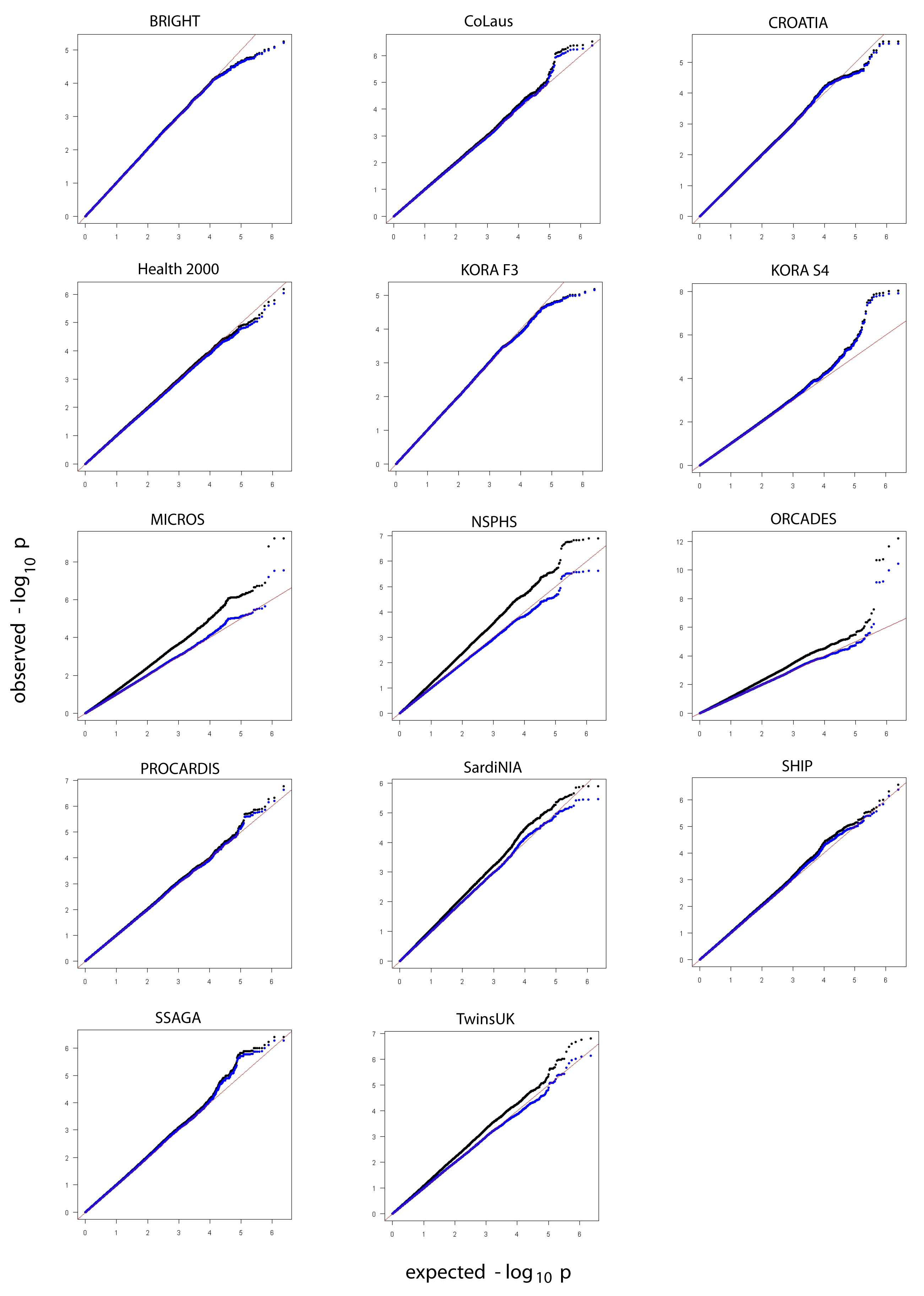

Supplement: Figure S3 — Study-specific quantile-quantile plots excluding GLUT9. Shown are expected p-values plotted against observed p-values resulting from each single study before (black dots) and after (blue dots) genomic control correction, excluding SNPs in the SLC2A9 (GLUT9) region on chromosome 4 (positions 9194245 to 10270832). (1.91 MB TIF) [file pgen.1000504.s003.tif]
