# Supplementary material for: Meta-Analysis of 28,141 Individuals Identifies Common Variants within Five New Loci That Influence Uric Acid Concentrations
Source: PLoS Genet. 2009 Jun 5;5(6):e1000504. doi: 10.1371/journal.pgen.1000504 (PMC2683940; doi:10.1371/journal.pgen.1000504)
Supplement: Table S1 — Study sample characteristics. Characteristics are shown by study for British Genetics of Hypertension (BRIGHT), Cohorte Lausannoise (CoLaus), Vis island isolate study (CROATIA), Health 2000 cohort (Health 2000), two surveys of the Cooperative Health Research in the Region of Augsburg (KORA F3, KORA S4), Orkney Complex Disease Study (ORCADES), Precocious Coronary Artery Disease (PROCARDIS), Northern Swedish Population Health Study (NSPHS), SardiNIA Study of Aging (SardiNIA), Study of Health in Pomerania (SHIP), Semi-Structured Assessment for Genetics of Alcoholism (SSAGA), Microisolates in South Tyrol (MICROS) and UK Adult Twin Register (TwinsUK). Age is given as mean and range in brackets. Uric acid concentrations (UA) are given as mean and appropriate standard deviation (SD). NA indicates not applicable. (0.06 MB DOC) [file pgen.1000504.s004.doc]

| **Study acronym** | **Sample size** | | | **Age in years (range)** | | | **UA in mg/dl (SD)** | | |
| --- | --- | --- | --- | --- | --- | --- | --- | --- | --- |
|  | total | males | females | total | females | males | total | females | males |
| BRIGHT | 1743 | 690 | 1053 | 56.87 (21-85) | 57.31 (21-85) | 56.20 (23-84) | 5.39 (1.44) | 4.92 (1.26) | 6.11 (1.39) |
| CoLaus | 5411 | 2546 | 2865 | 53.43 (35-75) | 53.88 (35-75) | 52.93 (35-75) | 5.27 (1.42) | 4.56 (1.13) | 6.07 (1.27) |
| CROATIA | 774 | 320 | 454 | 56.55 (18-93) | 56.97 (18-93) | 55.95 (18-88) | 5.27 (1.53) | 4.66 (1.31) | 6.14 (1.38) |
| Health 2000 | 2212 | 1088 | 1124 | 50.44 (30-75) | 51.87 (30-75) | 48.96 (30-75) | 3.06 (0.77) | 2.69 (0.66) | 3.44 (0.68) |
| KORA F3 | 1644 | 813 | 831 | 62.50 (35-79) | 62.09 (35-79) | 62.96 (35-79) | 5.21 (1.36) | 4.60 (1.20) | 5.82 (1.24) |
| KORA S4 | 1814 | 884 | 930 | 56.43 (45–69) | 56.03 (45-69) | 56.85 (45-69) | 5.40 (1.44) | 4.63 (1.09) | 6.19 (1.32) |
| MICROS | 1086 | 471 | 615 | 45.26 (18-88) | 45.38 (18-84) | 45.09 (18-88) | 5.33 (1.53) | 4.65 (1.19) | 6.21 (1.45) |
| NSPHS | 655 | 309 | 346 | 46.98 (14-91) | 46.47 (14-91) | 47.56 (15-87) | 5.38 (1.42) | 4.74 (1.25) | 6.08 (1.27) |
| ORCADES | 715 | 331 | 384 | 53.59 (17-98) | 53.01 (18-98) | 54.27 (17-94) | 5.29 (1.34) | 4.67 (1.13) | 6.02 (1.2) |
| PROCARDIS | 1203 | 974 | 229 | 61.05 (37-82) | 62.61 (44-82) | 60.72 (37-82) | 6.17 (1.44) | 5.48 (1.36) | 6.33 (1.42) |
| SardiNIA | 4305 | 1886 | 2419 | 43.58 (14-101) | 43.19 (14-101) | 44.08 (14-94) | 4.32 (1.48) | 3.62 (1.15) | 5.24 (1.36) |
| SHIP | 4087 | 2016 | 2071 | 49.79 (21-80) | 48.79 (20-81) | 50.81 (20-81) | 4.39 (1.43) | 4.19 (1.15) | 5.70 (1.29) |
| SSAGA | 379 | 0 | 379 | 46.72 (30-83) | 46.72 (30-83) | NA | 4.82 (1.25) | 4.82 (1.25) | NA |
| TwinsUK | 2113 | 0 | 2113 | 47.24 (18-79) | 47.24 (18-79) | NA | 4.49 (1.07) | 4.49 (1.07) | NA |
