# Supplementary material for: Meta-Analysis of 28,141 Individuals Identifies Common Variants within Five New Loci That Influence Uric Acid Concentrations
Source: PLoS Genet. 2009 Jun 5;5(6):e1000504. doi: 10.1371/journal.pgen.1000504 (PMC2683940; doi:10.1371/journal.pgen.1000504)
Supplement: Table S2 — Genotyping, imputation and analysis procedures by study. Shown are the genotyping platforms, quality control (QC) filters applied before imputation, imputation software, number of SNPs and genotype-phenotype association software. (0.07 MB DOC) [file pgen.1000504.s005.doc]

| **Study acronym** | **Genotyping** | | **QC filters before Imputation** | | | | **Imputation** | | | **SNPs for analysis** | | | **Statistical software** |
| --- | --- | --- | --- | --- | --- | --- | --- | --- | --- | --- | --- | --- | --- |
|  | **Platform** | **Calling algorithm** | **Individual Callrate** | **SNP Callrate** | **HWE** | **MAF** | **SNPs for imputation** | **NCBI Build** | **Method** | **Total** | **Females** | **Males** |  |
|  |  |  |  |  |  |  |  |  |  |  |  |  |  |
| BRIGHT | Affymetrix 500K | CHIAMO | < 97% | <95% MAF>0.05 <99% MAF>0.05 | < 5.7E-7 | NA | 490032 | 35 | IMPUTE | 2361716 | 2360458 | 2359934 | SNPTEST |
| CoLaus | Affymetrix 500K | BRLMM | <95% | < 70% | < 10-7 | <0 | 390631 | 35 | IMPUTE v0.2 | 2431919 | 2431215 | 2431841 | custom C++ |
| CROATIA | Illumina HumanHap300 (v1) | Bead Studio | < 97% | < 98% | < 10-6 | <1% | 305068 | 36 | MACH v1.0.15 | 2392574 | 2391744 | 2388768 | ProABEL |
| Health 2000 | Illumina 610K | GenCall | <95% | < 95% | < 0.0001 | <0.02 | 598203 | 35 | MACH 1.0.10 | 2449205 | 2449629 | 2449606 | PLINK v1.04 |
| KORA F3 | Affymetrix 500K | BRLMM | < 93% each Chip | < 90% | NA | NA | 490032 | 35 | MACH v1.0.9 | 2374873 | 2374873 | 2374873 | MACH2QTL v1.0.4 |
| KORA F4 | Affymetrix 6.0 | Birdseed2 | < 93% each Chip | < 90% | NA | NA | 909622 | 36 | IMPUTE v0.4.2 | 2488915 | 2488712 | 2488079 | SNPTEST v1.1.5 |
| ORCADES | Illumina HumanHap300 (v2) | Bead Studio | <98% | < 98% | < 10-6 | <1% | 306207 | 36 | MACH v1.0.15 | 2402399 | 2400913 | 2401026 | ProABEL |
| PROCARDIS | Illumina 1M | Bead Studio | <95% | < 95% | < 10-3 | NA | 882598 | 36 | IMPUTE v0.3.2 | 2478059 | 2477051 | 2477940 | SNPTEST |
| NSPHS | Illumina Infinium HumanHap300v2 | Bead Studio | 0.97 | 0.9 | 0.000001 | 0.01 | 315315 | 36 | MACH v1.0 | 2364271 | 2364439 | 2363905 | ProbABEL |
| SardiNIA | Affymetrix 500K | BRLMM | < 95% each Chip | < 90% | < 1x10^-6^ | <5% | 356 359 | 35 | MACH v1.0.9 | 2251300 | 2251833 | 2251859 | Merlin --fastassoc |
| SHIP | Affymetrix 6.0 | Birdseed2 | < 86% each Chip | NA | NA | NA | 869224 | 36 | IMPUTE v0.5.0 | 2493963 | 2493107 | 2493160 | SNPTEST v1.1.5 |
| SSAGA | Illumina HumanHap300 | Beadstudio 2.0 | NA | NA | NA | NA | 312431 | 35 | MACH v1.0.16 | NA | 2416796 | NA | PLINK v1.04 |
| MICROS | Illumina HumanHap300 (v2) | Bead Studio | < 98% | < 98% | < 10-6 | <1% | 306207 | 36 | MACH v1.0.15 | 2403686 | 2402814 | 2400511 | ProABEL |
| TwinsUK | Illumina 317K | Illumina protocol | < 97% | > 0.95 | < 1x10^-4^ | 0.01 | 279801 | 36 | IMPUTE v0.4.2 | NA | 2286606 | NA | GenABEL |
