# Supplementary material for: Meta-Analysis of 28,141 Individuals Identifies Common Variants within Five New Loci That Influence Uric Acid Concentrations
Source: PLoS Genet. 2009 Jun 5;5(6):e1000504. doi: 10.1371/journal.pgen.1000504 (PMC2683940; doi:10.1371/journal.pgen.1000504)
Supplement: Table S3 — Study-specific results. Shown are study-specific results of the most significant SNP at each locus. (0.23 MB DOC) [file pgen.1000504.s006.doc]

|  |  | **BRIGHT** | **CoLaus** | **CROATIA** | **Health 2000** | **KORA F3** | **KORA S4** | **ORCADES** | **PROCARDIS** | **NSPHS** | **SardiNIA** | **SHIP** | **SSAGA** | **TwinsUK** | **MICROS** |
| --- | --- | --- | --- | --- | --- | --- | --- | --- | --- | --- | --- | --- | --- | --- | --- |
| **rs12129861** | n | 1743 | 5411 | 774 | 2138 | 1644 | 1500 | 715 | 1201 | 656 | 4305 | 3574 | 379 | 501 | 1086 |
|  | beta | 0.114 | -0.078 | -0.064 | 0.061 | 0.052 | 0.110 | 0.039 | 0.129 | -0.014 | 0.021 | 0.041 | 0.032 | -0.057 | -0.020 |
|  | sebeta | 0.042 | 0.024 | 0.061 | 0.032 | 0.047 | 0.036 | 0.066 | 0.040 | 0.057 | 0.033 | 0.022 | 0.077 | 0.066 | 0.054 |
|  | p | 6.56E-03 | 1.37E-03 | 2.97E-01 | 5.80E-02 | 2.66E-01 | 2.01E-03 | 5.48E-01 | 1.42E-03 | 8.05E-01 | 5.22E-01 | 6.85E-02 | 6.75E-01 | 3.85E-01 | 7.05E-01 |
|  | effallele | G | A | A | G | G | G | A | G | A | G | G | G | A | A |
|  | effallelefreq | 52.21% | 45.48% | 47.40% | 44.22% | 49.85% | 52.93% | 45.76% | 54.76% | 43.22% | 59.10% | 50.91% | 44.86% | 16.37% | 50.34% |
|  | impute | I | I | I | I | I | I | I | G | I | I | I | I | I | I |
| **rs780094** | n | 1743 | 5411 | 769 | 2138 | 1644 | 1768 | 714 | 1202 | 652 |  | 4085 | 374 | 2101 | 1085 |
|  | beta | 0.097 | 0.063 | -0.056 | 0.062 | -0.021 | 0.077 | -0.131 | 0.087 | 0.131 |  | -0.003 | -0.083 | 0.058 | -0.037 |
|  | sebeta | 0.036 | 0.019 | 0.051 | 0.032 | 0.036 | 0.034 | 0.053 | 0.043 | 0.054 |  | 0.022 | 0.078 | 0.018 | 0.044 |
|  | p | 6.31E-03 | 8.65E-04 | 2.68E-01 | 5.03E-02 | 5.56E-01 | 2.43E-02 | 1.38E-02 | 4.37E-02 | 1.56E-02 |  | 8.76E-01 | 2.90E-01 | 1.39E-03 | 4.04E-01 |
|  | effallele | T | T | C | T | T | T | C | T | A |  | T | A | T | C |
|  | effallelefreq | 39.83% | 45.66% | 55.70% | 36.30% | 43.10% | 41.32% | 65.37% | 39.30% | 41.64% |  | 38.64% | 38.91% | 38.55% | 57.62% |
|  | impute | G | G | G | G | G | G | G | G | G |  | G | G | G | G |
| **rs734553** | n | 1743 | 5411 | 774 | 2138 | 1644 | 1737 | 715 | 1201 | 656 | 4305 | 3953 | 379 | 2075 | 1086 |
|  | beta | 0.310 | -0.353 | 0.358 | -0.309 | -0.290 | 0.387 | 0.310 | 0.170 | 0.251 | -0.064 | 0.299 | -0.549 | -0.283 | 0.335 |
|  | sebeta | 0.044 | 0.022 | 0.054 | 0.038 | 0.043 | 0.044 | 0.068 | 0.050 | 0.060 | 0.031 | 0.026 | 0.080 | 0.026 | 0.049 |
|  | p | 1.13E-12 | 4.92E-56 | 4.37E-11 | 7.30E-01 | 1.40E-11 | 5.54E-19 | 6.09E-06 | 5.90E-04 | 3.33E-05 | 3.90E-02 | 1.23E-29 | 2.27E-11 | 6.02E-28 | 8.83E-12 |
|  | effallele | T | G | T | G | G | T | T | T | T | C | T | G | G | T |
|  | effallelefreq | 75.50% | 24.63% | 70.33% | 18.17% | 25.64% | 77.72% | 82.17% | 76.91% | 83.85% | 54.30% | 77.70% | 21.91% | 23.25% | 73.69% |
|  | impute | I | I | I | I | I | I | I | G | I | G | I | I | I | I |
| **rs2231142** | n | 1743 | 5411 | 774 | 2138 | 1644 | 1763 | 715 | 1200 | 655 | 4305 | 4005 | 376 | 2112 | 1086 |
|  | beta | 0.193 | 0.201 | -0.110 | 0.162 | 0.095 | 0.299 | -0.312 | 0.199 | 0.219 | 0.291 | 0.124 | 0.191 | 0.129 | -0.157 |
|  | sebeta | 0.066 | 0.040 | 0.100 | 0.058 | 0.064 | 0.054 | 0.110 | 0.066 | 0.075 | 0.036 | 0.036 | 0.119 | 0.043 | 0.064 |
|  | p | 3.27E-03 | 4.25E-07 | 2.71E-01 | 5.52E-03 | 1.38E-01 | 2.61E-08 | 4.69E-03 | 2.39E-03 | 3.34E-03 | 4.26E-16 | 5.55E-04 | 1.11E-01 | 2.64E-03 | 1.35E-02 |
|  | effallele | T | T | G | T | T | T | G | T | A | T | T | A | T | G |
|  | effallelefreq | 13.35% | 11.65% | 92.89% | 7.37% | 10.52% | 10.98% | 93.64% | 11.32% | 16.49% | 76.30% | 9.95% | 12.01% | 10.30% | 86.10% |
|  | impute | I | I | G | G | I | I | G | G | G | I | I | G | G | G |
| **rs742132** | n | 1743 | 5411 | 774 | 2138 | 1644 | 1779 | 715 | 1203 | 656 |  | 4022 | 379 | 2068 | 1086 |
|  | beta | -0.096 | -0.053 | -0.010 | -0.036 | 0.076 | -0.024 | -0.057 | -0.104 | 0.011 |  | -0.060 | -0.064 | -0.088 | -0.050 |
|  | sebeta | 0.037 | 0.021 | 0.055 | 0.035 | 0.038 | 0.035 | 0.058 | 0.045 | 0.054 |  | 0.024 | 0.079 | 0.023 | 0.044 |
|  | p | 1.05E-02 | 1.05E-02 | 8.57E-01 | 3.06E-01 | 4.34E-02 | 4.90E-01 | 3.27E-01 | 2.12E-02 | 8.43E-01 |  | 1.23E-02 | 4.16E-01 | 1.02E-04 | 2.56E-01 |
|  | effallele | G | G | G | G | A | G | G | G | G |  | G | G | G | G |
|  | effallelefreq | 28.99% | 31.26% | 31.93% | 25.65% | 31.87% | 32.27% | 30.49% | 29.54% | 24.62% |  | 30.77% | 30.61% | 29.09% | 40.80% |
|  | impute | G | G | I | I | G | G | I | I | I |  | G | I | I | I |
| **rs1183201** | n | 1743 | 5411 | 774 | 2138 | 1644 | 1757 | 715 | 1203 | 656 | 4305 | 3987 | 379 | 2110 | 1086 |
|  | beta | 0.075 | 0.056 | 0.092 | -0.121 | -0.024 | 0.098 | 0.094 | 0.055 | 0.061 | -0.085 | 0.047 | -0.189 | -0.031 | 0.117 |
|  | sebeta | 0.034 | 0.019 | 0.053 | 0.031 | 0.035 | 0.034 | 0.052 | 0.041 | 0.046 | 0.030 | 0.022 | 0.073 | 0.016 | 0.044 |
|  | p | 2.77E-02 | 3.76E-03 | 8.17E-02 | 1.18E-04 | 4.96E-01 | 3.39E-03 | 7.25E-02 | 1.76E-01 | 1.83E-01 | 5.40E-03 | 3.10E-02 | 1.04E-02 | 4.42E-02 | 7.59E-03 |
|  | effallele | T | T | T | A | A | T | T | T | T | A | T | A | A | T |
|  | effallelefreq | 54.03% | 49.83% | 59.00% | 38.47% | 49.30% | 50.20% | 51.67% | 53.78% | 51.84% | 52.60% | 51.28% | 47.59% | 45.95% | 49.55% |
|  | impute | I | I | I | I | I | I | I | I | I | I | I | I | I | I |
| **rs12356193** | n | 1743 | 5411 | 774 | 2138 | 1644 | 1734 | 715 | 1203 | 656 | 4305 | 4010 | 379 | 2066 | 1086 |
|  | beta | -0.097 | -0.080 | 0.123 | -0.127 | -0.054 | -0.135 | 0.115 | -0.053 | 0.060 | 0.101 | -0.090 | 0.105 | -0.043 | 0.024 |
|  | sebeta | 0.055 | 0.033 | 0.067 | 0.052 | 0.063 | 0.047 | 0.061 | 0.054 | 0.106 | 0.031 | 0.031 | 0.091 | 0.033 | 0.056 |
|  | p | 7.78E-02 | 1.49E-02 | 6.72E-02 | 1.45E-02 | 3.92E-01 | 4.32E-03 | 5.78E-02 | 3.23E-01 | 5.73E-01 | 1.37E-03 | 3.78E-03 | 2.47E-01 | 1.88E-01 | 6.62E-01 |
|  | effallele | G | G | A | G | G | G | A | G | A | A | G | G | G | A |
|  | effallelefreq | 19.47% | 22.05% | 82.30% | 9.75% | 18.19% | 15.95% | 74.83% | 16.47% | 94.43% | 57.10% | 14.61% | 17.82% | 17.06% | 81.00% |
|  | impute | I | I | I | G | I | I | I | G | I | I | I | I | I | I |
| **rs17300741** | n | 1743 | 5411 | 774 | 2138 | 1644 | 1712 | 715 | 1198 | 656 | 4305 | 3886 | 379 | 2080 | 1086 |
|  | beta | 0.020 | -0.068 | -0.085 | -0.050 | 0.054 | -0.066 | -0.079 | -0.070 | -0.032 | -0.109 | -0.100 | 0.112 | 0.044 | -0.043 |
|  | sebeta | 0.037 | 0.020 | 0.051 | 0.030 | 0.036 | 0.034 | 0.054 | 0.041 | 0.048 | 0.037 | 0.022 | 0.076 | 0.016 | 0.042 |
|  | p | 5.85E-01 | 5.02E-04 | 9.77E-02 | 9.90E-02 | 1.38E-01 | 5.26E-02 | 1.43E-01 | 8.99E-02 | 5.09E-01 | 2.87E-03 | 5.04E-06 | 1.40E-01 | 5.08E-03 | 3.05E-01 |
|  | effallele | G | G | G | G | A | G | G | G | G | T | G | A | A | G |
|  | effallelefreq | 54.00% | 49.67% | 45.62% | 43.50% | 48.57% | 52.13% | 54.24% | 52.31% | 34.70% | 77.70% | 49.65% | 46.33% | 46.35% | 53.69% |
|  | impute | I | I | I | I | I | I | I | G | I | I | I | I | I | I |
| **rs505802** | n | 1743 | 5411 | 774 | 2138 | 1644 | 1766 | 715 | 1203 | 655 | 4305 | 4038 | 376 | 2113 | 1086 |
|  | beta | -0.043 | 0.070 | -0.047 | 0.026 | 0.032 | -0.042 | -0.071 | -0.089 | 0.101 | -0.027 | -0.079 | 0.155 | 0.012 | -0.028 |
|  | sebeta | 0.037 | 0.021 | 0.054 | 0.031 | 0.039 | 0.037 | 0.055 | 0.045 | 0.056 | 0.033 | 0.023 | 0.078 | 0.022 | 0.049 |
|  | p | 2.40E-01 | 7.63E-04 | 3.82E-01 | 4.07E-01 | 4.08E-01 | 2.54E-01 | 1.96E-01 | 4.53E-02 | 7.39E-02 | 4.19E-01 | 6.44E-04 | 4.87E-02 | 5.92E-01 | 5.70E-01 |
|  | effallele | T | C | T | C | C | T | T | T | G | A | T | G | C | T |
|  | effallelefreq | 70.90% | 30.23% | 66.42% | 39.64% | 30.96% | 69.85% | 69.49% | 69.96% | 48.63% | 70.40% | 69.40% | 30.09% | 29.70% | 71.33% |
|  | impute | I | I | G | G | I | I | G | G | G | G | I | G | G | G |
