# Supplementary material for: Meta-Analysis of 28,141 Individuals Identifies Common Variants within Five New Loci That Influence Uric Acid Concentrations
Source: PLoS Genet. 2009 Jun 5;5(6):e1000504. doi: 10.1371/journal.pgen.1000504 (PMC2683940; doi:10.1371/journal.pgen.1000504)
Supplement: Table S4 — Full list of genome-wide significant SNPs. Shown is a full list of SNPs that exceeded the threshold of genome-wide significance (p<5×10^−8^). Position is given for NCBI Build 36. Effect estimates result from additive linear regression on Z-scores of uric acid concentrations. P-values have been calculated using weighting by the inverse variance. The effect allele is the allele to which the beta (effect) estimate refers. (2.01 MB DOC) [file pgen.1000504.s007.doc]

|  |  |  | **Allele** | |  |  |  |  |
| --- | --- | --- | --- | --- | --- | --- | --- | --- |
| **db SNP** | **Chromosome** | **Position** | **Effect** | **Other** | **N** | **Beta** | **SE (Beta)** | **p-value** |
| rs10910845 | 1 | 1.44E+08 | A | C | 26613 | 0.0535 | 0.0095 | 1.57E-08 |
| rs1967017 | 1 | 1.44E+08 | T | C | 26554 | 0.0541 | 0.0093 | 5.00E-09 |
| rs1471633 | 1 | 1.44E+08 | A | C | 26514 | 0.0546 | 0.0093 | 3.77E-09 |
| rs12129861 | 1 | 1.44E+08 | A | G | 25627 | -0.0623 | 0.0105 | 2.68E-09 |
| rs9728619 | 1 | 1.44E+08 | T | C | 26392 | 0.0499 | 0.0091 | 3.99E-08 |
| rs1260326 | 2 | 27584444 | T | C | 27765 | 0.0511 | 0.0085 | 1.82E-09 |
| rs780094 | 2 | 27594741 | T | C | 27991 | 0.0515 | 0.0085 | 1.40E-09 |
| rs780093 | 2 | 27596107 | T | C | 27994 | 0.0502 | 0.0085 | 2.94E-09 |
| rs1260333 | 2 | 27602128 | A | G | 27562 | 0.0459 | 0.0083 | 3.19E-08 |
| rs13138961 | 4 | 9194245 | T | G | 26530 | 0.0645 | 0.0112 | 7.30E-09 |
| rs12512447 | 4 | 9195411 | A | G | 26774 | 0.0722 | 0.0109 | 4.08E-11 |
| rs13103207 | 4 | 9212770 | T | C | 22551 | 0.0784 | 0.0135 | 6.28E-09 |
| rs6821253 | 4 | 9299829 | T | G | 27194 | -0.0627 | 0.0107 | 4.75E-09 |
| rs13103452 | 4 | 9303760 | T | C | 27249 | -0.0625 | 0.0107 | 4.61E-09 |
| rs13136075 | 4 | 9324066 | A | G | 28004 | 0.0509 | 0.0089 | 1.16E-08 |
| rs9684176 | 4 | 9325484 | T | G | 28000 | -0.0515 | 0.0089 | 8.28E-09 |
| rs12501880 | 4 | 9327073 | T | C | 27958 | 0.0513 | 0.0089 | 8.13E-09 |
| rs11730320 | 4 | 9328999 | T | C | 24700 | 0.0610 | 0.0106 | 8.68E-09 |
| rs13121465 | 4 | 9331373 | T | G | 26678 | -0.0702 | 0.0097 | 4.02E-13 |
| rs13128435 | 4 | 9332770 | A | G | 26866 | -0.0684 | 0.0094 | 4.11E-13 |
| rs13127001 | 4 | 9340313 | A | G | 26860 | 0.0660 | 0.0095 | 2.81E-12 |
| rs6448981 | 4 | 9349423 | C | G | 26777 | 0.0547 | 0.0098 | 2.23E-08 |
| rs13140817 | 4 | 9372073 | A | G | 27574 | 0.0671 | 0.0090 | 8.87E-14 |
| rs7655090 | 4 | 9374973 | A | G | 27561 | 0.0538 | 0.0093 | 8.37E-09 |
| rs11731100 | 4 | 9376278 | A | T | 27647 | -0.0661 | 0.0089 | 1.42E-13 |
| rs11732272 | 4 | 9377356 | A | G | 27755 | 0.0751 | 0.0130 | 6.67E-09 |
| rs10939507 | 4 | 9377921 | T | C | 27976 | -0.1162 | 0.0205 | 1.46E-08 |
| rs10033951 | 4 | 9388678 | T | C | 27706 | -0.0523 | 0.0093 | 1.68E-08 |
| rs2867383 | 4 | 9397033 | A | G | 27849 | -0.0504 | 0.0091 | 3.04E-08 |
| rs7685396 | 4 | 9403822 | A | C | 27583 | 0.0659 | 0.0088 | 8.43E-14 |
| rs13106539 | 4 | 9406801 | T | C | 27860 | 0.0654 | 0.0087 | 6.77E-14 |
| rs2280207 | 4 | 9408874 | T | C | 27855 | -0.0650 | 0.0087 | 8.33E-14 |
| rs1519097 | 4 | 9411951 | A | G | 27871 | 0.0533 | 0.0092 | 5.98E-09 |
| rs1519096 | 4 | 9414970 | T | C | 27868 | 0.0618 | 0.0113 | 4.97E-08 |
| rs2280333 | 4 | 9420231 | A | G | 27753 | 0.0690 | 0.0088 | 4.78E-15 |
| rs1401438 | 4 | 9423554 | A | G | 27868 | -0.0645 | 0.0116 | 2.69E-08 |
| rs13148356 | 4 | 9426168 | A | G | 27839 | 0.0665 | 0.0088 | 4.75E-14 |
| rs13141706 | 4 | 9427443 | T | C | 27731 | -0.0721 | 0.0090 | 1.13E-15 |
| rs6855095 | 4 | 9429075 | A | G | 27412 | -0.0725 | 0.0090 | 9.85E-16 |
| rs1107912 | 4 | 9429277 | A | G | 27399 | -0.0751 | 0.0089 | 4.40E-17 |
| rs4697892 | 4 | 9432935 | A | C | 27320 | -0.1073 | 0.0108 | 2.03E-23 |
| rs1980220 | 4 | 9433734 | A | G | 27945 | -0.1096 | 0.0105 | 2.71E-25 |
| rs6822889 | 4 | 9435855 | T | C | 27963 | 0.0813 | 0.0088 | 2.28E-20 |
| rs4621429 | 4 | 9435968 | A | G | 28046 | 0.1113 | 0.0105 | 2.42E-26 |
| rs4697895 | 4 | 9437582 | T | C | 27932 | 0.1122 | 0.0105 | 1.59E-26 |
| rs1401440 | 4 | 9441610 | T | C | 28037 | 0.0808 | 0.0087 | 2.12E-20 |
| rs1464258 | 4 | 9443205 | T | G | 27964 | 0.0814 | 0.0088 | 1.41E-20 |
| rs16889260 | 4 | 9443994 | T | C | 27798 | -0.1210 | 0.0113 | 5.54E-27 |
| rs16889264 | 4 | 9444097 | T | C | 25202 | 0.1590 | 0.0289 | 3.95E-08 |
| rs12505312 | 4 | 9446188 | C | G | 28037 | 0.1117 | 0.0105 | 1.25E-26 |
| rs10939552 | 4 | 9447646 | A | C | 28037 | -0.1118 | 0.0105 | 1.14E-26 |
| rs1914874 | 4 | 9448588 | A | C | 28032 | -0.0800 | 0.0090 | 6.85E-19 |
| rs10031303 | 4 | 9450788 | A | T | 28038 | -0.0811 | 0.0087 | 1.37E-20 |
| rs950310 | 4 | 9451948 | A | T | 27979 | -0.1230 | 0.0112 | 2.79E-28 |
| rs10939558 | 4 | 9453260 | T | C | 27508 | -0.0835 | 0.0089 | 5.41E-21 |
| rs7683831 | 4 | 9454177 | T | C | 27956 | 0.0796 | 0.0090 | 9.35E-19 |
| rs13141635 | 4 | 9455083 | T | C | 27960 | -0.0810 | 0.0087 | 1.38E-20 |
| rs1976792 | 4 | 9455407 | A | G | 27983 | -0.1126 | 0.0118 | 1.92E-21 |
| rs13119059 | 4 | 9456958 | C | G | 27961 | 0.0804 | 0.0087 | 2.56E-20 |
| rs11721988 | 4 | 9457997 | A | G | 28017 | 0.1147 | 0.0105 | 6.51E-28 |
| rs2176644 | 4 | 9458626 | T | C | 28015 | -0.1149 | 0.0105 | 5.42E-28 |
| rs6812007 | 4 | 9459318 | A | C | 27919 | -0.0788 | 0.0090 | 1.76E-18 |
| [rs4697898](http://www.ncbi.nlm.nih.gov/SNP/snp_ref.cgi?rs=4697898) | 4 | 9460925 | T | C | 13482 | -0.1043 | 0.0162 | 1.12E-10 |
| [rs4697899](http://www.ncbi.nlm.nih.gov/SNP/snp_ref.cgi?rs=4697899) | 4 | 9460973 | A | C | 13482 | -0.1008 | 0.0161 | 3.66E-10 |
| rs6831796 | 4 | 9461996 | C | G | 27741 | 0.1156 | 0.0104 | 6.73E-29 |
| rs6826806 | 4 | 9465567 | A | G | 28000 | 0.1110 | 0.0104 | 9.21E-27 |
| [rs4507358](http://www.ncbi.nlm.nih.gov/SNP/snp_ref.cgi?rs=4507358) | 4 | 9467054 | T | G | 13482 | -0.1028 | 0.0159 | 9.81E-11 |
| rs4697900 | 4 | 9469074 | A | G | 27312 | 0.0703 | 0.0090 | 4.53E-15 |
| rs9684729 | 4 | 9471134 | T | C | 27994 | -0.0782 | 0.0090 | 2.80E-18 |
| rs4697903 | 4 | 9474524 | A | G | 28004 | -0.1099 | 0.0103 | 1.90E-26 |
| rs2292917 | 4 | 9476375 | C | G | 27994 | -0.0800 | 0.0087 | 3.09E-20 |
| rs2139243 | 4 | 9476600 | A | G | 28045 | 0.1106 | 0.0104 | 3.12E-26 |
| rs883041 | 4 | 9477444 | T | C | 28007 | -0.1090 | 0.0103 | 4.31E-26 |
| rs13105954 | 4 | 9477515 | C | G | 27992 | -0.0799 | 0.0087 | 3.17E-20 |
| rs939134 | 4 | 9477691 | A | T | 28028 | 0.1086 | 0.0103 | 6.09E-26 |
| rs884573 | 4 | 9478832 | C | G | 27213 | -0.0990 | 0.0113 | 1.67E-18 |
| rs1048252 | 4 | 9479633 | T | C | 26676 | -0.1075 | 0.0139 | 9.02E-15 |
| rs1568318 | 4 | 9480639 | T | C | 28033 | 0.1194 | 0.0110 | 2.99E-27 |
| rs16889842 | 4 | 9481111 | A | G | 25225 | -0.1566 | 0.0286 | 4.30E-08 |
| rs2867394 | 4 | 9481573 | A | G | 28019 | -0.1256 | 0.0112 | 3.65E-29 |
| rs13148571 | 4 | 9482953 | A | C | 25798 | 0.0948 | 0.0097 | 1.75E-22 |
| rs1519098 | 4 | 9490256 | A | C | 28016 | 0.0778 | 0.0087 | 3.03E-19 |
| rs6824636 | 4 | 9490949 | C | G | 28038 | -0.1019 | 0.0097 | 5.52E-26 |
| rs6449090 | 4 | 9493634 | T | C | 28018 | 0.0774 | 0.0092 | 4.00E-17 |
| rs10939599 | 4 | 9495958 | A | C | 26151 | -0.1193 | 0.0114 | 1.94E-25 |
| rs10939600 | 4 | 9496029 | A | T | 27985 | 0.0781 | 0.0092 | 1.95E-17 |
| rs9993652 | 4 | 9497947 | C | G | 14495 | -0.0816 | 0.0121 | 1.50E-11 |
| rs6818572 | 4 | 9498546 | A | G | 27263 | -0.1185 | 0.0114 | 1.84E-25 |
| rs1107710 | 4 | 9499806 | A | G | 28036 | 0.1202 | 0.0111 | 2.71E-27 |
| rs938563 | 4 | 9500096 | C | G | 27937 | -0.1127 | 0.0109 | 4.78E-25 |
| rs938562 | 4 | 9500129 | T | C | 28051 | -0.1204 | 0.0111 | 1.39E-27 |
| rs10939602 | 4 | 9501200 | T | C | 27919 | 0.0774 | 0.0092 | 5.28E-17 |
| rs4697692 | 4 | 9502295 | T | C | 27858 | -0.1090 | 0.0118 | 2.43E-20 |
| rs12644047 | 4 | 9502501 | A | G | 27947 | -0.1287 | 0.0113 | 2.81E-30 |
| rs7669444 | 4 | 9502675 | A | G | 27819 | 0.0773 | 0.0093 | 8.72E-17 |
| rs4697693 | 4 | 9504958 | A | C | 26240 | 0.1082 | 0.0098 | 1.93E-28 |
| rs10939605 | 4 | 9505391 | A | C | 28031 | -0.1038 | 0.0094 | 3.29E-28 |
| rs4697694 | 4 | 9505740 | T | C | 28030 | 0.1032 | 0.0094 | 6.51E-28 |
| rs6449097 | 4 | 9505832 | T | C | 27856 | -0.0767 | 0.0081 | 2.38E-21 |
| rs13115121 | 4 | 9506440 | T | G | 27908 | -0.0767 | 0.0081 | 1.96E-21 |
| rs13129868 | 4 | 9506470 | A | G | 27926 | 0.0772 | 0.0081 | 1.07E-21 |
| rs13122026 | 4 | 9507305 | T | C | 27925 | -0.0778 | 0.0081 | 6.17E-22 |
| rs6449100 | 4 | 9510661 | T | C | 27809 | 0.0757 | 0.0082 | 2.30E-20 |
| rs4697910 | 4 | 9510972 | A | G | 27941 | -0.1026 | 0.0095 | 2.06E-27 |
| rs11732681 | 4 | 9511974 | A | G | 27762 | 0.0791 | 0.0081 | 1.90E-22 |
| rs11737685 | 4 | 9512219 | A | G | 27986 | -0.0983 | 0.0091 | 2.68E-27 |
| rs7694136 | 4 | 9517889 | C | G | 27912 | -0.0787 | 0.0081 | 2.51E-22 |
| rs2280202 | 4 | 9518393 | T | C | 27954 | -0.0792 | 0.0081 | 1.11E-22 |
| rs2280205 | 4 | 9519021 | A | G | 27972 | 0.0798 | 0.0081 | 5.08E-23 |
| rs11734893 | 4 | 9519539 | A | G | 27937 | 0.0798 | 0.0081 | 7.45E-23 |
| rs13103429 | 4 | 9519733 | T | C | 27936 | 0.0799 | 0.0081 | 9.53E-23 |
| rs13108825 | 4 | 9519761 | C | G | 27913 | -0.0806 | 0.0082 | 4.97E-23 |
| rs11722228 | 4 | 9524839 | T | C | 28021 | 0.1679 | 0.0091 | 1.75E-75 |
| rs4697695 | 4 | 9524948 | A | G | 27978 | 0.1688 | 0.0092 | 5.74E-76 |
| rs10516194 | 4 | 9525307 | T | C | 19672 | 0.3494 | 0.0394 | 7.25E-19 |
| rs10805346 | 4 | 9529445 | T | C | 27902 | 0.2020 | 0.0083 | 7.89E-131 |
| rs874432 | 4 | 9529704 | A | T | 26175 | -0.2971 | 0.0106 | 7.35E-173 |
| rs6823877 | 4 | 9531029 | T | C | 27975 | 0.1705 | 0.0091 | 7.32E-79 |
| rs16890979 | 4 | 9531265 | T | C | 26993 | -0.3051 | 0.0104 | 3.55E-189 |
| rs938564 | 4 | 9531671 | T | G | 27835 | 0.3132 | 0.0105 | 1.67E-195 |
| rs734553 | 4 | 9532102 | T | G | 27817 | 0.3146 | 0.0104 | 5.22E-201 |
| rs6832439 | 4 | 9533417 | A | G | 27819 | -0.3146 | 0.0105 | 3.08E-197 |
| rs938553 | 4 | 9534624 | T | C | 26717 | 0.0909 | 0.0154 | 3.63E-09 |
| rs938554 | 4 | 9534790 | C | G | 27819 | -0.3128 | 0.0106 | 2.18E-191 |
| rs938555 | 4 | 9535149 | A | G | 27821 | -0.3150 | 0.0105 | 9.81E-198 |
| rs10939614 | 4 | 9535711 | T | C | 27947 | 0.1707 | 0.0090 | 1.67E-79 |
| rs13129697 | 4 | 9536065 | T | G | 27812 | 0.2846 | 0.0096 | 3.84E-193 |
| rs6838021 | 4 | 9536718 | T | C | 27809 | -0.3142 | 0.0105 | 9.65E-197 |
| rs881971 | 4 | 9540060 | T | C | 27964 | 0.1441 | 0.0081 | 2.08E-71 |
| rs737267 | 4 | 9543842 | T | G | 27991 | -0.2960 | 0.0100 | 1.49E-192 |
| rs6855911 | 4 | 9545008 | A | G | 25983 | 0.2896 | 0.0102 | 2.53E-177 |
| rs7670751 | 4 | 9547871 | A | C | 14481 | 0.2723 | 0.0128 | 2.00E-100 |
| rs4447863 | 4 | 9548067 | T | C | 27923 | 0.1583 | 0.0080 | 3.14E-88 |
| rs938558 | 4 | 9548303 | A | G | 27925 | 0.2681 | 0.0094 | 6.38E-179 |
| rs4511996 | 4 | 9548916 | A | G | 27986 | 0.0817 | 0.0106 | 9.98E-15 |
| rs5028843 | 4 | 9549904 | A | G | 27964 | -0.2958 | 0.0099 | 3.71E-196 |
| rs4697913 | 4 | 9550360 | T | C | 27933 | -0.2978 | 0.0102 | 2.17E-187 |
| rs7675964 | 4 | 9550532 | T | C | 28024 | -0.2701 | 0.0094 | 1.43E-181 |
| rs4697698 | 4 | 9551675 | T | C | 28023 | 0.1557 | 0.0080 | 1.09E-84 |
| rs4292327 | 4 | 9552798 | A | G | 28013 | 0.0814 | 0.0105 | 1.00E-14 |
| rs12498742 | 4 | 9553150 | A | G | 28035 | 0.3081 | 0.0102 | 1.98E-200 |
| rs6449144 | 4 | 9553748 | T | G | 27796 | 0.1701 | 0.0090 | 1.53E-79 |
| rs4235346 | 4 | 9554394 | T | C | 26268 | -0.1549 | 0.0082 | 6.36E-79 |
| rs4697700 | 4 | 9554890 | C | G | 27674 | -0.2928 | 0.0104 | 2.15E-174 |
| rs4697701 | 4 | 9555193 | A | G | 27976 | -0.2841 | 0.0096 | 1.80E-192 |
| rs16891234 | 4 | 9555261 | T | C | 27970 | -0.0682 | 0.0103 | 4.22E-11 |
| rs4475146 | 4 | 9555754 | A | C | 28039 | -0.3113 | 0.0104 | 7.39E-197 |
| rs2018643 | 4 | 9556219 | T | C | 28043 | 0.1480 | 0.0082 | 1.69E-72 |
| rs1122141 | 4 | 9556376 | T | C | 27768 | 0.1506 | 0.0082 | 3.19E-76 |
| rs4621431 | 4 | 9556688 | A | G | 27913 | -0.1478 | 0.0082 | 5.26E-72 |
| rs4339211 | 4 | 9556756 | T | C | 28040 | -0.1413 | 0.0082 | 4.62E-66 |
| rs7694997 | 4 | 9556909 | A | G | 28016 | 0.1477 | 0.0082 | 3.15E-72 |
| rs7686538 | 4 | 9557175 | T | C | 28016 | -0.1477 | 0.0082 | 3.53E-72 |
| rs4580649 | 4 | 9557559 | A | G | 28019 | -0.1476 | 0.0082 | 3.93E-72 |
| rs998676 | 4 | 9557662 | T | C | 28000 | 0.1558 | 0.0080 | 8.30E-85 |
| rs998675 | 4 | 9557927 | T | C | 27880 | -0.1537 | 0.0080 | 4.71E-82 |
| rs12498150 | 4 | 9559635 | T | C | 28045 | -0.1473 | 0.0082 | 7.52E-72 |
| rs12498956 | 4 | 9559803 | A | C | 28035 | -0.1471 | 0.0082 | 2.99E-71 |
| rs13328050 | 4 | 9560218 | T | G | 27963 | -0.1466 | 0.0082 | 6.35E-71 |
| rs1079128 | 4 | 9560319 | T | C | 28045 | 0.1473 | 0.0082 | 8.46E-72 |
| rs9993410 | 4 | 9560362 | T | C | 28045 | -0.1473 | 0.0082 | 8.44E-72 |
| rs11723439 | 4 | 9560917 | T | C | 27375 | -0.3190 | 0.0111 | 1.25E-181 |
| rs4235347 | 4 | 9561054 | T | C | 28046 | -0.1471 | 0.0083 | 6.72E-71 |
| [rs4697914](http://www.ncbi.nlm.nih.gov/SNP/snp_ref.cgi?rs=4697914) | 4 | 9561364 | A | G | 13482 | -0.1693 | 0.0136 | 9.37E-36 |
| rs4455410 | 4 | 9562395 | T | C | 28038 | 0.1464 | 0.0082 | 1.98E-70 |
| rs4560411 | 4 | 9562459 | A | G | 28046 | 0.1458 | 0.0082 | 3.68E-70 |
| rs4447861 | 4 | 9563038 | T | C | 27838 | -0.1375 | 0.0085 | 7.24E-59 |
| rs4459990 | 4 | 9563103 | T | C | 28046 | 0.1459 | 0.0082 | 3.90E-70 |
| rs9994266 | 4 | 9563548 | A | G | 28024 | -0.1466 | 0.0083 | 2.06E-70 |
| rs7376948 | 4 | 9563806 | A | G | 28048 | -0.1470 | 0.0083 | 8.46E-71 |
| rs7375587 | 4 | 9563856 | A | T | 28028 | 0.1468 | 0.0083 | 1.31E-70 |
| rs7378305 | 4 | 9563991 | T | C | 28049 | -0.1470 | 0.0083 | 8.58E-71 |
| rs7375599 | 4 | 9564016 | A | G | 28015 | 0.1542 | 0.0080 | 3.94E-83 |
| rs7378340 | 4 | 9564296 | T | C | 28045 | -0.1457 | 0.0083 | 8.35E-70 |
| rs4519796 | 4 | 9565034 | A | G | 28049 | 0.1462 | 0.0082 | 1.35E-70 |
| rs4311316 | 4 | 9565069 | A | C | 27968 | -0.1462 | 0.0083 | 5.81E-70 |
| rs4481233 | 4 | 9565177 | T | C | 27956 | -0.3229 | 0.0111 | 4.79E-186 |
| rs4314284 | 4 | 9565194 | T | C | 28026 | -0.1469 | 0.0083 | 1.04E-70 |
| rs4312757 | 4 | 9565243 | A | T | 27819 | -0.1528 | 0.0085 | 3.06E-72 |
| rs6814664 | 4 | 9565326 | T | C | 28046 | -0.1470 | 0.0083 | 8.94E-71 |
| rs6449155 | 4 | 9565645 | T | G | 28046 | 0.1472 | 0.0083 | 5.97E-71 |
| rs6449156 | 4 | 9565810 | C | G | 27967 | -0.1517 | 0.0082 | 5.33E-76 |
| rs17245436 | 4 | 9567267 | A | G | 28025 | 0.1469 | 0.0082 | 4.89E-71 |
| rs17185835 | 4 | 9567278 | T | G | 28025 | 0.1470 | 0.0082 | 4.12E-71 |
| rs17185870 | 4 | 9567312 | A | C | 28025 | -0.1470 | 0.0083 | 5.45E-71 |
| rs11724510 | 4 | 9567681 | T | C | 28025 | -0.1470 | 0.0082 | 4.11E-71 |
| rs6815001 | 4 | 9567760 | C | G | 28025 | -0.1470 | 0.0082 | 4.64E-71 |
| rs6849717 | 4 | 9567817 | T | C | 27999 | 0.1550 | 0.0080 | 5.88E-84 |
| rs6849729 | 4 | 9567830 | T | C | 28025 | 0.1470 | 0.0082 | 4.41E-71 |
| rs6843873 | 4 | 9567886 | A | C | 28025 | 0.1470 | 0.0082 | 4.40E-71 |
| rs6850143 | 4 | 9568022 | T | C | 28025 | 0.1466 | 0.0082 | 8.89E-71 |
| rs6844316 | 4 | 9568075 | A | G | 28025 | 0.1469 | 0.0082 | 5.49E-71 |
| rs6834893 | 4 | 9568221 | T | C | 28025 | -0.1468 | 0.0082 | 2.98E-71 |
| rs10001964 | 4 | 9568373 | T | C | 26935 | -0.1377 | 0.0084 | 5.07E-61 |
| rs4515163 | 4 | 9568701 | T | C | 28025 | -0.1474 | 0.0082 | 1.89E-71 |
| rs6449157 | 4 | 9569540 | A | G | 28022 | -0.1473 | 0.0082 | 2.46E-71 |
| rs6449159 | 4 | 9569596 | A | G | 28022 | -0.1473 | 0.0082 | 2.46E-71 |
| rs7672947 | 4 | 9570466 | C | G | 28019 | -0.1472 | 0.0082 | 3.18E-71 |
| rs17245723 | 4 | 9571316 | A | T | 27935 | 0.1524 | 0.0082 | 8.30E-77 |
| rs11942223 | 4 | 9571863 | T | C | 28029 | 0.3178 | 0.0106 | 1.73E-197 |
| rs6823361 | 4 | 9572225 | A | G | 28013 | 0.1473 | 0.0083 | 2.87E-71 |
| rs6836706 | 4 | 9573349 | A | T | 28013 | -0.1473 | 0.0083 | 2.83E-71 |
| rs6850684 | 4 | 9573478 | T | G | 28013 | -0.1468 | 0.0083 | 1.10E-70 |
| rs10018204 | 4 | 9573668 | T | C | 28013 | -0.1473 | 0.0083 | 3.20E-71 |
| rs6839490 | 4 | 9574098 | C | G | 27485 | 0.1469 | 0.0084 | 1.70E-68 |
| rs6856127 | 4 | 9574541 | T | C | 28034 | 0.1473 | 0.0083 | 2.78E-71 |
| rs6840802 | 4 | 9574731 | C | G | 28033 | 0.1477 | 0.0083 | 4.39E-71 |
| rs6449171 | 4 | 9575096 | T | C | 28024 | 0.1475 | 0.0083 | 3.39E-71 |
| rs6449172 | 4 | 9575134 | A | T | 28036 | 0.1469 | 0.0083 | 1.31E-70 |
| rs6449173 | 4 | 9575203 | T | G | 28012 | 0.3136 | 0.0107 | 8.10E-189 |
| rs6847019 | 4 | 9575347 | T | C | 28042 | 0.0801 | 0.0104 | 1.44E-14 |
| rs7442295 | 4 | 9575478 | A | G | 27995 | 0.3166 | 0.0106 | 5.14E-196 |
| rs6449174 | 4 | 9575520 | T | C | 28035 | -0.1466 | 0.0083 | 2.35E-70 |
| rs9998811 | 4 | 9575575 | A | G | 28037 | -0.3173 | 0.0105 | 1.33E-200 |
| rs7658170 | 4 | 9575691 | T | C | 28029 | 0.1463 | 0.0083 | 5.23E-70 |
| rs6449175 | 4 | 9575708 | T | C | 28055 | -0.0828 | 0.0105 | 3.27E-15 |
| rs7663097 | 4 | 9575889 | T | C | 27939 | 0.1496 | 0.0081 | 3.52E-76 |
| rs7676733 | 4 | 9576054 | A | G | 28036 | -0.1470 | 0.0083 | 1.20E-70 |
| rs10017674 | 4 | 9576151 | T | C | 27754 | 0.1419 | 0.0082 | 2.14E-66 |
| rs7435196 | 4 | 9576654 | A | C | 28036 | 0.1471 | 0.0083 | 9.66E-71 |
| rs6449176 | 4 | 9576941 | A | G | 28036 | -0.1469 | 0.0083 | 1.29E-70 |
| rs6449178 | 4 | 9577782 | T | C | 28028 | -0.1466 | 0.0083 | 2.56E-70 |
| rs6449179 | 4 | 9578215 | A | G | 28036 | -0.1471 | 0.0083 | 9.88E-71 |
| rs7677710 | 4 | 9578615 | T | G | 28036 | 0.1470 | 0.0083 | 1.11E-70 |
| rs7683283 | 4 | 9579072 | T | C | 28036 | 0.1469 | 0.0083 | 1.44E-70 |
| rs7376960 | 4 | 9579668 | A | G | 27912 | 0.3175 | 0.0106 | 4.04E-197 |
| rs6449183 | 4 | 9579789 | A | C | 27899 | -0.1513 | 0.0081 | 9.44E-78 |
| rs4292328 | 4 | 9580060 | T | C | 27892 | -0.1557 | 0.0080 | 2.54E-84 |
| rs4473653 | 4 | 9580156 | A | G | 27909 | 0.1467 | 0.0083 | 3.85E-70 |
| rs7439210 | 4 | 9580847 | C | G | 27876 | 0.3149 | 0.0108 | 6.73E-187 |
| rs13103690 | 4 | 9581876 | T | G | 27899 | 0.1507 | 0.0081 | 3.60E-77 |
| rs13103879 | 4 | 9581977 | T | C | 27899 | 0.1509 | 0.0081 | 2.60E-77 |
| rs6852441 | 4 | 9582842 | T | C | 27905 | -0.1476 | 0.0083 | 4.07E-71 |
| rs6449201 | 4 | 9582992 | T | C | 27905 | -0.1470 | 0.0083 | 1.97E-70 |
| rs6449202 | 4 | 9583141 | T | C | 27905 | 0.1468 | 0.0083 | 2.82E-70 |
| rs1071988 | 4 | 9583736 | A | G | 27876 | 0.3138 | 0.0108 | 1.31E-185 |
| rs4505821 | 4 | 9587192 | A | G | 28052 | 0.0822 | 0.0106 | 6.99E-15 |
| rs16868246 | 4 | 9587403 | C | G | 27877 | -0.3141 | 0.0108 | 5.83E-186 |
| rs13103497 | 4 | 9588360 | A | G | 26126 | -0.1514 | 0.0089 | 1.08E-64 |
| rs13144899 | 4 | 9588400 | T | C | 28001 | -0.1128 | 0.0197 | 9.51E-09 |
| rs11723970 | 4 | 9589560 | T | C | 27147 | -0.1632 | 0.0083 | 1.96E-86 |
| rs11722229 | 4 | 9589795 | A | C | 27872 | 0.2944 | 0.0107 | 1.19E-166 |
| rs882223 | 4 | 9590723 | A | C | 26855 | -0.1615 | 0.0083 | 4.02E-85 |
| rs13131257 | 4 | 9590987 | T | C | 27881 | -0.3135 | 0.0108 | 2.94E-185 |
| rs13145758 | 4 | 9591095 | A | G | 27876 | 0.3097 | 0.0107 | 3.35E-184 |
| rs13125029 | 4 | 9591127 | A | G | 26718 | -0.1439 | 0.0087 | 3.52E-61 |
| rs13125209 | 4 | 9591142 | A | C | 27108 | -0.2918 | 0.0110 | 4.70E-155 |
| rs13115193 | 4 | 9591289 | T | C | 27906 | 0.1501 | 0.0080 | 9.19E-78 |
| rs13125646 | 4 | 9591428 | A | G | 26831 | -0.2865 | 0.0115 | 5.37E-137 |
| rs10003001 | 4 | 9593573 | T | C | 28021 | 0.0813 | 0.0105 | 1.15E-14 |
| rs10033612 | 4 | 9594104 | T | C | 27974 | 0.0817 | 0.0105 | 9.22E-15 |
| rs11723591 | 4 | 9594496 | A | T | 27908 | 0.1525 | 0.0080 | 3.50E-80 |
| rs7660895 | 4 | 9594543 | A | G | 27897 | -0.2836 | 0.0099 | 1.77E-180 |
| rs7680126 | 4 | 9594694 | A | G | 27902 | 0.3089 | 0.0106 | 1.07E-186 |
| rs17246501 | 4 | 9594808 | A | C | 27960 | -0.1533 | 0.0080 | 3.30E-81 |
| rs9992406 | 4 | 9595386 | T | C | 27151 | 0.0758 | 0.0105 | 6.08E-13 |
| rs4385059 | 4 | 9598331 | T | C | 28018 | 0.3193 | 0.0111 | 5.75E-182 |
| rs17187075 | 4 | 9599426 | C | G | 27980 | 0.1450 | 0.0081 | 9.13E-72 |
| rs10011206 | 4 | 9601053 | T | C | 28017 | 0.0817 | 0.0105 | 7.69E-15 |
| rs7678012 | 4 | 9602870 | T | C | 28016 | -0.1448 | 0.0081 | 6.06E-72 |
| rs7663032 | 4 | 9602936 | T | C | 14554 | 0.2699 | 0.0129 | 3.34E-97 |
| rs6449213 | 4 | 9603313 | T | C | 28006 | 0.3229 | 0.0111 | 4.79E-186 |
| rs3775948 | 4 | 9604280 | C | G | 27968 | 0.2833 | 0.0099 | 4.23E-180 |
| rs12499857 | 4 | 9604474 | A | G | 27927 | 0.1445 | 0.0081 | 1.40E-70 |
| rs3796842 | 4 | 9604949 | A | T | 27953 | -0.1460 | 0.0081 | 4.17E-73 |
| rs9998739 | 4 | 9605607 | A | G | 28038 | 0.0814 | 0.0105 | 9.18E-15 |
| rs13111638 | 4 | 9605988 | T | C | 26701 | -0.3152 | 0.0114 | 2.86E-168 |
| rs4547795 | 4 | 9606158 | T | C | 27923 | 0.0795 | 0.0105 | 4.78E-14 |
| rs4529048 | 4 | 9606210 | A | C | 27958 | 0.2793 | 0.0099 | 4.15E-175 |
| rs3733588 | 4 | 9606401 | A | G | 27949 | 0.2838 | 0.0099 | 9.94E-181 |
| rs3733587 | 4 | 9606532 | A | G | 28033 | -0.0818 | 0.0105 | 7.06E-15 |
| rs7669607 | 4 | 9606899 | T | C | 28014 | -0.3093 | 0.0106 | 3.56E-187 |
| rs10939650 | 4 | 9607538 | T | C | 28023 | 0.2837 | 0.0099 | 1.33E-180 |
| rs13113918 | 4 | 9607591 | A | G | 14521 | -0.2925 | 0.0138 | 1.05E-99 |
| rs10008035 | 4 | 9608433 | T | G | 28031 | 0.0787 | 0.0104 | 3.40E-14 |
| rs7696536 | 4 | 9609334 | T | G | 28027 | 0.0819 | 0.0105 | 6.53E-15 |
| rs1014290 | 4 | 9610959 | A | G | 28026 | 0.2843 | 0.0099 | 2.33E-181 |
| rs7696895 | 4 | 9611523 | T | C | 28028 | -0.0793 | 0.0105 | 3.52E-14 |
| rs9991278 | 4 | 9611763 | T | C | 27939 | -0.3021 | 0.0105 | 4.89E-182 |
| rs4622999 | 4 | 9612493 | C | G | 27963 | -0.1454 | 0.0081 | 1.03E-72 |
| rs17247314 | 4 | 9613841 | C | G | 27996 | -0.1424 | 0.0081 | 1.94E-69 |
| rs10023068 | 4 | 9613930 | A | G | 28042 | -0.2556 | 0.0109 | 1.34E-121 |
| rs6853437 | 4 | 9614533 | A | G | 28037 | 0.2576 | 0.0109 | 1.76E-123 |
| rs10022499 | 4 | 9615635 | A | C | 28038 | 0.2557 | 0.0109 | 1.08E-121 |
| rs9291640 | 4 | 9616184 | T | C | 28040 | 0.2562 | 0.0109 | 3.66E-122 |
| rs9291642 | 4 | 9616373 | T | C | 28027 | 0.2993 | 0.0125 | 1.07E-126 |
| rs4543113 | 4 | 9617403 | A | G | 27997 | 0.1333 | 0.0084 | 6.46E-57 |
| rs6845554 | 4 | 9622271 | T | G | 27887 | -0.1370 | 0.0083 | 1.27E-61 |
| rs3756236 | 4 | 9622561 | A | T | 28014 | 0.1418 | 0.0081 | 3.62E-69 |
| rs6827754 | 4 | 9627251 | A | C | 27926 | 0.1388 | 0.0083 | 9.58E-63 |
| rs13133766 | 4 | 9628830 | T | C | 28025 | 0.1427 | 0.0081 | 4.84E-70 |
| rs2240720 | 4 | 9629578 | T | C | 28025 | 0.1427 | 0.0081 | 4.37E-70 |
| rs2240721 | 4 | 9629662 | A | G | 28025 | 0.1372 | 0.0081 | 6.35E-65 |
| rs2240724 | 4 | 9630388 | C | G | 27975 | 0.1407 | 0.0081 | 3.50E-68 |
| rs6849273 | 4 | 9630693 | T | C | 28009 | 0.1377 | 0.0082 | 7.47E-63 |
| rs12509955 | 4 | 9633401 | T | C | 28008 | -0.2837 | 0.0107 | 6.68E-155 |
| rs3775940 | 4 | 9634261 | A | T | 28022 | 0.1389 | 0.0082 | 7.00E-64 |
| rs6826764 | 4 | 9639892 | C | G | 28044 | 0.2503 | 0.0112 | 1.26E-110 |
| rs6856396 | 4 | 9640261 | A | T | 28035 | -0.2972 | 0.0125 | 5.92E-125 |
| rs12506455 | 4 | 9640667 | A | T | 28005 | 0.1404 | 0.0081 | 6.35E-68 |
| rs10939663 | 4 | 9641614 | T | G | 27558 | -0.1578 | 0.0099 | 1.13E-57 |
| rs12506122 | 4 | 9642636 | A | C | 28002 | 0.1379 | 0.0083 | 2.19E-62 |
| rs13146686 | 4 | 9644031 | T | C | 27944 | -0.1354 | 0.0083 | 3.61E-60 |
| rs11722930 | 4 | 9644552 | A | G | 28013 | 0.1403 | 0.0081 | 8.38E-68 |
| rs10006397 | 4 | 9645238 | A | C | 28032 | 0.2506 | 0.0112 | 6.90E-111 |
| rs11727199 | 4 | 9645288 | T | C | 28011 | 0.1404 | 0.0081 | 6.18E-68 |
| rs3733585 | 4 | 9645437 | A | G | 28008 | -0.1405 | 0.0081 | 4.98E-68 |
| rs11731110 | 4 | 9646444 | T | C | 28011 | -0.1405 | 0.0081 | 4.63E-68 |
| rs10939665 | 4 | 9646726 | T | C | 28006 | -0.1403 | 0.0081 | 6.86E-68 |
| rs10012779 | 4 | 9647210 | T | C | 27960 | -0.1767 | 0.0174 | 2.97E-24 |
| rs13139055 | 4 | 9648022 | T | G | 27945 | 0.1380 | 0.0083 | 1.13E-62 |
| rs13115776 | 4 | 9649287 | C | G | 27945 | -0.1380 | 0.0083 | 1.26E-62 |
| rs12508991 | 4 | 9650202 | T | C | 26204 | 0.1407 | 0.0083 | 2.67E-64 |
| rs10029311 | 4 | 9650232 | T | C | 27960 | 0.1703 | 0.0172 | 4.29E-23 |
| rs7679916 | 4 | 9651258 | T | C | 27946 | 0.1403 | 0.0081 | 1.60E-67 |
| rs7349721 | 4 | 9651660 | A | T | 28004 | -0.2501 | 0.0112 | 1.87E-110 |
| rs13101785 | 4 | 9652013 | A | T | 27946 | 0.1402 | 0.0081 | 1.72E-67 |
| rs13137343 | 4 | 9652126 | A | C | 27946 | 0.1403 | 0.0081 | 1.47E-67 |
| rs13110307 | 4 | 9653462 | T | C | 27941 | -0.1380 | 0.0082 | 6.88E-63 |
| rs13129453 | 4 | 9653882 | T | C | 27940 | -0.1380 | 0.0082 | 7.81E-63 |
| rs4529049 | 4 | 9654487 | T | C | 27940 | 0.1369 | 0.0082 | 6.82E-62 |
| rs4637402 | 4 | 9654528 | T | C | 2138 | 0.3144 | 0.0393 | 1.22E-15 |
| [rs4637402](http://www.ncbi.nlm.nih.gov/SNP/snp_ref.cgi?rs=4637402) | 4 | 9654528 | T | C | 13482 | 0.2863 | 0.0162 | 8.70E-70 |
| rs10939669 | 4 | 9654925 | A | G | 27945 | -0.1404 | 0.0081 | 7.44E-68 |
| rs4608811 | 4 | 9658773 | A | C | 7999 | -0.2554 | 0.0201 | 8.17E-37 |
| rs733175 | 4 | 9659239 | T | C | 28008 | 0.2511 | 0.0112 | 2.53E-111 |
| rs6829727 | 4 | 9660770 | A | C | 7994 | -0.1424 | 0.0159 | 2.86E-19 |
| rs13120348 | 4 | 9662253 | C | G | 27982 | -0.1395 | 0.0083 | 3.77E-64 |
| rs7671266 | 4 | 9665474 | T | C | 27902 | -0.2817 | 0.0107 | 9.39E-153 |
| rs10516198 | 4 | 9668546 | T | C | 27727 | -0.0647 | 0.0106 | 1.03E-09 |
| rs714873 | 4 | 9668716 | A | G | 27964 | 0.1998 | 0.0106 | 5.40E-80 |
| rs6834555 | 4 | 9671424 | A | G | 27915 | 0.2001 | 0.0105 | 3.23E-80 |
| rs12506004 | 4 | 9675968 | A | G | 27836 | -0.1307 | 0.0094 | 3.67E-44 |
| rs16868313 | 4 | 9677162 | T | C | 27825 | 0.1323 | 0.0094 | 3.42E-45 |
| rs4320137 | 4 | 9682067 | T | C | 27792 | 0.2827 | 0.0125 | 3.02E-113 |
| rs4461524 | 4 | 9683268 | A | T | 26999 | -0.0990 | 0.0110 | 2.02E-19 |
| rs11731597 | 4 | 9684583 | T | C | 27869 | 0.1291 | 0.0094 | 3.92E-43 |
| rs9926 | 4 | 9685958 | A | G | 14303 | 0.1482 | 0.0140 | 4.94E-26 |
| rs10516200 | 4 | 9691254 | A | C | 27960 | 0.1270 | 0.0094 | 7.36E-42 |
| rs3756230 | 4 | 9692927 | C | G | 27967 | -0.1471 | 0.0104 | 9.14E-46 |
| rs2241470 | 4 | 9693668 | T | G | 14470 | -0.1135 | 0.0122 | 1.36E-20 |
| rs2241473 | 4 | 9695047 | A | G | 27970 | 0.1398 | 0.0102 | 1.06E-42 |
| rs2241475 | 4 | 9695286 | T | G | 7917 | -0.1111 | 0.0175 | 2.10E-10 |
| rs3756227 | 4 | 9697093 | A | C | 27977 | 0.1418 | 0.0102 | 1.37E-43 |
| rs2241480 | 4 | 9698861 | T | C | 28043 | 0.1259 | 0.0094 | 3.54E-41 |
| rs734122 | 4 | 9698963 | A | G | 28019 | 0.1276 | 0.0093 | 1.67E-42 |
| rs3822242 | 4 | 9704002 | T | C | 27967 | 0.1171 | 0.0080 | 5.43E-49 |
| rs11727087 | 4 | 9705118 | T | C | 28026 | -0.1266 | 0.0083 | 4.91E-53 |
| rs3796818 | 4 | 9707074 | T | C | 27987 | 0.1478 | 0.0104 | 4.91E-46 |
| rs11726271 | 4 | 9707290 | A | G | 27959 | -0.1198 | 0.0080 | 7.81E-51 |
| rs2241483 | 4 | 9708929 | A | G | 27970 | 0.1202 | 0.0080 | 3.23E-51 |
| rs2241486 | 4 | 9710181 | C | G | 27806 | -0.1277 | 0.0094 | 2.51E-42 |
| rs2241488 | 4 | 9710229 | T | C | 27816 | -0.1405 | 0.0102 | 1.71E-43 |
| rs6830786 | 4 | 9710541 | T | C | 27345 | 0.1291 | 0.0094 | 1.33E-42 |
| rs16868326 | 4 | 9711691 | A | T | 27787 | 0.1252 | 0.0093 | 5.81E-41 |
| rs717615 | 4 | 9713768 | A | G | 27980 | 0.1167 | 0.0078 | 4.49E-50 |
| rs717614 | 4 | 9713886 | C | G | 23651 | 0.1160 | 0.0083 | 1.10E-44 |
| rs3756223 | 4 | 9714895 | T | C | 27612 | 0.0941 | 0.0084 | 4.05E-29 |
| rs12509714 | 4 | 9716189 | C | G | 27827 | -0.1721 | 0.0085 | 3.77E-91 |
| rs4459989 | 4 | 9721700 | T | C | 27832 | 0.0672 | 0.0106 | 2.14E-10 |
| rs2241468 | 4 | 9723003 | A | G | 27510 | 0.1465 | 0.0107 | 9.89E-43 |
| rs4604059 | 4 | 9724163 | T | C | 27866 | -0.1296 | 0.0080 | 9.51E-59 |
| rs12498927 | 4 | 9724621 | A | G | 27927 | -0.1313 | 0.0080 | 5.24E-61 |
| rs10939710 | 4 | 9725899 | T | C | 27790 | -0.0618 | 0.0096 | 1.21E-10 |
| rs3822236 | 4 | 9729059 | T | G | 27829 | 0.1420 | 0.0085 | 4.40E-63 |
| rs12374320 | 4 | 9729707 | T | C | 27911 | 0.0478 | 0.0086 | 3.14E-08 |
| rs4697708 | 4 | 9730287 | T | C | 27966 | 0.1386 | 0.0084 | 1.39E-60 |
| rs3756215 | 4 | 9730358 | A | G | 14482 | -0.1267 | 0.0136 | 1.10E-20 |
| rs4697710 | 4 | 9731747 | T | C | 27977 | 0.1387 | 0.0084 | 9.93E-61 |
| rs6825888 | 4 | 9731832 | T | C | 27983 | -0.1313 | 0.0102 | 5.85E-38 |
| rs4235354 | 4 | 9732040 | A | C | 12737 | -0.2802 | 0.0374 | 6.84E-14 |
| rs4235355 | 4 | 9732176 | A | C | 27981 | -0.1383 | 0.0084 | 2.61E-60 |
| rs4235356 | 4 | 9732204 | C | G | 27981 | -0.1389 | 0.0084 | 7.05E-61 |
| rs12506893 | 4 | 9732763 | T | C | 28002 | 0.1299 | 0.0102 | 2.13E-37 |
| rs10516201 | 4 | 9733039 | T | C | 28015 | -0.1301 | 0.0102 | 2.75E-37 |
| rs4697926 | 4 | 9733665 | A | C | 28034 | 0.1240 | 0.0084 | 1.50E-49 |
| rs4444830 | 4 | 9733917 | T | C | 25891 | 0.1223 | 0.0086 | 2.02E-45 |
| rs4456954 | 4 | 9733936 | C | G | 27575 | 0.1388 | 0.0122 | 3.72E-30 |
| rs715979 | 4 | 9734340 | C | G | 27851 | -0.1297 | 0.0106 | 1.66E-34 |
| rs3886038 | 4 | 9734353 | T | C | 28041 | 0.1309 | 0.0103 | 2.68E-37 |
| rs7699512 | 4 | 9734906 | T | C | 27945 | -0.1184 | 0.0081 | 5.23E-48 |
| rs7699671 | 4 | 9734972 | T | C | 28019 | -0.1287 | 0.0084 | 3.38E-53 |
| rs11722989 | 4 | 9735237 | A | G | 27966 | 0.1180 | 0.0081 | 1.24E-47 |
| rs11723016 | 4 | 9735287 | A | G | 27961 | 0.1149 | 0.0082 | 4.68E-45 |
| rs6449286 | 4 | 9735797 | A | T | 27153 | 0.1174 | 0.0094 | 6.98E-36 |
| rs4619888 | 4 | 9736076 | A | C | 27574 | -0.1350 | 0.0102 | 4.92E-40 |
| rs4467562 | 4 | 9736239 | A | G | 27498 | 0.1199 | 0.0092 | 8.84E-39 |
| rs10001106 | 4 | 9736539 | T | C | 27489 | -0.1195 | 0.0092 | 1.92E-38 |
| rs17250843 | 4 | 9737076 | C | G | 27903 | -0.0612 | 0.0105 | 5.58E-09 |
| rs715260 | 4 | 9737244 | C | G | 27546 | -0.1347 | 0.0102 | 8.90E-40 |
| rs7667775 | 4 | 9737634 | T | C | 27267 | 0.1338 | 0.0104 | 5.68E-38 |
| rs12502556 | 4 | 9739603 | C | G | 27193 | -0.1453 | 0.0103 | 4.87E-45 |
| rs10009493 | 4 | 9741145 | C | G | 27872 | 0.2584 | 0.0110 | 5.05E-122 |
| rs12501597 | 4 | 9741235 | T | G | 27857 | 0.1369 | 0.0101 | 5.66E-42 |
| rs6449289 | 4 | 9741641 | T | C | 25724 | 0.1606 | 0.0113 | 1.81E-45 |
| rs881641 | 4 | 9742845 | A | G | 27900 | 0.1416 | 0.0102 | 4.49E-44 |
| rs881642 | 4 | 9742971 | T | C | 27894 | 0.1393 | 0.0101 | 1.25E-43 |
| rs881643 | 4 | 9743215 | A | G | 27886 | 0.1353 | 0.0098 | 3.68E-43 |
| rs17197769 | 4 | 9743434 | T | C | 27911 | 0.1413 | 0.0102 | 5.85E-44 |
| rs1109472 | 4 | 9743546 | C | G | 27898 | 0.1426 | 0.0102 | 8.75E-45 |
| rs11938608 | 4 | 9744947 | T | C | 27839 | -0.1369 | 0.0098 | 3.03E-44 |
| rs4358401 | 4 | 9745905 | A | T | 27877 | -0.0501 | 0.0082 | 1.01E-09 |
| rs873984 | 4 | 9746223 | C | G | 27812 | 0.0562 | 0.0081 | 3.97E-12 |
| rs4399989 | 4 | 9746486 | A | G | 27877 | -0.0516 | 0.0082 | 2.73E-10 |
| rs4315785 | 4 | 9746705 | A | C | 27877 | 0.0521 | 0.0082 | 2.22E-10 |
| rs4235357 | 4 | 9746854 | T | C | 27880 | 0.0510 | 0.0082 | 4.82E-10 |
| rs12507725 | 4 | 9746888 | A | T | 27873 | 0.1418 | 0.0101 | 1.07E-44 |
| rs17198113 | 4 | 9747568 | T | C | 27876 | 0.1368 | 0.0100 | 1.23E-42 |
| rs11732828 | 4 | 9747844 | T | C | 27877 | 0.0525 | 0.0082 | 1.46E-10 |
| rs10939722 | 4 | 9748145 | T | G | 27807 | -0.0525 | 0.0082 | 1.69E-10 |
| rs10939723 | 4 | 9748203 | T | G | 27577 | -0.2412 | 0.0109 | 1.68E-108 |
| rs6449300 | 4 | 9748649 | T | C | 27872 | 0.1420 | 0.0101 | 7.16E-45 |
| rs6850516 | 4 | 9749076 | C | G | 27879 | -0.0517 | 0.0082 | 2.94E-10 |
| rs10025456 | 4 | 9749357 | T | C | 27827 | -0.0463 | 0.0080 | 7.63E-09 |
| rs10022911 | 4 | 9749649 | A | G | 27591 | 0.2463 | 0.0109 | 4.71E-113 |
| rs10020887 | 4 | 9749849 | C | G | 27958 | 0.2565 | 0.0110 | 2.90E-120 |
| rs10012288 | 4 | 9750051 | A | G | 27893 | -0.0530 | 0.0082 | 9.69E-11 |
| rs17198547 | 4 | 9750517 | T | C | 27603 | -0.2532 | 0.0111 | 3.58E-115 |
| rs10805356 | 4 | 9750700 | T | G | 27901 | 0.0531 | 0.0082 | 8.73E-11 |
| rs4473652 | 4 | 9750715 | C | G | 27996 | 0.0585 | 0.0105 | 2.40E-08 |
| rs10028503 | 4 | 9751025 | A | G | 27714 | 0.0524 | 0.0082 | 1.91E-10 |
| rs10015494 | 4 | 9751088 | A | G | 27913 | -0.0530 | 0.0082 | 9.60E-11 |
| rs10028937 | 4 | 9751464 | A | G | 27910 | 0.0521 | 0.0082 | 1.99E-10 |
| rs10015872 | 4 | 9751508 | A | G | 26127 | -0.0522 | 0.0085 | 6.55E-10 |
| rs17251963 | 4 | 9751659 | T | C | 28001 | 0.2640 | 0.0111 | 4.92E-125 |
| rs10031453 | 4 | 9751757 | A | G | 27909 | 0.0532 | 0.0082 | 6.73E-11 |
| rs4697713 | 4 | 9752680 | T | C | 27952 | 0.0497 | 0.0081 | 8.97E-10 |
| rs4697930 | 4 | 9752692 | C | G | 27954 | 0.0509 | 0.0081 | 3.11E-10 |
| rs4697714 | 4 | 9752884 | A | T | 27970 | -0.2556 | 0.0110 | 1.95E-119 |
| rs4292329 | 4 | 9753557 | C | G | 28024 | -0.0599 | 0.0105 | 1.17E-08 |
| rs4697931 | 4 | 9754394 | T | C | 28041 | -0.0519 | 0.0081 | 1.41E-10 |
| rs4574408 | 4 | 9754666 | A | G | 27919 | -0.0493 | 0.0081 | 1.23E-09 |
| rs4640669 | 4 | 9754831 | A | G | 27898 | -0.2479 | 0.0108 | 1.35E-116 |
| rs4484300 | 4 | 9754892 | A | G | 28022 | -0.0509 | 0.0081 | 3.08E-10 |
| rs4401449 | 4 | 9755096 | T | C | 28038 | -0.0496 | 0.0081 | 9.66E-10 |
| rs10939730 | 4 | 9755147 | C | G | 28038 | -0.0504 | 0.0081 | 5.15E-10 |
| rs4697933 | 4 | 9755591 | A | G | 27946 | -0.2493 | 0.0109 | 8.92E-116 |
| rs6855657 | 4 | 9757246 | T | C | 27996 | 0.0482 | 0.0081 | 2.53E-09 |
| rs10939732 | 4 | 9757488 | A | G | 28011 | -0.0499 | 0.0081 | 6.91E-10 |
| rs12507586 | 4 | 9757673 | A | G | 27956 | 0.1423 | 0.0101 | 5.29E-45 |
| rs4168 | 4 | 9757707 | A | C | 28019 | -0.0485 | 0.0081 | 2.23E-09 |
| rs11735668 | 4 | 9757753 | C | G | 27948 | 0.2424 | 0.0109 | 1.46E-109 |
| rs12508413 | 4 | 9757769 | A | G | 27956 | 0.1423 | 0.0101 | 4.71E-45 |
| rs6813334 | 4 | 9757851 | T | G | 27956 | -0.1423 | 0.0101 | 4.30E-45 |
| rs4697934 | 4 | 9758162 | T | C | 28023 | -0.0470 | 0.0081 | 6.80E-09 |
| rs4697936 | 4 | 9758693 | T | G | 27040 | -0.0966 | 0.0097 | 2.33E-23 |
| rs4697937 | 4 | 9758767 | T | G | 27946 | 0.0542 | 0.0083 | 6.03E-11 |
| rs6840883 | 4 | 9759962 | A | G | 27847 | -0.0457 | 0.0082 | 2.02E-08 |
| rs11721682 | 4 | 9760245 | T | C | 27792 | -0.2549 | 0.0111 | 1.07E-116 |
| rs6827946 | 4 | 9760433 | T | C | 27802 | 0.2406 | 0.0108 | 6.07E-110 |
| rs6827496 | 4 | 9760754 | A | G | 27921 | -0.1433 | 0.0101 | 5.90E-46 |
| rs6847379 | 4 | 9760802 | T | G | 27832 | -0.0471 | 0.0082 | 8.25E-09 |
| rs4697940 | 4 | 9761427 | C | G | 27818 | -0.0466 | 0.0082 | 1.08E-08 |
| rs731069 | 4 | 9761529 | A | C | 27865 | 0.0451 | 0.0081 | 3.15E-08 |
| rs731070 | 4 | 9761680 | T | C | 27791 | -0.0450 | 0.0082 | 3.60E-08 |
| rs747356 | 4 | 9762149 | T | G | 27753 | -0.0464 | 0.0082 | 1.29E-08 |
| rs6851524 | 4 | 9764139 | A | C | 27653 | 0.0585 | 0.0107 | 4.60E-08 |
| rs12513376 | 4 | 9765320 | T | C | 27735 | 0.1347 | 0.0100 | 4.87E-41 |
| rs11929718 | 4 | 9765419 | A | C | 27866 | -0.1315 | 0.0091 | 1.45E-47 |
| rs6834055 | 4 | 9767609 | A | T | 26538 | 0.2240 | 0.0107 | 2.59E-97 |
| rs2241464 | 4 | 9768059 | A | G | 27466 | 0.0666 | 0.0102 | 6.30E-11 |
| rs2241465 | 4 | 9768118 | T | C | 27680 | -0.1405 | 0.0094 | 6.27E-51 |
| rs4697941 | 4 | 9769960 | A | C | 27946 | -0.1342 | 0.0092 | 2.40E-48 |
| rs17450260 | 4 | 9772887 | A | T | 27882 | -0.1435 | 0.0100 | 1.71E-46 |
| rs6816215 | 4 | 9773125 | T | C | 27761 | -0.0815 | 0.0091 | 2.32E-19 |
| rs17450372 | 4 | 9773442 | T | C | 27879 | -0.1434 | 0.0100 | 1.96E-46 |
| rs1009144 | 4 | 9774029 | T | G | 27879 | 0.1454 | 0.0102 | 3.62E-46 |
| rs17450434 | 4 | 9774059 | T | C | 27879 | 0.1440 | 0.0102 | 2.40E-45 |
| rs16894270 | 4 | 9774877 | T | G | 27863 | -0.0722 | 0.0091 | 1.77E-15 |
| rs11729371 | 4 | 9775055 | A | T | 27990 | -0.2397 | 0.0169 | 2.02E-45 |
| rs11724641 | 4 | 9776092 | A | G | 27662 | -0.1305 | 0.0096 | 1.66E-42 |
| rs7659717 | 4 | 9776315 | T | C | 27786 | -0.1453 | 0.0100 | 4.29E-48 |
| rs17385112 | 4 | 9776630 | T | G | 27574 | 0.2407 | 0.0135 | 6.16E-71 |
| rs17385294 | 4 | 9777545 | A | G | 27802 | 0.1461 | 0.0102 | 1.19E-46 |
| rs1001216 | 4 | 9777947 | A | G | 27579 | 0.2390 | 0.0134 | 6.70E-71 |
| rs1001217 | 4 | 9778097 | C | G | 27584 | 0.0578 | 0.0088 | 5.44E-11 |
| rs11734209 | 4 | 9779469 | A | T | 27601 | 0.0615 | 0.0102 | 1.55E-09 |
| rs874079 | 4 | 9780203 | T | C | 27678 | -0.0552 | 0.0086 | 1.49E-10 |
| rs4697948 | 4 | 9780665 | C | G | 27619 | 0.0579 | 0.0088 | 4.87E-11 |
| rs2080076 | 4 | 9781043 | A | C | 27743 | -0.0568 | 0.0085 | 3.05E-11 |
| rs2098234 | 4 | 9781211 | C | G | 27743 | -0.0557 | 0.0085 | 6.65E-11 |
| rs6835689 | 4 | 9781577 | T | C | 27634 | -0.0582 | 0.0088 | 4.22E-11 |
| [rs10034405](http://www.ncbi.nlm.nih.gov/SNP/snp_ref.cgi?rs=10034405) | 4 | 9782794 | A | G | 13482 | 0.2111 | 0.0233 | 1.45E-19 |
| rs4697718 | 4 | 9783237 | T | C | 13482 | 0.1416 | 0.0145 | 1.60E-22 |
| rs4697719 | 4 | 9783256 | A | G | 7895 | -0.1244 | 0.0175 | 1.28E-12 |
| [rs4697719](http://www.ncbi.nlm.nih.gov/SNP/snp_ref.cgi?rs=4697719) | 4 | 9783256 | A | G | 13482 | -0.1433 | 0.0143 | 9.98E-24 |
| rs4697721 | 4 | 9783315 | C | G | 27671 | -0.1350 | 0.0092 | 4.07E-49 |
| [rs4697954](http://www.ncbi.nlm.nih.gov/SNP/snp_ref.cgi?rs=4697954) | 4 | 9783627 | A | G | 13482 | 0.1434 | 0.0145 | 4.69E-23 |
| rs10017447 | 4 | 9784634 | A | C | 13482 | -0.1458 | 0.0147 | 3.48E-23 |
| [rs10024152](http://www.ncbi.nlm.nih.gov/SNP/snp_ref.cgi?rs=10024152) | 4 | [9784787](http://www.ncbi.nlm.nih.gov/sites/entrez?term=NT_006316.15&db=nuccore&dopt=graph&m=851047&v=850997:851097&c=3366FF&theme=Details&flip=false&select=null&content=5&color=0&decor=0&layout=0&spacing=0) | A | T | 13482 | 0.1433 | 0.0143 | 1.37E-23 |
| rs2903827 | 4 | 9784970 | C | G | 27617 | 0.2666 | 0.0109 | 4.06E-132 |
| rs2868414 | 4 | 9786218 | C | G | 27990 | -0.1319 | 0.0091 | 4.66E-48 |
| rs17385872 | 4 | 9787266 | A | C | 27884 | 0.1425 | 0.0099 | 8.47E-47 |
| rs16894579 | 4 | 9787315 | T | C | 28050 | -0.1311 | 0.0091 | 1.60E-47 |
| rs10033955 | 4 | 9787654 | A | T | 28052 | -0.1306 | 0.0090 | 2.80E-47 |
| rs11737347 | 4 | 9788020 | A | T | 28052 | 0.1311 | 0.0090 | 1.55E-47 |
| rs7657551 | 4 | 9788407 | T | C | 28051 | 0.1310 | 0.0091 | 1.70E-47 |
| rs6449342 | 4 | 9788867 | A | G | 28048 | 0.1308 | 0.0091 | 2.59E-47 |
| rs4273473 | 4 | 9789720 | T | C | 28040 | 0.1309 | 0.0090 | 1.91E-47 |
| rs11724112 | 4 | 9789741 | T | C | 27777 | 0.1538 | 0.0081 | 8.49E-80 |
| rs6811287 | 4 | 9789921 | T | C | 27778 | -0.1547 | 0.0081 | 1.90E-80 |
| rs723663 | 4 | 9790472 | A | G | 28054 | 0.1316 | 0.0090 | 5.76E-48 |
| rs12500891 | 4 | 9790485 | A | T | 28020 | 0.1401 | 0.0099 | 6.99E-46 |
| rs4697956 | 4 | 9790770 | T | C | 28042 | -0.1311 | 0.0091 | 1.49E-47 |
| rs4697957 | 4 | 9791352 | A | G | 27705 | 0.2678 | 0.0108 | 9.83E-136 |
| rs4697958 | 4 | 9791654 | T | C | 28038 | 0.1303 | 0.0090 | 4.95E-47 |
| rs887735 | 4 | 9791943 | T | C | 27755 | -0.1438 | 0.0099 | 8.17E-48 |
| rs887733 | 4 | 9792206 | T | C | 27859 | 0.1174 | 0.0083 | 1.74E-45 |
| rs887732 | 4 | 9792215 | C | G | 27702 | -0.2685 | 0.0108 | 1.96E-136 |
| rs887731 | 4 | 9792284 | A | C | 28039 | 0.1310 | 0.0091 | 1.89E-47 |
| rs887729 | 4 | 9792709 | C | G | 28005 | -0.1395 | 0.0099 | 2.73E-45 |
| rs887728 | 4 | 9792896 | A | G | 28040 | 0.1310 | 0.0091 | 1.85E-47 |
| rs887727 | 4 | 9792917 | A | G | 28014 | -0.1327 | 0.0092 | 3.62E-47 |
| rs11722345 | 4 | 9795897 | A | C | 28034 | -0.1305 | 0.0091 | 4.04E-47 |
| rs4697960 | 4 | 9796361 | C | G | 28014 | -0.1310 | 0.0092 | 5.88E-46 |
| rs4697727 | 4 | 9797020 | T | C | 28007 | 0.1309 | 0.0091 | 7.22E-47 |
| rs956312 | 4 | 9797424 | A | G | 28040 | -0.1293 | 0.0088 | 1.25E-48 |
| rs956311 | 4 | 9797479 | A | C | 28029 | -0.1290 | 0.0088 | 1.22E-48 |
| rs11721530 | 4 | 9798260 | A | G | 27983 | -0.1291 | 0.0091 | 4.54E-46 |
| rs4697964 | 4 | 9798910 | A | G | 28036 | 0.1304 | 0.0091 | 6.65E-47 |
| rs4697965 | 4 | 9799156 | A | G | 28019 | 0.1300 | 0.0091 | 1.07E-46 |
| rs4697966 | 4 | 9799172 | A | G | 28021 | -0.1291 | 0.0091 | 5.31E-46 |
| rs2215691 | 4 | 9801206 | T | C | 28019 | -0.1288 | 0.0091 | 2.51E-45 |
| rs10489080 | 4 | 9802525 | C | G | 28019 | -0.1386 | 0.0099 | 6.56E-45 |
| rs2159864 | 4 | 9802702 | A | G | 27902 | 0.1267 | 0.0091 | 1.23E-43 |
| rs6826383 | 4 | 9803602 | A | G | 28040 | 0.1293 | 0.0091 | 2.37E-45 |
| rs4697728 | 4 | 9805736 | T | C | 28039 | 0.1295 | 0.0091 | 3.60E-46 |
| rs917825 | 4 | 9805859 | A | T | 28028 | 0.1284 | 0.0091 | 1.73E-45 |
| rs1860903 | 4 | 9805951 | A | G | 28039 | 0.1407 | 0.0099 | 5.24E-46 |
| rs929575 | 4 | 9805984 | C | G | 27873 | 0.2686 | 0.0109 | 4.46E-134 |
| rs917823 | 4 | 9806323 | T | C | 28031 | -0.1292 | 0.0091 | 5.88E-46 |
| rs4697968 | 4 | 9807726 | T | C | 8019 | -0.1207 | 0.0174 | 3.62E-12 |
| [rs4697968](http://www.ncbi.nlm.nih.gov/SNP/snp_ref.cgi?rs=4697968) | 4 | [9807726](http://www.ncbi.nlm.nih.gov/sites/entrez?term=NT_006316.15&db=nuccore&dopt=graph&m=873986&v=873936:874036&c=3366FF&theme=Details&flip=false&select=null&content=5&color=0&decor=0&layout=0&spacing=0) | T | C | 13482 | -0.1410 | 0.0141 | 1.38E-23 |
| [rs4697969](http://www.ncbi.nlm.nih.gov/SNP/snp_ref.cgi?rs=4697969) | 4 | [9808079](http://www.ncbi.nlm.nih.gov/sites/entrez?term=NT_006316.15&db=nuccore&dopt=graph&m=874339&v=874289:874389&c=3366FF&theme=Details&flip=false&select=null&content=5&color=0&decor=0&layout=0&spacing=0) | C | G | 13482 | -0.1412 | 0.0141 | 1.18E-23 |
| rs2080075 | 4 | 9808546 | T | C | 28019 | 0.1288 | 0.0091 | 1.16E-45 |
| rs4697729 | 4 | 9809499 | T | G | 28029 | -0.1289 | 0.0091 | 1.04E-45 |
| rs4697730 | 4 | 9809594 | T | C | 28001 | 0.1282 | 0.0091 | 3.58E-45 |
| rs4697971 | 4 | 9809958 | A | T | 28028 | -0.1284 | 0.0091 | 1.88E-45 |
| rs4697732 | 4 | 9810971 | T | G | 28021 | -0.1290 | 0.0091 | 9.35E-46 |
| rs2868416 | 4 | 9811672 | T | C | 28007 | -0.1287 | 0.0091 | 1.47E-45 |
| rs10489079 | 4 | 9813061 | C | G | 28013 | 0.1397 | 0.0099 | 1.24E-45 |
| rs6858209 | 4 | 9813255 | A | G | 28005 | 0.1287 | 0.0091 | 1.48E-45 |
| rs9283699 | 4 | 9813287 | T | C | 28006 | -0.1287 | 0.0091 | 1.50E-45 |
| rs10030776 | 4 | 9813573 | C | G | 28007 | 0.1280 | 0.0091 | 4.02E-45 |
| rs10030782 | 4 | 9813594 | T | C | 28007 | -0.1285 | 0.0091 | 2.12E-45 |
| rs9990501 | 4 | 9813691 | A | G | 28020 | 0.1284 | 0.0091 | 2.10E-45 |
| rs10939766 | 4 | 9814068 | A | G | 27967 | 0.1306 | 0.0091 | 1.01E-46 |
| rs16894893 | 4 | 9814693 | A | T | 27849 | -0.0485 | 0.0080 | 1.33E-09 |
| rs231 | 4 | 9814946 | C | G | 27973 | 0.1290 | 0.0091 | 1.04E-45 |
| rs17455117 | 4 | 9815302 | C | G | 26750 | 0.1552 | 0.0138 | 2.98E-29 |
| rs6449355 | 4 | 9815829 | T | C | 27972 | 0.1296 | 0.0091 | 4.29E-46 |
| rs17389602 | 4 | 9816024 | A | T | 27865 | -0.2672 | 0.0108 | 3.90E-135 |
| rs6812780 | 4 | 9817823 | T | G | 27793 | 0.1291 | 0.0092 | 4.28E-45 |
| rs6826450 | 4 | 9817876 | A | G | 27997 | -0.1292 | 0.0091 | 8.74E-46 |
| rs6845818 | 4 | 9817892 | T | C | 27827 | 0.0490 | 0.0080 | 1.04E-09 |
| rs6855489 | 4 | 9817986 | A | G | 27929 | 0.1305 | 0.0091 | 1.31E-46 |
| rs11947517 | 4 | 9818349 | A | G | 27909 | 0.1308 | 0.0092 | 8.89E-46 |
| rs4697977 | 4 | 9819751 | T | C | 27996 | -0.1292 | 0.0091 | 1.10E-45 |
| rs2080072 | 4 | 9844720 | C | G | 27780 | -0.1559 | 0.0083 | 4.72E-79 |
| rs11734783 | 4 | 9849761 | T | C | 27787 | 0.2650 | 0.0119 | 7.40E-110 |
| rs11727366 | 4 | 9853939 | A | G | 27954 | -0.1300 | 0.0090 | 2.27E-47 |
| rs10023177 | 4 | 9854053 | A | T | 27853 | -0.1315 | 0.0090 | 2.18E-48 |
| rs1978274 | 4 | 9854185 | T | G | 27723 | 0.2690 | 0.0108 | 6.18E-137 |
| rs7675945 | 4 | 9854438 | T | C | 27951 | 0.1303 | 0.0090 | 1.55E-47 |
| rs6853056 | 4 | 9854996 | A | G | 27483 | 0.1331 | 0.0091 | 9.22E-49 |
| rs10010656 | 4 | 9855425 | C | G | 27946 | 0.1281 | 0.0090 | 3.46E-46 |
| rs11732042 | 4 | 9855546 | T | C | 27736 | 0.0624 | 0.0104 | 1.86E-09 |
| rs4697983 | 4 | 9856346 | A | G | 19059 | 0.3197 | 0.0354 | 1.61E-19 |
| rs10939801 | 4 | 9856610 | C | G | 27950 | -0.1262 | 0.0091 | 1.71E-43 |
| rs11730940 | 4 | 9856991 | A | G | 27961 | -0.1261 | 0.0091 | 2.98E-43 |
| rs10025702 | 4 | 9857272 | C | G | 27813 | -0.1424 | 0.0099 | 1.16E-46 |
| rs2024282 | 4 | 9858786 | A | G | 14479 | 0.1164 | 0.0119 | 1.12E-22 |
| rs2024281 | 4 | 9858849 | A | G | 27961 | -0.1270 | 0.0092 | 8.84E-44 |
| rs7661555 | 4 | 9859124 | T | C | 27830 | -0.1404 | 0.0100 | 3.30E-45 |
| rs17392044 | 4 | 9859438 | C | G | 27606 | 0.2545 | 0.0130 | 2.43E-85 |
| rs1017124 | 4 | 9859497 | T | C | 27956 | 0.1323 | 0.0090 | 6.98E-49 |
| rs12509424 | 4 | 9859601 | A | G | 27963 | 0.1395 | 0.0099 | 6.89E-45 |
| rs1860896 | 4 | 9859875 | T | C | 27881 | 0.1281 | 0.0090 | 3.45E-46 |
| rs11735543 | 4 | 9860750 | T | G | 26964 | -0.2658 | 0.0112 | 1.68E-124 |
| rs10029208 | 4 | 9861103 | T | G | 27945 | -0.1328 | 0.0090 | 3.72E-49 |
| rs6838644 | 4 | 9861969 | T | C | 27961 | -0.1377 | 0.0099 | 6.28E-44 |
| rs4522862 | 4 | 9862113 | C | G | 27945 | 0.1327 | 0.0090 | 3.86E-49 |
| rs4697984 | 4 | 9863648 | A | C | 27945 | 0.1323 | 0.0090 | 5.17E-49 |
| rs12513165 | 4 | 9865675 | T | C | 27966 | 0.1401 | 0.0099 | 2.83E-45 |
| rs2192101 | 4 | 9867155 | C | G | 27943 | 0.1325 | 0.0090 | 3.58E-49 |
| rs4697986 | 4 | 9868066 | A | G | 27373 | -0.1259 | 0.0090 | 4.12E-44 |
| rs17406107 | 4 | 9872478 | T | G | 27780 | 0.2665 | 0.0107 | 6.32E-137 |
| rs929577 | 4 | 9873986 | A | G | 27980 | 0.1398 | 0.0099 | 4.06E-45 |
| rs759031 | 4 | 9874531 | T | C | 27975 | 0.1341 | 0.0090 | 3.16E-50 |
| rs17472370 | 4 | [9879229](http://www.ncbi.nlm.nih.gov/sites/entrez?term=NT_006316.15&db=nuccore&dopt=graph&m=945489&v=945439:945539&c=3366FF&theme=Details&flip=false&select=null&content=5&color=0&decor=0&layout=0&spacing=0) | A | C | 19063 | -0.2990 | 0.0307 | 2.05E-22 |
| rs10489076 | 4 | 9879946 | T | C | 27890 | 0.0688 | 0.0092 | 8.49E-14 |
| rs12505222 | 4 | 9880234 | T | G | 28017 | -0.0675 | 0.0092 | 2.15E-13 |
| rs7676442 | 4 | 9881689 | T | C | 27975 | -0.0695 | 0.0092 | 5.83E-14 |
| rs10489073 | 4 | 9881886 | A | G | 27962 | 0.0701 | 0.0099 | 1.58E-12 |
| rs17407324 | 4 | 9882141 | A | C | 28020 | 0.0682 | 0.0099 | 5.93E-12 |
| rs10489072 | 4 | 9882342 | T | C | 28024 | 0.0686 | 0.0092 | 8.32E-14 |
| rs10939814 | 4 | 9882427 | T | C | 28030 | 0.0689 | 0.0092 | 6.44E-14 |
| rs10489071 | 4 | 9882647 | A | G | 27908 | -0.0685 | 0.0092 | 9.75E-14 |
| rs2192095 | 4 | 9883271 | T | C | 28031 | 0.0695 | 0.0092 | 4.82E-14 |
| rs4697998 | 4 | 9883724 | T | G | 27981 | -0.0678 | 0.0092 | 1.44E-13 |
| rs4697999 | 4 | 9884032 | C | G | 27971 | -0.0624 | 0.0093 | 1.54E-11 |
| rs17407555 | 4 | 9884092 | A | G | 27991 | -0.0642 | 0.0100 | 1.65E-10 |
| rs1860911 | 4 | 9884155 | T | C | 27771 | 0.1043 | 0.0080 | 3.07E-39 |
| rs1860910 | 4 | 9884568 | T | G | 27761 | -0.1043 | 0.0080 | 3.90E-39 |
| rs10805364 | 4 | 9884616 | A | G | 27796 | 0.2640 | 0.0107 | 2.09E-134 |
| rs6823180 | 4 | 9884929 | T | C | 27832 | -0.0637 | 0.0093 | 7.13E-12 |
| rs6833142 | 4 | 9885080 | A | G | 27592 | 0.0640 | 0.0094 | 9.25E-12 |
| rs10489070 | 4 | 9885450 | C | G | 27943 | 0.2637 | 0.0108 | 1.14E-131 |
| rs12510549 | 4 | 9885565 | T | C | 27915 | 0.2659 | 0.0107 | 2.56E-136 |
| rs4698000 | 4 | 9886565 | T | C | 27951 | 0.0659 | 0.0093 | 1.16E-12 |
| rs6836916 | 4 | 9886890 | C | G | 26818 | 0.0617 | 0.0095 | 7.54E-11 |
| rs16895836 | 4 | 9887447 | T | C | 27666 | -0.0733 | 0.0095 | 1.18E-14 |
| rs7435841 | 4 | 9887650 | A | G | 27444 | -0.0622 | 0.0094 | 3.66E-11 |
| rs10489069 | 4 | 9887766 | C | G | 27877 | 0.1386 | 0.0099 | 1.77E-44 |
| rs10032742 | 4 | 9887991 | A | G | 27963 | 0.0615 | 0.0093 | 3.44E-11 |
| rs4698001 | 4 | 9888511 | A | T | 27960 | 0.0665 | 0.0093 | 7.18E-13 |
| rs17474174 | 4 | 9890509 | T | C | 27793 | -0.1366 | 0.0099 | 5.03E-43 |
| rs16895984 | 4 | 9893825 | T | C | 27923 | 0.1891 | 0.0095 | 5.21E-88 |
| rs4698009 | 4 | 9894091 | T | C | 27942 | 0.2572 | 0.0107 | 1.13E-127 |
| rs4698014 | 4 | 9895399 | T | C | 27883 | -0.2584 | 0.0106 | 2.97E-131 |
| rs17409460 | 4 | 9895525 | C | G | 27982 | 0.1383 | 0.0099 | 2.71E-44 |
| rs7685241 | 4 | 9895763 | A | G | 27997 | -0.1302 | 0.0090 | 1.20E-47 |
| rs10939818 | 4 | 9896060 | T | G | 27813 | -0.1321 | 0.0090 | 2.11E-48 |
| rs17475334 | 4 | 9896268 | A | G | 27996 | -0.1379 | 0.0099 | 4.35E-44 |
| rs11932349 | 4 | 9896349 | A | G | 27886 | 0.1311 | 0.0090 | 4.71E-48 |
| rs10489068 | 4 | 9896366 | A | G | 27998 | 0.1292 | 0.0091 | 1.89E-45 |
| rs17475461 | 4 | 9896775 | T | C | 27937 | -0.0590 | 0.0101 | 4.97E-09 |
| rs11937220 | 4 | 9898972 | T | C | 27986 | 0.1301 | 0.0090 | 1.62E-47 |
| rs7692559 | 4 | 9899824 | A | G | 27973 | -0.0452 | 0.0081 | 2.21E-08 |
| rs6449438 | 4 | 9900161 | T | C | 27938 | 0.0467 | 0.0081 | 7.65E-09 |
| rs2024280 | 4 | 9900911 | T | C | 27782 | 0.2631 | 0.0109 | 1.01E-128 |
| rs2192094 | 4 | 9901066 | T | C | 27991 | -0.1391 | 0.0099 | 7.85E-45 |
| rs11730631 | 4 | 9902066 | A | G | 5873 | 0.2810 | 0.0232 | 7.90E-34 |
| rs11731652 | 4 | 9902082 | T | C | 27196 | 0.2527 | 0.0108 | 4.46E-121 |
| rs917827 | 4 | 9904598 | T | C | 27990 | 0.1278 | 0.0091 | 1.99E-44 |
| rs11728055 | 4 | 9905396 | A | C | 27807 | 0.2247 | 0.0171 | 2.21E-39 |
| rs1860907 | 4 | 9905797 | T | C | 27924 | 0.1296 | 0.0091 | 1.40E-45 |
| rs17410735 | 4 | 9906545 | T | C | 27946 | -0.1405 | 0.0099 | 7.35E-46 |
| rs4697744 | 4 | 9907245 | A | G | 27775 | 0.1268 | 0.0092 | 1.59E-43 |
| rs11732729 | 4 | 9909414 | A | G | 27945 | -0.0589 | 0.0104 | 1.60E-08 |
| rs2080077 | 4 | 9909480 | A | G | 27974 | 0.1324 | 0.0090 | 3.10E-49 |
| rs2098236 | 4 | 9909570 | C | G | 27973 | -0.1326 | 0.0090 | 2.47E-49 |
| rs6834574 | 4 | 9909912 | A | G | 27954 | -0.1400 | 0.0099 | 9.87E-46 |
| rs10939829 | 4 | 9909917 | T | G | 25814 | -0.1359 | 0.0104 | 9.70E-39 |
| rs7683755 | 4 | 9910855 | A | G | 27953 | -0.1403 | 0.0099 | 8.49E-46 |
| rs2868937 | 4 | 9912179 | T | C | 27918 | -0.2609 | 0.0107 | 2.58E-131 |
| rs4698023 | 4 | 9913824 | A | G | 27948 | 0.2599 | 0.0106 | 9.30E-133 |
| rs4698025 | 4 | 9914255 | A | G | 27419 | 0.1327 | 0.0093 | 2.99E-46 |
| rs7689060 | 4 | 9914561 | C | G | 27682 | -0.2603 | 0.0111 | 1.31E-121 |
| rs1468692 | 4 | 9914873 | T | G | 27972 | -0.0453 | 0.0081 | 2.46E-08 |
| rs9991653 | 4 | 9919593 | A | G | 27901 | -0.0449 | 0.0081 | 3.05E-08 |
| rs7436833 | 4 | 9920172 | T | C | 25785 | -0.0938 | 0.0153 | 7.92E-10 |
| rs6449449 | 4 | 9920805 | T | C | 27980 | -0.1199 | 0.0089 | 2.83E-41 |
| rs12511337 | 4 | 9921070 | A | G | 27980 | -0.2604 | 0.0109 | 3.90E-126 |
| rs6449451 | 4 | 9921171 | C | G | 27975 | -0.1200 | 0.0089 | 2.40E-41 |
| rs6449452 | 4 | 9921210 | T | C | 28009 | 0.1199 | 0.0089 | 2.58E-41 |
| rs4698028 | 4 | 9921314 | A | G | 27942 | 0.1199 | 0.0089 | 2.87E-41 |
| rs4698029 | 4 | 9921896 | C | G | 27904 | 0.2586 | 0.0109 | 2.00E-124 |
| rs2192093 | 4 | 9922204 | T | C | 28037 | 0.0483 | 0.0081 | 2.17E-09 |
| rs6810699 | 4 | 9923123 | A | G | 27965 | -0.1209 | 0.0089 | 6.75E-42 |
| rs727995 | 4 | 9923275 | T | G | 28009 | -0.0480 | 0.0080 | 2.21E-09 |
| rs714436 | 4 | 9923765 | A | C | 28030 | 0.2625 | 0.0109 | 3.81E-128 |
| rs2868939 | 4 | 9924019 | A | G | 28037 | 0.0621 | 0.0103 | 1.58E-09 |
| rs17477561 | 4 | 9924194 | T | C | 27930 | -0.1367 | 0.0099 | 1.02E-43 |
| rs4698031 | 4 | 9925019 | A | G | 27993 | 0.2651 | 0.0108 | 4.74E-133 |
| rs6449453 | 4 | 9925932 | A | T | 27926 | -0.1359 | 0.0099 | 3.01E-43 |
| rs17418478 | 4 | 9925949 | A | G | 27626 | -0.2681 | 0.0118 | 2.82E-114 |
| rs7666514 | 4 | 9925977 | A | C | 27764 | 0.1294 | 0.0098 | 5.31E-40 |
| rs17418533 | 4 | 9926039 | T | C | 27984 | 0.2656 | 0.0108 | 1.52E-133 |
| rs6449454 | 4 | 9926479 | A | C | 27980 | -0.1279 | 0.0090 | 9.25E-46 |
| rs11722185 | 4 | 9926580 | C | G | 27986 | 0.2651 | 0.0108 | 4.74E-133 |
| rs1860905 | 4 | 9927934 | C | G | 27986 | -0.1270 | 0.0089 | 7.86E-46 |
| rs11737588 | 4 | 9928105 | A | G | 17547 | -0.2896 | 0.0452 | 1.52E-10 |
| rs4698033 | 4 | 9928669 | T | G | 27999 | -0.1277 | 0.0090 | 1.18E-45 |
| rs4697748 | 4 | 9928797 | T | C | 27998 | 0.1282 | 0.0090 | 5.46E-46 |
| rs993173 | 4 | 9933033 | T | C | 27997 | 0.1213 | 0.0082 | 3.51E-49 |
| rs9291683 | 4 | 9933258 | A | G | 28051 | 0.1027 | 0.0080 | 5.50E-38 |
| rs17478453 | 4 | 9933410 | T | G | 27818 | 0.1403 | 0.0100 | 4.07E-45 |
| rs993172 | 4 | 9933459 | A | C | 27983 | 0.1203 | 0.0082 | 1.20E-48 |
| rs1558489 | 4 | 9934587 | A | T | 27957 | -0.1137 | 0.0082 | 3.97E-44 |
| rs1558488 | 4 | 9936512 | A | G | 27924 | 0.0698 | 0.0093 | 7.04E-14 |
| rs4698036 | 4 | 9940392 | T | G | 27812 | 0.2614 | 0.0106 | 2.85E-134 |
| rs11729318 | 4 | 9946017 | C | G | 27942 | -0.2680 | 0.0108 | 6.20E-136 |
| rs17419612 | 4 | 9946361 | T | G | 27889 | -0.1319 | 0.0098 | 2.20E-41 |
| rs1964268 | 4 | 9947019 | A | T | 27953 | -0.1291 | 0.0090 | 4.67E-47 |
| rs13142790 | 4 | 9948138 | A | G | 27890 | -0.1313 | 0.0098 | 3.05E-41 |
| rs759024 | 4 | 9948193 | A | G | 27962 | -0.1311 | 0.0090 | 4.88E-48 |
| rs2007103 | 4 | 9950353 | A | G | 27976 | 0.1322 | 0.0090 | 2.25E-48 |
| rs4306950 | 4 | 9950517 | T | G | 27927 | 0.1342 | 0.0090 | 9.19E-50 |
| rs984723 | 4 | 9953722 | T | C | 28010 | 0.1284 | 0.0089 | 6.11E-47 |
| rs2052165 | 4 | 9954270 | T | C | 26665 | 0.1006 | 0.0177 | 1.29E-08 |
| rs17420080 | 4 | 9954646 | T | C | 27330 | -0.2648 | 0.0110 | 4.83E-128 |
| rs13109847 | 4 | 9955343 | T | C | 28004 | -0.1374 | 0.0098 | 2.16E-44 |
| rs17479487 | 4 | 9955648 | A | G | 27479 | -0.1434 | 0.0106 | 1.49E-41 |
| rs2192084 | 4 | 9956149 | T | G | 27982 | 0.1317 | 0.0090 | 4.29E-48 |
| rs6849037 | 4 | 9957529 | T | G | 27932 | -0.1335 | 0.0090 | 9.58E-50 |
| rs4698037 | 4 | 9957993 | A | G | 28032 | 0.1343 | 0.0090 | 2.07E-50 |
| rs17420450 | 4 | 9959863 | T | C | 25367 | -0.1571 | 0.0140 | 2.69E-29 |
| rs13145430 | 4 | 9960046 | T | C | 24640 | -0.1317 | 0.0105 | 2.53E-36 |
| rs17420513 | 4 | 9960073 | A | G | 27981 | 0.1403 | 0.0099 | 1.44E-45 |
| rs17420562 | 4 | 9960693 | A | C | 27950 | 0.1385 | 0.0099 | 8.85E-45 |
| rs4697750 | 4 | 9960804 | A | G | 27975 | 0.1324 | 0.0090 | 3.66E-49 |
| rs6853659 | 4 | 9961068 | A | C | 27848 | -0.1369 | 0.0098 | 3.74E-44 |
| rs4697751 | 4 | 9961604 | A | G | 27851 | 0.1321 | 0.0092 | 5.20E-47 |
| rs4698040 | 4 | 9961648 | T | C | 27909 | -0.2674 | 0.0109 | 6.71E-133 |
| rs4697752 | 4 | 9962479 | T | C | 27972 | 0.1326 | 0.0090 | 2.62E-49 |
| rs10938768 | 4 | 9963439 | T | C | 27971 | -0.1327 | 0.0090 | 2.34E-49 |
| rs6838846 | 4 | 9964627 | A | G | 27720 | -0.1238 | 0.0097 | 2.21E-37 |
| rs11931317 | 4 | 9965446 | A | G | 27992 | 0.1303 | 0.0090 | 1.01E-47 |
| rs1860904 | 4 | 9966546 | A | G | 5869 | -0.1277 | 0.0199 | 1.35E-10 |
| rs7680825 | 4 | 9967888 | C | G | 27990 | 0.1327 | 0.0090 | 2.53E-49 |
| rs7681212 | 4 | 9968062 | C | G | 27643 | 0.2355 | 0.0106 | 2.35E-109 |
| rs7661209 | 4 | 9968705 | A | G | 27990 | -0.1327 | 0.0089 | 4.84E-50 |
| rs4697753 | 4 | 9975551 | A | T | 19002 | -0.3307 | 0.0350 | 3.03E-21 |
| rs4698041 | 4 | 9977333 | A | G | 27994 | 0.1326 | 0.0090 | 2.88E-49 |
| rs6857135 | 4 | 9979683 | A | C | 27994 | 0.1326 | 0.0090 | 2.83E-49 |
| rs6813712 | 4 | 9979761 | A | G | 27994 | 0.1326 | 0.0090 | 2.62E-49 |
| rs6849583 | 4 | 9981888 | A | G | 27997 | -0.1324 | 0.0090 | 3.51E-49 |
| rs6851536 | 4 | 9982064 | T | C | 27995 | -0.1325 | 0.0090 | 2.97E-49 |
| rs11943393 | 4 | 9983103 | A | G | 27997 | 0.1324 | 0.0090 | 3.36E-49 |
| rs10938772 | 4 | 9984426 | A | G | 27839 | 0.2363 | 0.0104 | 2.77E-114 |
| rs4698043 | 4 | 9987700 | T | G | 27994 | -0.1323 | 0.0090 | 4.04E-49 |
| rs7677806 | 4 | 9992103 | T | C | 27959 | -0.1314 | 0.0090 | 2.00E-48 |
| rs4302456 | 4 | 9995772 | T | C | 28025 | -0.1380 | 0.0099 | 3.73E-44 |
| rs4302457 | 4 | 9996071 | T | C | 28045 | -0.1378 | 0.0099 | 2.26E-44 |
| rs9990701 | 4 | 9997708 | A | G | 27457 | 0.0804 | 0.0142 | 1.41E-08 |
| rs10017305 | 4 | 10010321 | T | C | 26363 | 0.2253 | 0.0104 | 4.54E-104 |
| rs11943276 | 4 | 10012643 | T | C | 5884 | 0.2398 | 0.0220 | 1.11E-27 |
| rs7654258 | 4 | 10013610 | T | C | 27873 | -0.0903 | 0.0153 | 3.29E-09 |
| rs4463062 | 4 | 10016092 | A | T | 27697 | -0.1213 | 0.0091 | 1.31E-40 |
| rs6819959 | 4 | 10016632 | A | G | 25930 | -0.0480 | 0.0086 | 2.44E-08 |
| rs4643800 | 4 | 10016670 | T | G | 25947 | -0.1993 | 0.0106 | 2.30E-78 |
| rs11728025 | 4 | 10017319 | C | G | 25059 | 0.1912 | 0.0108 | 7.49E-70 |
| rs7697246 | 4 | 10017855 | T | C | 27675 | -0.1227 | 0.0091 | 1.64E-41 |
| rs4698049 | 4 | 10018868 | C | G | 27878 | 0.1226 | 0.0091 | 1.34E-41 |
| rs4698050 | 4 | 10019846 | T | C | 27922 | -0.2218 | 0.0107 | 1.90E-95 |
| rs4610325 | 4 | 10022266 | T | C | 27779 | 0.1225 | 0.0091 | 6.08E-41 |
| rs11736389 | 4 | 10025458 | T | C | 27795 | 0.2288 | 0.0109 | 7.96E-98 |
| rs13125855 | 4 | 10027176 | C | G | 27393 | 0.1058 | 0.0125 | 3.08E-17 |
| rs6858510 | 4 | 10041606 | A | G | 15616 | 0.2971 | 0.0523 | 1.34E-08 |
| rs7691990 | 4 | 10043557 | A | G | 28018 | 0.0792 | 0.0099 | 1.18E-15 |
| rs10003864 | 4 | 10046489 | T | C | 27897 | -0.0482 | 0.0085 | 1.40E-08 |
| rs4422413 | 4 | 10052482 | T | G | 27917 | -0.0577 | 0.0085 | 1.38E-11 |
| rs10938799 | 4 | 10052523 | A | G | 27909 | -0.0898 | 0.0103 | 2.49E-18 |
| rs3217 | 4 | 10053748 | T | C | 27923 | 0.0580 | 0.0085 | 1.13E-11 |
| rs10016022 | 4 | 10056004 | A | G | 28008 | -0.0773 | 0.0099 | 6.06E-15 |
| rs7674156 | 4 | 10061861 | C | G | 27925 | 0.0570 | 0.0085 | 2.37E-11 |
| rs12019277 | 4 | 10064092 | A | C | 27829 | 0.0571 | 0.0086 | 2.55E-11 |
| rs9996284 | 4 | 10078544 | C | G | 23781 | -0.0668 | 0.0099 | 1.30E-11 |
| rs10029818 | 4 | 10081768 | C | G | 27894 | 0.0585 | 0.0086 | 7.96E-12 |
| rs11732503 | 4 | 10091712 | C | G | 27916 | 0.0786 | 0.0096 | 2.79E-16 |
| rs11737650 | 4 | 10094112 | A | G | 27946 | -0.0789 | 0.0100 | 2.31E-15 |
| rs11733306 | 4 | 10097500 | T | C | 27965 | -0.0793 | 0.0099 | 1.48E-15 |
| rs11737601 | 4 | 10097666 | A | G | 23436 | 0.0922 | 0.0099 | 9.61E-21 |
| rs4541501 | 4 | 10097719 | T | C | 27396 | -0.0740 | 0.0101 | 2.13E-13 |
| rs13111270 | 4 | 10098105 | A | T | 27757 | 0.0617 | 0.0086 | 8.56E-13 |
| rs13130674 | 4 | 10098698 | A | G | 27960 | -0.0772 | 0.0099 | 5.56E-15 |
| rs7692088 | 4 | 10100138 | C | G | 27945 | 0.0758 | 0.0082 | 1.76E-20 |
| rs9790491 | 4 | 10102213 | A | G | 28015 | 0.0784 | 0.0098 | 1.78E-15 |
| rs3749558 | 4 | 10103101 | T | C | 28002 | -0.0795 | 0.0099 | 1.05E-15 |
| rs7667644 | 4 | 10104170 | T | C | 28028 | 0.0792 | 0.0099 | 9.81E-16 |
| rs16869060 | 4 | 10104888 | C | G | 27974 | 0.0730 | 0.0092 | 1.97E-15 |
| rs13142053 | 4 | 10105339 | C | G | 25966 | -0.0729 | 0.0099 | 2.30E-13 |
| rs10033825 | 4 | 10105890 | T | C | 27736 | 0.0617 | 0.0086 | 9.02E-13 |
| rs7698826 | 4 | 10106521 | A | C | 27818 | 0.0767 | 0.0082 | 7.41E-21 |
| rs6819820 | 4 | 10107928 | A | G | 27755 | 0.0503 | 0.0084 | 2.40E-09 |
| rs12504795 | 4 | 10108442 | T | C | 27960 | 0.0886 | 0.0097 | 9.92E-20 |
| rs17467273 | 4 | 10109529 | T | C | 27784 | -0.0622 | 0.0087 | 6.72E-13 |
| rs13109939 | 4 | 10110723 | T | C | 27971 | 0.0774 | 0.0099 | 4.97E-15 |
| rs6833095 | 4 | 10115697 | A | G | 27734 | 0.0476 | 0.0085 | 2.03E-08 |
| rs11734599 | 4 | 10116588 | C | G | 27884 | -0.0782 | 0.0099 | 3.42E-15 |
| rs13141385 | 4 | 10117502 | A | C | 27796 | 0.0615 | 0.0085 | 4.86E-13 |
| rs2868941 | 4 | 10118797 | T | C | 27866 | -0.0884 | 0.0098 | 1.32E-19 |
| rs13108998 | 4 | 10119967 | T | G | 27719 | 0.0597 | 0.0085 | 2.36E-12 |
| rs1004327 | 4 | 10120581 | T | C | 27933 | 0.0683 | 0.0094 | 4.47E-13 |
| rs12508358 | 4 | 10121336 | T | C | 27922 | -0.0667 | 0.0094 | 1.28E-12 |
| rs13125670 | 4 | 10122170 | T | C | 27875 | 0.0776 | 0.0081 | 6.82E-22 |
| rs12499142 | 4 | 10123336 | A | T | 27314 | -0.0753 | 0.0100 | 3.96E-14 |
| rs2868942 | 4 | 10123666 | T | C | 27703 | -0.0667 | 0.0095 | 1.94E-12 |
| rs16869379 | 4 | 10123779 | T | C | 27948 | 0.0758 | 0.0093 | 3.13E-16 |
| rs10034180 | 4 | 10127623 | A | G | 27750 | 0.0671 | 0.0083 | 5.80E-16 |
| rs16869430 | 4 | 10128524 | T | C | 27716 | 0.0835 | 0.0082 | 2.69E-24 |
| rs2041215 | 4 | 10129080 | T | G | 27823 | -0.0674 | 0.0083 | 3.17E-16 |
| rs16869474 | 4 | 10130070 | C | G | 27760 | 0.0829 | 0.0082 | 7.31E-24 |
| rs2012249 | 4 | 10130256 | T | C | 25160 | 0.1844 | 0.0296 | 4.35E-10 |
| rs2041216 | 4 | 10132188 | T | C | 27848 | -0.0682 | 0.0082 | 1.30E-16 |
| rs997219 | 4 | 10133769 | A | G | 27885 | -0.0629 | 0.0082 | 1.96E-14 |
| rs2108878 | 4 | 10136440 | T | C | 27864 | 0.0980 | 0.0096 | 2.78E-24 |
| rs12641877 | 4 | 10137324 | A | G | 27943 | 0.0971 | 0.0096 | 5.26E-24 |
| rs2286463 | 4 | 10141356 | A | G | 27685 | 0.0863 | 0.0103 | 5.18E-17 |
| rs2286465 | 4 | 10141732 | A | G | 27663 | 0.0897 | 0.0101 | 9.34E-19 |
| rs10030521 | 4 | 10145515 | C | G | 15616 | -0.2903 | 0.0524 | 3.06E-08 |
| rs10488948 | 4 | 10145642 | A | G | 26848 | -0.0837 | 0.0110 | 2.72E-14 |
| rs10938845 | 4 | 10160598 | T | C | 27481 | 0.0604 | 0.0090 | 1.76E-11 |
| rs1974584 | 4 | 10166959 | T | C | 23399 | 0.0561 | 0.0088 | 1.70E-10 |
| rs1558201 | 4 | 10167016 | C | G | 23128 | 0.0597 | 0.0088 | 1.11E-11 |
| rs2531178 | 4 | 10210232 | A | G | 28030 | -0.0449 | 0.0082 | 4.31E-08 |
| rs2728123 | 4 | 89089819 | T | C | 23584 | -0.0962 | 0.0174 | 3.42E-08 |
| rs11730059 | 4 | 89106351 | A | G | 27935 | 0.1023 | 0.0165 | 5.21E-10 |
| rs2725234 | 4 | 89151296 | T | C | 28006 | 0.0966 | 0.0160 | 1.46E-09 |
| rs2728113 | 4 | 89158760 | A | G | 27958 | -0.0992 | 0.0160 | 5.27E-10 |
| rs2728109 | 4 | 89176747 | A | C | 27958 | 0.1078 | 0.0164 | 5.41E-11 |
| rs2725217 | 4 | 89179282 | A | T | 27617 | -0.1061 | 0.0163 | 6.63E-11 |
| rs2725215 | 4 | 89180595 | T | C | 27936 | 0.1213 | 0.0165 | 2.01E-13 |
| rs2725212 | 4 | 89187737 | A | G | 27705 | -0.0997 | 0.0153 | 6.59E-11 |
| rs2725211 | 4 | 89189399 | T | C | 27934 | 0.1164 | 0.0162 | 6.96E-13 |
| rs2728106 | 4 | 89191075 | A | G | 28031 | 0.0969 | 0.0149 | 7.15E-11 |
| rs2728104 | 4 | 89192030 | T | C | 28026 | -0.1117 | 0.0160 | 2.73E-12 |
| rs2725210 | 4 | 89192451 | A | G | 28020 | -0.0930 | 0.0147 | 2.64E-10 |
| rs2728099 | 4 | 89194762 | T | C | 27931 | -0.1177 | 0.0174 | 1.18E-11 |
| rs2725207 | 4 | 89198553 | A | C | 27843 | 0.0797 | 0.0144 | 3.40E-08 |
| rs2728133 | 4 | 89200714 | T | C | 27836 | -0.0787 | 0.0144 | 4.79E-08 |
| rs2728132 | 4 | 89201485 | A | C | 27837 | -0.0804 | 0.0144 | 2.44E-08 |
| rs2725205 | 4 | 89204735 | A | G | 27770 | 0.0791 | 0.0144 | 4.24E-08 |
| rs4336187 | 4 | 89206940 | A | G | 27589 | -0.0788 | 0.0144 | 4.50E-08 |
| rs2728121 | 4 | 89216126 | T | C | 27801 | 0.0967 | 0.0153 | 2.38E-10 |
| rs2728126 | 4 | 89218246 | A | T | 27807 | 0.0976 | 0.0148 | 4.75E-11 |
| rs2728125 | 4 | 89220917 | A | G | 27905 | -0.1134 | 0.0159 | 9.06E-13 |
| rs2231156 | 4 | 89239451 | A | C | 27921 | 0.1318 | 0.0163 | 6.69E-16 |
| rs4148157 | 4 | 89239958 | A | G | 27923 | 0.1361 | 0.0165 | 1.51E-16 |
| rs4693924 | 4 | 89242248 | A | G | 27888 | 0.1351 | 0.0164 | 1.77E-16 |
| rs2054576 | 4 | 89247799 | A | G | 23470 | -0.1371 | 0.0168 | 2.91E-16 |
| rs12505410 | 4 | 89249865 | T | G | 27297 | 0.0595 | 0.0099 | 2.08E-09 |
| rs2622621 | 4 | 89249944 | C | G | 22950 | -0.0721 | 0.0106 | 1.14E-11 |
| rs1481012 | 4 | 89258106 | A | G | 23592 | -0.1673 | 0.0164 | 2.55E-24 |
| rs2199936 | 4 | 89264355 | A | G | 23541 | 0.1708 | 0.0164 | 2.32E-25 |
| rs2231142 | 4 | 89271347 | T | G | 23622 | 0.1726 | 0.0163 | 3.10E-26 |
| rs4148155 | 4 | 89273691 | A | G | 23610 | -0.1701 | 0.0163 | 1.50E-25 |
| rs3114018 | 4 | 89283605 | A | C | 28020 | -0.0568 | 0.0081 | 2.93E-12 |
| rs2622626 | 4 | 89285739 | A | C | 27853 | 0.0606 | 0.0083 | 2.80E-13 |
| rs17731799 | 4 | 89287479 | T | G | 27500 | 0.0604 | 0.0086 | 2.05E-12 |
| rs2622624 | 4 | 89288430 | T | C | 27492 | -0.0602 | 0.0086 | 2.21E-12 |
| rs2622605 | 4 | 89298410 | T | C | 27579 | -0.0597 | 0.0085 | 2.75E-12 |
| rs3114020 | 4 | 89302690 | T | C | 27432 | -0.0623 | 0.0086 | 4.08E-13 |
| rs10011796 | 4 | 89309901 | T | C | 27434 | 0.0550 | 0.0089 | 5.06E-10 |
| rs10009618 | 4 | 89313032 | T | C | 27663 | -0.0543 | 0.0088 | 7.86E-10 |
| rs1481017 | 4 | 89316501 | T | C | 27620 | -0.0525 | 0.0089 | 4.16E-09 |
| rs150551 | 6 | 25641909 | A | G | 27794 | 0.0527 | 0.0094 | 2.13E-08 |
| rs1028318 | 6 | 25698203 | T | C | 27601 | 0.0534 | 0.0094 | 1.40E-08 |
| rs2077393 | 6 | 25715344 | T | C | 27944 | -0.0528 | 0.0093 | 1.32E-08 |
| rs742132 | 6 | 25715550 | A | G | 27923 | 0.0538 | 0.0093 | 8.50E-09 |
| rs6903765 | 6 | 25717717 | A | G | 27942 | 0.0527 | 0.0093 | 1.55E-08 |
| rs6908390 | 6 | 25717735 | T | C | 27974 | -0.0524 | 0.0093 | 1.83E-08 |
| rs3804105 | 6 | 25720662 | A | C | 27943 | 0.0527 | 0.0093 | 1.53E-08 |
| rs10946785 | 6 | 25725047 | T | G | 27943 | -0.0523 | 0.0093 | 1.98E-08 |
| rs1997672 | 6 | 25725523 | T | G | 27941 | 0.0523 | 0.0093 | 2.00E-08 |
| rs7761700 | 6 | 25726170 | C | G | 27941 | -0.0524 | 0.0093 | 1.89E-08 |
| rs11754288 | 6 | 25884928 | A | G | 27975 | -0.0579 | 0.0084 | 4.38E-12 |
| rs3757131 | 6 | 25891888 | T | C | 27903 | -0.0579 | 0.0083 | 3.67E-12 |
| rs13197601 | 6 | 25893914 | A | G | 27900 | -0.0569 | 0.0084 | 1.11E-11 |
| rs3799344 | 6 | 25894972 | T | C | 27886 | -0.0577 | 0.0083 | 3.53E-12 |
| rs3799346 | 6 | 25899333 | T | C | 27945 | 0.0556 | 0.0101 | 3.82E-08 |
| rs2762353 | 6 | 25902410 | A | G | 27903 | -0.0609 | 0.0082 | 1.19E-13 |
| rs1165215 | 6 | 25906911 | A | G | 27970 | 0.0615 | 0.0082 | 7.12E-14 |
| rs1165209 | 6 | 25909298 | A | G | 27973 | 0.0614 | 0.0082 | 7.81E-14 |
| rs1324082 | 6 | 25909950 | T | C | 27942 | 0.0563 | 0.0101 | 2.34E-08 |
| rs9467604 | 6 | 25912981 | A | T | 27963 | -0.0566 | 0.0101 | 1.96E-08 |
| rs10498730 | 6 | 25920048 | A | G | 27748 | 0.1175 | 0.0203 | 6.97E-09 |
| rs942378 | 6 | 25920438 | A | G | 27973 | 0.0553 | 0.0100 | 3.09E-08 |
| rs1165196 | 6 | 25921129 | A | G | 28042 | 0.0602 | 0.0082 | 2.51E-13 |
| rs7753366 | 6 | 25925497 | A | G | 27984 | 0.0565 | 0.0101 | 1.96E-08 |
| rs1165153 | 6 | 25925768 | A | G | 27997 | -0.0602 | 0.0082 | 2.04E-13 |
| rs1185567 | 6 | 25926567 | A | G | 27999 | -0.0600 | 0.0082 | 2.48E-13 |
| rs1183200 | 6 | 25926625 | C | G | 27999 | 0.0601 | 0.0082 | 2.32E-13 |
| rs12182983 | 6 | 25926734 | A | G | 27905 | 0.0620 | 0.0105 | 3.28E-09 |
| rs1165152 | 6 | 25926745 | A | G | 27982 | -0.0597 | 0.0082 | 3.34E-13 |
| rs6913879 | 6 | 25928407 | T | C | 27974 | -0.0555 | 0.0101 | 3.52E-08 |
| rs1165151 | 6 | 25929595 | T | G | 27905 | -0.0613 | 0.0081 | 4.84E-14 |
| rs3799352 | 6 | 25930599 | T | C | 27905 | 0.0614 | 0.0081 | 4.51E-14 |
| rs1183201 | 6 | 25931423 | A | T | 27908 | -0.0618 | 0.0081 | 3.04E-14 |
| rs6456703 | 6 | 25934098 | T | C | 27945 | 0.0625 | 0.0103 | 1.36E-09 |
| rs1408268 | 6 | 25934965 | A | T | 27955 | 0.0615 | 0.0103 | 2.08E-09 |
| rs1165178 | 6 | 25935495 | A | G | 27910 | -0.0615 | 0.0081 | 4.05E-14 |
| rs765285 | 6 | 25936221 | C | G | 27910 | 0.0614 | 0.0081 | 4.72E-14 |
| rs13200784 | 6 | 25937612 | A | T | 27955 | -0.0610 | 0.0102 | 2.22E-09 |
| rs1165177 | 6 | 25937638 | A | T | 27910 | 0.0610 | 0.0081 | 6.60E-14 |
| rs1165176 | 6 | 25938277 | A | G | 27910 | -0.0615 | 0.0081 | 3.94E-14 |
| rs1185569 | 6 | 25939582 | A | G | 14244 | -0.0582 | 0.0102 | 1.23E-08 |
| rs1185568 | 6 | 25942407 | A | T | 27909 | 0.0614 | 0.0081 | 4.40E-14 |
| rs1184803 | 6 | 25942637 | T | C | 27901 | -0.0614 | 0.0081 | 4.92E-14 |
| rs1185978 | 6 | 25943874 | A | G | 27610 | -0.0582 | 0.0082 | 1.14E-12 |
| rs1165182 | 6 | 25945808 | A | G | 27902 | -0.0616 | 0.0081 | 3.78E-14 |
| rs6905614 | 6 | 25948464 | A | C | 28010 | 0.0575 | 0.0082 | 1.79E-12 |
| rs1408273 | 6 | 25948925 | A | G | 28054 | -0.0598 | 0.0081 | 1.91E-13 |
| rs9393672 | 6 | 25950584 | T | G | 27990 | -0.0614 | 0.0081 | 4.27E-14 |
| rs1165148 | 6 | 25952689 | T | G | 28051 | 0.0635 | 0.0102 | 5.53E-10 |
| rs942379 | 6 | 25957599 | A | G | 27901 | -0.0610 | 0.0082 | 7.99E-14 |
| rs1165189 | 6 | 25957758 | A | C | 28045 | -0.0625 | 0.0102 | 8.20E-10 |
| rs1165187 | 6 | 25959348 | T | C | 28058 | -0.0613 | 0.0101 | 1.46E-09 |
| rs1780969 | 6 | 25966411 | A | C | 27869 | 0.0629 | 0.0101 | 5.66E-10 |
| rs1179087 | 6 | 25966683 | T | G | 27406 | 0.0635 | 0.0107 | 3.46E-09 |
| rs1182814 | 6 | 25967533 | T | C | 28058 | -0.0624 | 0.0102 | 7.90E-10 |
| rs1165167 | 6 | 25968667 | A | G | 28056 | -0.0614 | 0.0101 | 1.25E-09 |
| rs1165165 | 6 | 25970445 | T | C | 27813 | 0.0608 | 0.0104 | 5.71E-09 |
| rs1165164 | 6 | 25971460 | A | G | 28005 | 0.0625 | 0.0109 | 9.45E-09 |
| rs1165162 | 6 | 25971584 | T | C | 28051 | 0.0634 | 0.0102 | 4.83E-10 |
| rs1165161 | 6 | 25972341 | T | C | 27924 | 0.0655 | 0.0102 | 1.09E-10 |
| rs1165160 | 6 | 25972435 | A | G | 27998 | -0.0613 | 0.0081 | 4.44E-14 |
| rs1165159 | 6 | 25972604 | A | G | 28043 | -0.0626 | 0.0102 | 7.28E-10 |
| rs1165158 | 6 | 25972877 | A | C | 28010 | 0.0631 | 0.0102 | 5.58E-10 |
| rs1165207 | 6 | 25973245 | T | C | 28015 | -0.0605 | 0.0081 | 1.01E-13 |
| rs1184804 | 6 | 25976205 | T | C | 27991 | -0.0599 | 0.0081 | 1.60E-13 |
| rs1165205 | 6 | 25978521 | A | T | 28003 | 0.0600 | 0.0081 | 1.60E-13 |
| rs556339 | 6 | 25978724 | T | C | 28013 | 0.0599 | 0.0101 | 3.25E-09 |
| rs9379801 | 6 | 26009690 | T | C | 27995 | -0.0444 | 0.0080 | 3.47E-08 |
| rs199737 | 6 | 26041517 | A | T | 27916 | -0.0563 | 0.0093 | 1.50E-09 |
| rs442601 | 6 | 26042503 | A | G | 27993 | 0.0544 | 0.0092 | 3.06E-09 |
| rs199736 | 6 | 26044766 | T | C | 27998 | 0.0546 | 0.0092 | 2.96E-09 |
| rs9393676 | 6 | 26044923 | A | G | 28022 | 0.0451 | 0.0080 | 1.42E-08 |
| rs9295678 | 6 | 26045012 | A | G | 28022 | 0.0454 | 0.0080 | 1.15E-08 |
| rs199734 | 6 | 26048372 | C | G | 27940 | 0.0552 | 0.0093 | 2.46E-09 |
| rs2051541 | 6 | 26053190 | A | G | 27974 | -0.0491 | 0.0080 | 1.02E-09 |
| rs199726 | 6 | 26061339 | A | G | 27949 | 0.0542 | 0.0092 | 3.42E-09 |
| rs199739 | 6 | 26068488 | A | C | 27943 | -0.0560 | 0.0093 | 1.92E-09 |
| rs129129 | 6 | 26069008 | A | G | 28004 | -0.0547 | 0.0092 | 2.23E-09 |
| rs115810 | 6 | 26083862 | C | G | 27870 | 0.0570 | 0.0093 | 8.92E-10 |
| rs199753 | 6 | 26109867 | A | G | 27855 | 0.0569 | 0.0093 | 8.36E-10 |
| rs199752 | 6 | 26120854 | T | C | 27941 | 0.0558 | 0.0092 | 1.40E-09 |
| rs199751 | 6 | 26123562 | T | C | 27967 | -0.0570 | 0.0092 | 5.53E-10 |
| rs199750 | 6 | 26124441 | T | C | 28043 | 0.0573 | 0.0092 | 4.91E-10 |
| rs9467664 | 6 | 26129792 | A | T | 27825 | -0.0545 | 0.0091 | 2.34E-09 |
| rs9358901 | 6 | 26132415 | T | G | 27861 | 0.0549 | 0.0090 | 1.26E-09 |
| rs1540276 | 6 | 26136798 | T | G | 27994 | -0.0554 | 0.0091 | 9.91E-10 |
| rs2213284 | 6 | 26139847 | A | G | 28002 | 0.0553 | 0.0091 | 1.16E-09 |
| rs2230655 | 6 | 26141485 | A | G | 28019 | 0.0567 | 0.0091 | 4.69E-10 |
| rs4401650 | 6 | 26143187 | A | G | 27983 | 0.0549 | 0.0090 | 1.18E-09 |
| rs1540275 | 6 | 26144455 | T | C | 28001 | -0.0562 | 0.0091 | 6.74E-10 |
| rs2032447 | 6 | 26152348 | A | G | 27394 | -0.0507 | 0.0091 | 2.08E-08 |
| rs7756117 | 6 | 26154544 | A | G | 28009 | 0.0555 | 0.0090 | 7.86E-10 |
| rs10425 | 6 | 26164528 | A | G | 27703 | -0.0503 | 0.0091 | 3.23E-08 |
| rs807214 | 6 | 26169748 | C | G | 27744 | 0.0549 | 0.0089 | 8.43E-10 |
| rs807212 | 6 | 26173600 | A | G | 27791 | -0.0551 | 0.0091 | 1.39E-09 |
| rs1150660 | 6 | 26209419 | A | C | 25815 | -0.0562 | 0.0095 | 2.87E-09 |
| rs198853 | 6 | 26212075 | T | C | 27951 | 0.0564 | 0.0091 | 5.67E-10 |
| rs12356193 | 10 | 61083359 | A | G | 23559 | 0.0779 | 0.0136 | 1.07E-08 |
| rs6591859 | 11 | 64068424 | A | T | 27608 | 0.0463 | 0.0084 | 4.11E-08 |
| rs4930423 | 11 | 64068473 | T | G | 27623 | 0.0469 | 0.0084 | 2.67E-08 |
| rs7124676 | 11 | 64069867 | A | G | 27747 | -0.0592 | 0.0085 | 3.21E-12 |
| rs4930426 | 11 | 64072490 | C | G | 27641 | 0.0599 | 0.0084 | 1.24E-12 |
| rs3759053 | 11 | 64079656 | T | C | 27541 | -0.0602 | 0.0084 | 9.68E-13 |
| rs7940321 | 11 | 64080696 | A | G | 27430 | -0.0597 | 0.0085 | 2.04E-12 |
| rs3782099 | 11 | 64084286 | T | C | 27524 | 0.0599 | 0.0084 | 1.38E-12 |
| rs7943154 | 11 | 64084420 | A | G | 27521 | 0.0600 | 0.0085 | 1.23E-12 |
| rs17300741 | 11 | 64088038 | A | G | 27727 | 0.0616 | 0.0082 | 6.68E-14 |
| rs17372915 | 11 | 64088144 | A | G | 27532 | -0.0601 | 0.0084 | 6.52E-13 |
| rs1783811 | 11 | 64089872 | A | G | 26063 | 0.0521 | 0.0095 | 3.70E-08 |
| rs2078267 | 11 | 64090690 | T | C | 28009 | -0.0614 | 0.0082 | 7.47E-14 |
| rs528211 | 11 | 64108297 | T | C | 27969 | 0.0543 | 0.0093 | 5.49E-09 |
| rs505802 | 11 | 64113648 | T | C | 27967 | -0.0559 | 0.0093 | 2.04E-09 |
| rs10897518 | 11 | 64117281 | T | C | 27962 | -0.0545 | 0.0093 | 5.43E-09 |
| rs7932437 | 11 | 64130080 | T | C | 28046 | 0.0525 | 0.0094 | 2.22E-08 |
| rs7117423 | 11 | 64181696 | T | G | 27937 | 0.0624 | 0.0109 | 9.69E-09 |
| rs544838 | 11 | 64185635 | T | C | 28050 | 0.0509 | 0.0093 | 4.28E-08 |
| rs490192 | 11 | 64227945 | A | G | 27916 | 0.0629 | 0.0106 | 2.97E-09 |
