# Supplementary material for: Meta-Analysis of 28,141 Individuals Identifies Common Variants within Five New Loci That Influence Uric Acid Concentrations
Source: PLoS Genet. 2009 Jun 5;5(6):e1000504. doi: 10.1371/journal.pgen.1000504 (PMC2683940; doi:10.1371/journal.pgen.1000504)
Supplement: Table S5 — Multiple regression analysis. Genome-wide significant SNPs were tested for independent associations, by including all nine SNPs in a multiple regression model, and then performing inverse variance weighted meta-analysis, across participating cohorts (except for Health2000), of the coefficient for each SNP. (0.04 MB DOC) [file pgen.1000504.s008.doc]

| **SNP** | **Single SNP analysis** | | | **Multiple SNP analysis** | | |
| --- | --- | --- | --- | --- | --- | --- |
|  | **beta** | **SE** | **p-value** | **beta** | **SE** | **p-value** |
| rs12129861 | 0.055 | 0.010 | 8.8E-08 | 0.058 | 0.010 | 1.7E-08 |
| rs780094 | 0.053 | 0.009 | 1.1E-08 | 0.054 | 0.009 | 2.0E-09 |
| rs734553 | 0.320 | 0.011 | 1.2E-189 | 0.319 | 0.011 | 9.3E-190 |
| rs2231142 | 0.179 | 0.017 | 1.3E-25 | 0.180 | 0.017 | 5.0E-27 |
| rs742132 | -0.053 | 0.010 | 1.3E-07 | -0.038 | 0.010 | 8.1E-05 |
| rs1183201 | 0.066 | 0.009 | 1.4E-12 | 0.056 | 0.009 | 1.7E-09 |
| rs12356193 | -0.073 | 0.014 | 1.7E-07 | -0.060 | 0.013 | 2.5E-06 |
| rs17300741 | -0.070 | 0.010 | 1.8E-13 | -0.061 | 0.010 | 3.2E-10 |
| rs505802 | -0.063 | 0.010 | 6.4E-10 | -0.040 | 0.010 | 3.6E-05 |
