# Supplementary material for: Meta-Analysis of 28,141 Individuals Identifies Common Variants within Five New Loci That Influence Uric Acid Concentrations
Source: PLoS Genet. 2009 Jun 5;5(6):e1000504. doi: 10.1371/journal.pgen.1000504 (PMC2683940; doi:10.1371/journal.pgen.1000504)
Supplement: Text S1 — Study design. This section describes additional study specific characteristics. (0.14 MB DOC) [file pgen.1000504.s009.doc]

**Study design.** This section describes additional study specific characteristics.

**BRIGHT**

The MRC BRIGHT study (http://www.brightstudy.ac.uk/) comprises 2,500 hypertensive participants and 2,000 normotensive controls of white European ancestry. Case ascertainment and phenotyping has been described previously [1]. Briefly, cases were included if they had blood pressure readings ≥ 150/100 mm Hg based on one reading or ≥ 145/95 mm Hg based on the mean of three readings. Healthy age- and sex-matched normotensive controls (< 140/90 mm Hg) were recruited using the same strict selection criteria. Ethics Committee approval was received from the multi- and local-research committees and all participants gave informed written consent.

**CoLaus**

The Cohorte Lausannoise (CoLaus) is a population-based study aimed at assessing the prevalence and molecular determinants of cardiovascular risk factors in the Caucasian population of Lausanne, Switzerland. The Study was approved by the Institutional Ethic's Committee of the University of Lausanne. Participants in the study (6,188) were randomly selected from the population register of Lausanne in 2003 (n=56,694). The following inclusion criteria were applied: a) written informed consent; b) age 35–75 years and c) Caucasian origin. Caucasian origin was defined as having both parents and grandparents born in a restricted list of European countries. The majority of participants underwent genotying with an Affymetrix 500K array, of which 5,411 have data available for the present analysis.

**CROATIA**

The CROATIA study includes 1031 unselected adult participants, aged 18–93 years, who were recruited in a population-based study during 2003 and 2004 in the villages of Vis and Komiza on the Dalmatian island of Vis. The study received appropriate ethical approval, and all participants gave informed consent. All subjects visited the clinical research center in the region where they were examined in person and where fasting blood was drawn. Biochemical and physiological measurements were performed, detailed genealogies reconstructed, questionnaire of lifestyle and environmental exposures collected, and blood samples stored for further analyses. The settlements on Vis island have complex population histories dating back from the Illyrian period and including periods of isolation and large-scale emigration. A subset of the participants was genotyped genome-wide.

Genotyping was performed using the Illumina HumanHap300 (v1) array, which counts 317,503 SNPs, following the manufacturer’s standard recommendations. Genotypes were determined using BeadStudio software with the recommended parameters for the Infinitum assay and using genotype cluster files provided by Illumina. Samples with a call rate below 97% (for SNP of call rate above 98%, of MAF above 2% and p value for exact test of Hardy Weinberg equilibrium (pHWe) 10-10) were excluded from the analysis using the quality control algorithm implemented in GenABEL which also excluded individuals for which the called gender did not agree with the gender in the study database [2]. Imputation of genotypes was performed on autosomes with the software MACH v1.15 based on HapMap II , after filtering out SNPs with MAF < 0.01, call rate < 98%, and pHWe < 10-6.

**The Health 2000 population**

The population based Health 2000 Survey was carried out in Finland between August 2000 and July 2001. A nationally representative two-stage stratified cluster sample was drawn by stratifying mainland Finland into 20 strata consisting of the 15 biggest cities and five university hospital districts. The 15 cities and 65 out of the 234 municipalities or groups of municipalities with joint primary care (within the five university hospital districts) formed 80 clusters. At stage two, a total of 8028 persons aged 30 years or over were sampled from the clusters. Persons 80 years or over were over sampled by doubling the sampling fraction. Of the 8028 persons 6986 (87%) were interviewed in their home or in an institution, and 6354 (79%) participated a comprehensive health examination. Complete samples with glucose, insulin, HbA1C, HOMA-B and HOMA-IR phenotypes are available from 6302 persons (from 79 % of the original population sample). GWA analysed here was performed on a case control setting on metabolic syndrome (n=2212) on cases being selected according to IDF criterion.

**KORA**

The KORA study is a series of independent population-based epidemiological surveys of participants living in the region of Augsburg, Southern Germany [3]. All survey participants are residents of German nationality identified through the registration office and were examined in 1994/95 (KORA S3) and 1999/2001 (KORA S4). In the KORA S3 study 4,856 subjects (response 75%), and in KORA S4 in total 4,261 subjects have been examined (response 67%). 3,006 subjects participated in a 10-year follow-up examination of S3 in 2004/05 (KORA F3). For KORA F3 we selected 1,644 subjects of these participants while for KORA S4 we randomly selected 1,814 subjects. Informed consent has been given by all participants. The study has been approved by the local ethics committee.

Genotyping for KORA F3 was performed using Affymetrix 500K Array Set consisting of two chips (Sty I and Nsp I). The KORA S4 samples were genotyped with the Affymetrix Human SNP Array 6.0. Hybridisation of genomic DNA was done in accordance with the manufacturer’s standard recommendations. Genotypes were determined using BRLMM clustering algorithm (Affymetrix 500K Array Set) and Birdseed2 clustering algorithm (Affymetrix Array 6.0). For quality control purposes, we applied a positive control and a negative control DNA every 48 (KORA F3) samples or 96 samples (KORA S4). On chip level only subjects with overall genotyping efficiencies of at least 93% were included. In addition the called gender had to agree with the gender in the KORA study database. Imputation of genotypes was performed with the software MACH v1.0.9 (KORA F3) and IMPUTE v0.4.2 (KORA S4) based on HapMap II.

**MICROS**

The MICROS (Micro-isolates in South Tyrol) study is an extensive survey carried out in the period 2001-03 in the villages of Stelvio, Vallelunga, and Martello, located in the Venosta valley (South Tyrol, Italy) [4]. For geographical and political reasons, this German-speaking region, bordering with Austria and Switzerland, experienced prolonged isolation from surrounding populations. Information on the health status of participants was collected through a standardized questionnaire. Laboratory data were obtained from standard blood analyses. Recruitment now amounts to over 1400 individuals. The 1086 participants presented in this study are those for whom both phenotypic and GWA data were available.

Genotyping was performed using the Illumina HumanHap300 (v2) array following the manufacturer’s standard recommendations. Genotypes were determined using BeadStudio software with the recommended parameters for the Infinitum assay and using genotype cluster files provided by Illumina. Samples with a call rate below 98% (for SNP of call rate above 98%, of MAF above 2% and p value for exact test of Hardy Weinberg equilibrium (pHWe) 10^-10^) were excluded from the analysis using the quality control algorithm implemented in GenABEL which also excluded individuals for which the called gender did not agree with the gender in the study database [2]. Imputation of genotypes was performed on autosomes with the software MACH v1.15 based on HapMap II , after filtering out SNPs with MAF < 0.01, call rate < 98%, and pHWe < 10^-6^.

**NSPHS**

The Swedish samples are part of the Northern Swedish Population Health Study (NSPHS) representing a family-based population study including a comprehensive health investigation and collection of data on family structure, lifestyle, diet, medical history and samples for laboratory analyses. Historic population accounts show that there has been little immigration or other dramatic population changes in this area during the last 200 years. Informed consent has been given by all participants and the study has been approved by the local ethics committee.

DNA samples (N=700) were genotyped according to the manufacturer's instructions on using Illumina's HumanHap300 Genotyping BeadChip. Analysis of the raw data was done in the BeadStudio software with the recommended parameters for the Infinium assay and using the genotype cluster files provided by Illumina. Samples with a call rate below 98%, identical twins and genetic outliers were excluded from the analysis, resulting in a total of N = 656 individuals. Approximately 2.5 million SNPs were imputed using release 22 HapMap CEU population as reference. The imputations were performed using MACH software 1.0 (http://www.sph.umich.edu/csg/abecasis/MACH/). Only SNPs with MAF>=0.01 and P HWE >= 1e-6 were used for imputations. All regression models were run using the ProbABEL package from the ABEL set of programs (PMID: 17384015) for the imputed and the measured genotypes [2]. Genomic control was used to correct standard errors of the effect estimates for relatedness among individuals [5].

**ORCADES**

The Orkney Complex Disease Study (ORCADES) is an ongoing family-based, cross-sectional study in the isolated Scottish archipelago of Orkney. Genetic diversity in this population is decreased compared to Mainland Scotland, consistent with the high levels of endogamy historically. Data for participants aged 18-100 years, from a subgroup of ten islands, were used for this analysis. Fasting blood samples were collected and over 200 health-related phenotypes and environmental exposures were measured in each individual. All participants gave informed consent and the study was approved by Research Ethics Committees in Orkney and Aberdeen.

Genotyping was performed using Illumina Hap300 beadchips at the National Research Centre for Environment and Health, Munich, Germany according to the manufacturer’s standard recommendations. Genotypes were called in Beadstudio using clustering files provided by Illumina. Only subjects with overall genotyping efficiencies of at least 97% were included. In addition the called gender had to agree with the gender in the study database. SNPs with call rates below 95% were removed from the analysis. Imputation of genotypes was performed with the software MACH v1.0.15 based on HapMap II.

**PROCARDIS**

PROCARDIS is a European collaborative project studying genetic susceptibility to coronary artery disease (CAD) in Germany, Italy, Sweden and the United Kingdom. Uric acid measurements were available for 1,203 unrelated survivors of myocardial infarction with European ancestry [6]. Informed consent was given by all participants and the study design and protocols were approved by local ethics committees. Genotyping was performed using the Illumina 1M beadchip in two laboratories (CNG, Evry, France and Uppsala, Sweden) and genotypes were called with the BeadStudio software with identical cluster settings. Samples and SNPs with call-rates below 95%, extreme Hardy-Weinberg disequilibrium (p < 5E-7) or minimum allele frequency (MAF) less than 0.5% were excluded from further analysis. Imputation of genotypes was performed using the IMPUTE v0.5.0 program; association analysis with the SNPTEST v1.1.5 program included a country-of-origin categorical covariate.

**SardiNIA**

The SardiNIA study is a population based study, that recruited and phenotyped 6,148 individuals, male and female, ages 14–102 years, from a cluster of four towns in the Lanusei Valley of Sardinia [7]. During physical examination, a blood sample was collected from each individual and divided into two aliquots. One aliquot was used for DNA extraction and the other to characterize several blood phenotypes, including uric acid. Informed consent has been given by all participants. For the GWA scans a total of 4,305 related individuals were examined [8]. Genotyped individuals had four Sardinian grandparents and were selected without regard to their phenotypes.

Genotyping was carried out on 3,329 individuals using Affymetrix 10K and on 1,412 individuals using the Affymetrix 500K, where 436 individuals were genotyped with both arrays. Genotyping was performed according to manufacturer’s protocols and properly assessed by stringent quality control criteria as described elsewhere.[8] Additional genotypes from the entire set of polymorphic HapMap SNPs were imputed using the MACH software. This round of imputation was performed only in 1,412 individuals genotyped with the Affymetrix Mapping 500K Array Set. For the remaining 2,893 individuals genotyped with the Affymetrix Mapping 10K Array, mostly offspring and siblings of the 1,412 individuals that were genotyped with the Affymetrix Mapping 500K Array Set, we took advantage of the relatedness among individuals to impute missing genotypes as described elsewhere [8,9]. Briefly, we identified large stretches of chromosomes shared within each family identical-by-descent and probabilistically “filled-in” genotypes within each stretch whenever one or more of its carriers was genotyped with the 500K Array Set.

**SHIP**

The Study of Health in Pomerania (SHIP) is a cross-sectional survey in West Pomerania, the north-east area of Germany [10]. A sample from the population aged 20 to 79 years was drawn from population registries. First, the three cities of the region (with 17,076 to 65,977 inhabitants) and the 12 towns (with 1,516 to 3,044 inhabitants) were selected, and then 17 out of 97 smaller towns (with less than 1,500 inhabitants), were drawn at random. Second, from each of the selected communities, subjects were drawn at random, proportional to the population size of each community and stratified by age and gender. Only individuals with German citizenship and main residency in the study area were included. Finally, 7,008 subjects were sampled, with 292 persons of each gender in each of the twelve five-year age strata. In order to minimize drop-outs by migration or death, subjects were selected in two waves. The net sample (without migrated or deceased persons) comprised 6,267 eligible subjects. Selected persons received a maximum of three written invitations. In case of non-response, letters were followed by a phone call or by home visits if contact by phone was not possible. The SHIP population finally comprised 4,310 participants (corresponding to a final response of 68.8%).

The SHIP samples were genotyped using the Affymetrix Human SNP Array 6.0. Hybridisation of genomic DNA was done in accordance with the manufacturer’s standard recommendations. The genetic data analysis workflow was created using the Software InforSense. Genetic data were stored using the database Caché (InterSystems). Genotypes were determined using the Birdseed2 clustering algorithm. For quality control purposes, several control samples where added. On the chip level, only subjects with a genotyping rate on QC probesets (QC callrate) of at least 86% were included. The overall genotyping efficiency of the GWA was 98.55 %. Imputation of genotypes in SHIP was performed with the software IMPUTE v0.5.0 based on HapMap II.

**SSAGA**

This cohort is based on Australian twin pairs born before 1964 who participated in questionnaire- and interview-based studies on psychological and metabolic risk factors for psychiatric and cardiovascular disease. 7764 people were interviewed with the Semi-Structured Assessment for Genetics of Alcoholism instrument in 1992-93 (SSAGA Study), and 3375 of them gave blood samples for DNA preparation and serum biochemistry in 1993-96 (SSAGA Blood Study). Recruitment was originally through voluntary registration with the Australian Twin Registry; demographic and metabolic characteristics of the study participants are described elsewhere [11,12]. Monozygotic female twin pairs were initially selected for genotyping. Informed consent was given by all participants and the study has been approved by appropriate ethics committees.

For 477 MZ female twin pairs, selected because both twins had phenotypic information for cholesterol levels, one member of the pair was genotyped. For six of the MZ pairs, both twins and an additional duplicate DNA sample from one of them were genotyped as a quality control measure. The samples were genotyped at the Finnish Genome Center in Helsinki using the Infinium II assay28 on the HumanHap300-Duo Genotyping BeadChips (Illumina Inc, San Diego, USA). Genotyping was performed according to the manufacturers’ instructions (Infinium II assay two-sample manual, #11230506 rev.A, Illumina Inc). In total 318,237 SNPs were genotyped on the BeadChips. The signal intensity data were converted into genotypes using Illumina Beadstudio 2.0 software. Genotypes were assigned using custom defined cluster positions generated from samples genotyped in-house. The quality of samples and reagents was monitored by sample call rates, sample heterozygosity rates and sex. Samples yielding < 95% SNP call rates were discarded. Genotype consistency between the replicated samples corresponded to a reproducibility rate of 99.99%.The final dataset consisted of 318,121 SNPs, and 379 MZ twin-pairs for whom uric acid phenotype data were available.

We imputed genotypes for all 2,621,874 HapMap phase I+II SNPs in the sample using a Hidden Markov Model programmed in MACHv1.0.16 [13,14]. The imputation method combined Illumina HumanHap317 genotype data from our sample with the HapMap CEU sample (July 2006 phased haplotype release r21, http://www.hapmap.org/downloads/phasing/2006-07_phaseII/phased/) and then inferred the unobserved genotypes probabilistically. Association analyses were performed with the PLINK v1.04 genome association analysis toolset using either the observed HumanHap317 genotypes or the MACH best-guess (i.e., most likely) imputed genotypes for each individual at each SNP.[15] Where possible, the mean of the uric acid concentrations from the two MZ twins in each pair was used as the phenotypic value.

**TWINS UK**

The TwinsUK cohort ([www.twinsuk.ac.uk](http://www.twinsuk.ac.uk/)) is an adult twin British registry shown to be representative of singleton populations and the United Kingdom population [16]. A total of 2113 females with acid uric phenotype were included in the analysis. The mean age of the TwinsUK cohort was 47.24(18-79). Ethics approval was obtained from the Guy’s and St. Thomas’ Hospital Ethics Committee. Written informed consent was obtained from every participant to the study.

The design and methodology of the GWA study is described in detail elsewhere [17]. In brief, TwinsUK samples were typed with the Infinium assay (Illumina, San Diego, USA) with three fully compatible SNP arrays. We pooled the normalised intensity data and called genotypes on the basis of the Illluminus algorithm. No calls were assigned if the most likely call was less than a posterior probability of 0.95. Validation of pooling was done by visual inspection of 100 random, shared SNPs for overt batch effects; none were observed.

We excluded SNPs that had a low call rate (≤ 90%), Hardy-Weinberg p values < 10^−4^ and minor allele frequencies < 1%. We, also removed subjects where genotyping failed for >2 % of SNPs. The overall genotyping efficiency of the GWA was 98.7 %. Imputation of genotypes was carried out using the software IMPUTE on two sets of unrelated twins [18].

**References**

1. Caulfield M, Munroe P, Pembroke J, Samani N, Dominiczak A, et al. (2003) Genome-wide mapping of human loci for essential hypertension. Lancet 361: 2118-2123.

2. Aulchenko YS, Ripke S, Isaacs A, van Duijn CM (2007) GenABEL: an R library for genome-wide association analysis. Bioinformatics 23: 1294-1296.

3. Wichmann HE, Gieger C, Illig T (2005) KORA-gen--resource for population genetics, controls and a broad spectrum of disease phenotypes. Gesundheitswesen 67 Suppl 1: S26-S30.

4. Pattaro C, Marroni F, Riegler A, Mascalzoni D, Pichler I, et al. (2007) The genetic study of three population microisolates in South Tyrol (MICROS): study design and epidemiological perspectives. BMC Med Genet 8: 29.

5. Bacanu SA, Devlin B, Roeder K (2000) The power of genomic control. Am J Hum Genet 66: 1933-1944.

6. Broadbent HM, Peden JF, Lorkowski S, Goel A, Ongen H, et al. (2008) Susceptibility to coronary artery disease and diabetes is encoded by distinct, tightly linked SNPs in the ANRIL locus on chromosome 9p. Hum Mol Genet 17: 806-814.

7. Pilia G, Chen WM, Scuteri A, Orru M, Albai G, et al. (2006) Heritability of cardiovascular and personality traits in 6,148 Sardinians. PLoS Genet 2: e132.

8. Scuteri A, Sanna S, Chen WM, Uda M, Albai G, et al. (2007) Genome-wide association scan shows genetic variants in the FTO gene are associated with obesity-related traits. PLoS Genet 3: e115.

9. Sanna S, Jackson AU, Nagaraja R, Willer CJ, Chen WM, et al. (2008) Common variants in the GDF5-UQCC region are associated with variation in human height. Nat Genet 40: 198-203.

10. John U, Greiner B, Hensel E, Ludemann J, Piek M, et al. (2001) Study of Health In Pomerania (SHIP): a health examination survey in an east German region: objectives and design. Soz Praventivmed 46: 186-194.

11. Whitfield JB, Fletcher LM, Murphy TL, Powell LW, Halliday J, et al. (1998) Smoking, obesity, and hypertension alter the dose-response curve and test sensitivity of carbohydrate-deficient transferrin as a marker of alcohol intake. Clin Chem 44: 2480-2489.

12. Heath AC, Bucholz KK, Madden PA, Dinwiddie SH, Slutske WS, et al. (1997) Genetic and environmental contributions to alcohol dependence risk in a national twin sample: consistency of findings in women and men. Psychol Med 27: 1381-1396.

13. Scott LJ, Mohlke KL, Bonnycastle LL, Willer CJ, Li Y, et al. (2007) A genome-wide association study of type 2 diabetes in Finns detects multiple susceptibility variants. Science 316: 1341-1345.

14. Li Y, Abecasis GR (2006) Mach 1.0: Rapid Haplotype Reconstruction and Missing Genotype Inference. Am J Hum Genet S79: 2290.

15. Purcell S, Neale B, Todd-Brown K, Thomas L, Ferreira MA, et al. (2007) PLINK: a tool set for whole-genome association and population-based linkage analyses. Am J Hum Genet 81: 559-575.

16. Spector TD, MacGregor AJ (2002) The St. Thomas' UK Adult Twin Registry. Twin Res 5: 440-443.

17. Richards JB, Rivadeneira F, Inouye M, Pastinen TM, Soranzo N, et al. (2008) Bone mineral density, osteoporosis, and osteoporotic fractures: a genome-wide association study. Lancet 371: 1505-1512.

18. Marchini J, Howie B, Myers S, McVean G, Donnelly P (2007) A new multipoint method for genome-wide association studies by imputation of genotypes. Nat Genet 39: 906-913.

**Appendix A: Wellcome Trust Case Control Consortium Membership and affiliations.**

**Membership of the Wellcome Trust Case Control Consortium (WTCCC):**

**Management Committee:** Paul R Burton^1^, David G Clayton^2^, Lon R Cardon^3^, Nick Craddock^4^, Panos Deloukas^5^, Audrey Duncanson^6^, Dominic P Kwiatkowski^3,5^, Mark I McCarthy^3,7^, Willem H Ouwehand^8,9^, Nilesh J Samani^10^, John A Todd^2^, Peter Donnelly (Chair)^11^

**Analysis Committee:** Jeffrey C Barrett^3^, Paul R Burton^1^, Dan Davison^11^, Peter Donnelly^11^, Doug Easton^12^, David Evans^3^, Hin-Tak Leung^2^, Jonathan L Marchini^11^, Andrew P Morris^3^, I CA Spencer^11^, Martin D Tobin^1^, Lon R Cardon (Co-chair)^3^, David G Clayton (Co-chair)^2^

**UK Blood Services & University of Cambridge Controls**: Antony P Attwood^5,8^, James P Boorman^8,9^, Barbara Cant^8^, Ursula Everson^13^, Judith M Hussey^14^, Jennifer D Jolley^8^, Alexandra S Knight^8^, Kerstin Koch^8^, Elizabeth Meech^15^, Sarah Nutland^2^, Christopher V Prowse^16^, Helen E Stevens^2^, Niall C Taylor^8^, Graham R Walters^17^, Neil M Walker^2^, Nicholas A Watkins^8,9^, Thilo Winzer^8^, John A Todd^2^, Willem H Ouwehand^8,9^

**1958 Birth Cohort Controls:** Richard W Jones^18^, Wendy L McArdle^18^, Susan M Ring^18^, David P Strachan^19^, Marcus Pembrey^18,20^

**Bipolar Disorder (Aberdeen):** Gerome Breen^21^, David St Clair^21^; (**Birmingham):** Sian Caesar^22^, Katherine Gordon-Smith^22,23^, Lisa Jones^22^; **(Cardiff):** Christine Fraser^23^, Elaine K Green^23^, Detelina Grozeva^23^, Marian L Hamshere^23^, Peter A Holmans^23^, Ian R Jones^23^, George Kirov^23^, Valentina Moskvina^23^, Ivan Nikolov^23^, Michael C O’Donovan^23^, Michael J Owen^23^, Nick Craddock^23^; **(London):** David A Collier^24^, Amanda Elkin^24^, Anne Farmer^24^, Richard Williamson^24^, Peter McGuffin^24^; **(Newcastle):** Allan H Young^25^, I Nicol Ferrier^25^

**Coronary Artery Disease (Leeds):** Stephen G Ball^26^, Anthony J Balmforth^26^, Jennifer H Barrett^26^, D Timothy Bishop^26^, Mark M Iles^26^, Azhar Maqbool^26^, Nadira Yuldasheva^26^, Alistair S Hall^26^; **(Leicester):** Peter S Braund^10^, Paul R Burton^1^, Richard J Dixon^10^, Massimo Mangino^10^, Suzanne Stevens^10^, Martin D Tobin^1^, John R Thompson^1^, Nilesh J Samani^10^

**Crohn’s Disease (Cambridge):** Francesca Bredin^27^, Mark Tremelling^27^, Miles Parkes^27^; **(Edinburgh):** Hazel Drummond^28^, Charles W Lees^28^, Elaine R Nimmo^28^, Jack Satsangi^28^; **(London):** Sheila A Fisher^29^, Alastair Forbes^30^, Cathryn M Lewis^29^, Clive M Onnie^29^, Natalie J Prescott^29^, Jeremy Sanderson^31^, Christopher G Mathew^29^; **(Newcastle):** Jamie Barbour^32^, M Khalid Mohiuddin^32^, Catherine E Todhunter^32^, John C Mansfield^32^; **(Oxford):** Tariq Ahmad^33^, Fraser R Cummings^33^, Derek P Jewell^33^

**Hypertension (Aberdeen):** John Webster^34^; **(Cambridge):** Morris J Brown^35^, David G Clayton^2^; **(Evry, France): G** Mark Lathrop^36^; **(Glasgow):** John Connell^37^, Anna Dominiczak^37^; **(Leicester):** Nilesh J Samani^10^; **(London):** Carolina A Braga Marcano^38^, Beverley Burke^38^, Richard Dobson^38^, Johannie Gungadoo^38^, Kate L Lee^38^, Patricia B Munroe^38^, Stephen J Newhouse^38^, Abiodun Onipinla^38^, I Wallace^38^, Mingzhan Xue^38^, Mark Caulfield^38^; **(Oxford):** Martin Farrall^39^

**Rheumatoid Arthritis:** Anne Barton^40^, The Biologics in RA Genetics and Genomics Study Syndicate (BRAGGS) Steering Committee*, Ian N Bruce^40^, Hannah Donovan^40^, Steve Eyre^40^, Paul D Gilbert^40^, Samantha L Hider^40^, Anne M Hinks^40^, Sally L John^40^, Catherine Potter^40^, Alan J Silman^40^, Deborah PM Symmons^40^, Wendy Thomson^40^, Jane Worthington^40^

**Type 1 Diabetes:** David G Clayton^2^, David B Dunger^2,41^, Sarah Nutland^2^, Helen E Stevens^2^, Neil M Walker^2^, Barry Widmer^2,41^, John A Todd^2^

**Type 2 Diabetes (Exeter):** Timothy M Frayling^42,43^, Rachel M Freathy^42,43^, Hana Lango^42,43^, John R B Perry^42,43^, Beverley M Shields^43^, Michael N Weedon^42,43^, Andrew T Hattersley^42,43^; **(London):** Graham A Hitman^44^; **(Newcastle):** Mark Walker^45^; **(Oxford):** Kate S Elliott^3,7^, Christopher J Groves^7^, Cecilia M Lindgren^3,7^, Nigel W Rayner^3,7^, Nicholas J Timpson^3,46^, Eleftheria Zeggini^3,7^, Mark I McCarthy^3,7^

**Tuberculosis (Gambia):** Melanie Newport^47^, Giorgio Sirugo^47^; **(Oxford):** Emily Lyons^3^, Fredrik Vannberg^3^, Adrian VS Hill^3^

**Ankylosing Spondylitis:** Linda A Bradbury^48^, Claire Farrar^49^, Jennifer J Pointon^48^, Paul Wordsworth^49^, Matthew A Brown^48,49^

**AutoImmune Thyroid Disease:** Jayne A Franklyn^50^, Joanne M Heward^50^, Matthew J Simmonds^50^, Stephen CL Gough^50^

**Breast Cancer:** Sheila Seal^51^, Breast Cancer Susceptibility Collaboration (UK)*, Michael R Stratton^51,52^, Nazneen Rahman^51^

**Multiple Sclerosis:** Maria Ban^53^, An Goris^53^, Stephen J Sawcer^53^, Alastair Compston^53^

**Gambian Controls (Gambia):** David Conway^47^, Muminatou Jallow^47^, Melanie Newport^47^, Giorgio Sirugo^47^; **(Oxford):** Kirk A Rockett^3^, Dominic P Kwiatkowski^3,5^

**DNA, Genotyping, Data QC and Informatics (Wellcome Trust Sanger Institute, Hinxton):** Claire Bryan^5^, Suzannah J Bumpstead^5^, Amy Chaney^5^, Kate Downes^2,5^, Jilur Ghori^5^, Rhian Gwilliam^5^, Sarah E Hunt^5^, Michael Inouye^5^, Andrew Keniry^5^, Emma King^5^, Ralph McGinnis^5^, Simon Potter^5^, Rathi Ravindrarajah^5^, Pamela Whittaker^5^, David Withers^5^, Panos Deloukas^5^; **(Cambridge):** Hin-Tak Leung^2^, Sarah Nutland^2^, Helen E Stevens^2^, Neil M Walker^2^, John A Todd^2^

**Statistics (Cambridge):** Doug Easton^12^, David G Clayton^2^; **(Leicester):** Paul R Burton^1^, Martin D Tobin^1^; **(Oxford):** Jeffrey C Barrett^3^, David Evans^3^, Andrew P Morris^3^, Lon R Cardon^3^; **(Oxford):** Niall J Cardin^11^, Dan Davison^11^, Teresa Ferreira^11^, Joanne Pereira-Gale^11^, Ingeleif B Hallgrimsdóttir^11^, Bryan N Howie^11^, Jonathan L Marchini^11^, I CA Spencer^11^, Zhan Su^11^, Yik Ying Teo^3,11^, Damjan Vukcevic^11^, Peter Donnelly^11^

**PIs:** David Bentley^5,54^, Matthew A Brown^48,49^, Lon R Cardon^3^, Mark Caulfield^38^, David G Clayton^2^, Alistair Compston^53^, Nick Craddock^23^, Panos Deloukas^5^, Peter Donnelly^11^, Martin Farrall^39^, Stephen CL Gough^50^, Alistair S Hall^26^, Andrew T Hattersley^42,43^, Adrian VS Hill^3^, Dominic P Kwiatkowski^3,5^, Christopher G Mathew^29^, Mark I McCarthy^3,7^, Willem H Ouwehand^8,9^, Miles Parkes^27^, Marcus Pembrey^18,20^, Nazneen Rahman^51^, Nilesh J Samani^10^, Michael R Stratton^51,52^, John A Todd^2^, Jane Worthington^40^

*^1^ Genetic Epidemiology Group, Department of Health Sciences, University of Leicester, Adrian Building, University Road, Leicester, LE1 7RH, UK; ^2^ Juvenile Diabetes Research Foundation/Wellcome Trust Diabetes and Inflammation Laboratory, Department of Medical Genetics, Cambridge Institute for Medical Research, University of Cambridge, Wellcome Trust/MRC Building, Cambridge, CB2 0XY, UK; ^3^ Wellcome Trust Centre for Human Genetics, University of Oxford, Roosevelt Drive, Oxford OX3 7BN, UK; ^4^ Department of Psychological Medicine, Henry Wellcome Building, School of Medicine, Cardiff University, Heath Park, Cardiff CF14 4XN, UK; ^5^ The Wellcome Trust Sanger Institute, Wellcome Trust Genome Campus, Hinxton, Cambridge CB10 1SA, UK; ^6^ The Wellcome Trust, Gibbs Building, 215 Euston Road, London NW1 2BE, UK; ^7^ Oxford Centre for Diabetes, Endocrinology and Medicine, University of Oxford, Churchill Hospital, Oxford, OX3 7LJ, UK; ^8^ Department of Haematology, University of Cambridge, Long Road, Cambridge, CB2 2PT, UK; ^9^ National Health Service Blood and Transplant, Cambridge Centre, Long Road, Cambridge, CB2 2PT, UK; ^10^ Department of Cardiovascular Sciences, University of Leicester, Glenfield Hospital, Groby Road, Leicester, LE3 9QP, UK; ^11^ Department of Statistics, University of Oxford, 1 South Parks Road, Oxford OX1 3TG, UK; ^12^ Cancer Research UK Genetic Epidemiology Unit, Strangeways Research Laboratory, Worts Causeway, Cambridge CB1 8RN, UK^; 13^ National Health Service Blood and Transplant, Sheffield Centre, Longley Lane, Sheffield S5 7JN, UK; ^14^ National Health Service Blood and Transplant, Brentwood Centre, Crescent Drive, Brentwood, CM15 8DP, UK; ^15^ The Welsh Blood Service, Ely Valley Road, Talbot Green, Pontyclun, CF72 9WB, UK; ^16^ The Scottish National Blood Transfusion Service, Ellen’s Glen Road, Edinburgh, EH17 7QT, UK; ^17^ National Health Service Blood and Transplant, Southampton Centre, Coxford Road, Southampton, SO16 5AF, UK; ^18^ Avon Longitudinal Study of Parents and Children, University of Bristol, 24 Tyndall Avenue, Bristol, BS8 1TQ, UK; ^19^ Division of Community Health Services, St George’s University of London, Cranmer Terrace, London SW17 0RE, UK; ^20^ Institute of Child Health, University College London, 30 Guilford St, London WC1N 1EH, UK; ^21^ University of Aberdeen, Institute of Medical Sciences, Foresterhill, Aberdeen, AB25 2ZD, UK; ^22^ Department of Psychiatry, Division of Neuroscience, Birmingham University, Birmingham, B15 2QZ, UK; ^23^ Department of Psychological Medicine, Henry Wellcome Building, School of Medicine, Cardiff University, Heath Park, Cardiff CF14 4XN, UK; ^24^ SGDP, The Institute of Psychiatry, King’s College London, De Crespigny Park Denmark Hill London SE5 8AF, UK; ^25^ School of Neurology, Neurobiology and Psychiatry, Royal Victoria Infirmary, Queen Victoria Road, Newcastle upon Tyne, NE1 4LP, UK; ^26^ LIGHT and LIMM Research Institutes, Faculty of Medicine and Health, University of Leeds, Leeds, LS1 3EX, UK; ^27^ IBD Research Group, Addenbrooke’s Hospital, University of Cambridge, Cambridge, CB2 2QQ, UK; ^28^ Gastrointestinal Unit, School of Molecular and Clinical Medicine, University of Edinburgh, Western General Hospital, Edinburgh EH4 2XU UK; ^29^ Department of Medical & Molecular Genetics, King’s College London School of Medicine, 8^th^ Floor Guy’s Tower, Guy’s Hospital, London, SE1 9RT, UK; ^30^ Institute for Digestive Diseases, University College London Hospitals Trust, London, NW1 2BU, UK; ^31^ Department of Gastroenterology, Guy’s and St Thomas’ NHS Foundation Trust, London, SE1 7EH, UK; ^32^ Department of Gastroenterology & Hepatology, University of Newcastle upon Tyne, Royal Victoria Infirmary, Newcastle upon Tyne, NE1 4LP, UK; ^33^ Gastroenterology Unit, Radcliffe Infirmary, University of Oxford, Oxford, OX2 6HE, UK^; 34^ Medicine and Therapeutics, Aberdeen Royal Infirmary, Foresterhill, Aberdeen, Grampian AB9 2ZB, UK; ^35^ Clinical Pharmacology Unit and the Diabetes and Inflammation Laboratory, University of Cambridge, Addenbrookes Hospital, Hills Road, Cambridge CB2 2QQ, UK; ^36^ Centre National de Genotypage, 2, Rue Gaston Cremieux, Evry, Paris 91057.; ^37^ BHF Glasgow Cardiovascular Research Centre, University of Glasgow, 126 University Place, Glasgow, G12 8TA, UK; ^38^ Clinical Pharmacology and Barts and The London Genome Centre, William Harvey Research Institute, Barts and The London, Queen Mary’s School of Medicine, Charterhouse Square, London EC1M 6BQ, UK; ^39^ Cardiovascular Medicine, University of Oxford, Wellcome Trust Centre for Human Genetics, Roosevelt Drive, Oxford OX3 7BN, UK; ^40^arc Epidemiology Research Unit, University of Manchester, Stopford Building, Oxford Rd, Manchester, M13 9PT, UK; ^41^ Department of Paediatrics, University of Cambridge, Addenbrooke’s Hospital, Cambridge, CB2 2QQ, UK; ^42^ Genetics of Complex Traits, Institute of Biomedical and Clinical Science, Peninsula Medical School, Magdalen Road, Exeter EX1 2LU UK; ^43^ Diabetes Genetics, Institute of Biomedical and Clinical Science, Peninsula Medical School, Barrack Road, Exeter EX2 5DU UK; ^44^ Centre for Diabetes and Metabolic Medicine, Barts and The London, Royal London Hospital, Whitechapel, London, E1 1BB UK; ^45^ Diabetes Research Group, School of Clinical Medical Sciences, Newcastle University, Framlington Place, Newcastle upon Tyne NE2 4HH, UK; ^46^ The MRC Centre for Causal Analyses in Translational Epidemiology, Bristol University, Canynge Hall, Whiteladies Rd, Bristol BS2 8PR, UK; ^47^ MRC Laboratories, Fajara, The Gambia; ^48^ Diamantina Institute for Cancer, Immunology and Metabolic Medicine, Princess Alexandra Hospital, University of Queensland, Woolloongabba, Qld 4102, Australia; ^49^ Botnar Research Centre, University of Oxford, Headington, Oxford OX3 7BN, UK; ^50^ Department of Medicine, Division of Medical Sciences, Institute of Biomedical Research, University of Birmingham, Edgbaston, Birmingham B15 2TT, UK; ^51^ Section of Cancer Genetics, Institute of Cancer Research, 15 Cotswold Road, Sutton, SM2 5NG, UK; ^52^ Cancer Genome Project, The Wellcome Trust Sanger Institute, Wellcome Trust Genome Campus, Hinxton, Cambridge CB10 1SA, UK; ^53^ Department of Clinical Neurosciences, University of Cambridge, Addenbrooke’s Hospital, Hills Road, Cambridge CB2 2QQ, UK; ^54^ PRESENT ADDRESS: Illumina Cambridge, Chesterford Research Park, Little Chesterford, Nr Saffron Walden, Essex, CB10 1XL, UK.*
